# DNA methylation GrimAge version 2

**DOI:** 10.18632/aging.204434

**Published:** 2022-12-14

**Authors:** Ake T. Lu, Alexandra M. Binder, Joshua Zhang, Qi Yan, Alex P. Reiner, Simon R. Cox, Janie Corley, Sarah E. Harris, Pei-Lun Kuo, Ann Z. Moore, Stefania Bandinelli, James D. Stewart, Cuicui Wang, Elissa J. Hamlat, Elissa S. Epel, Joel D. Schwartz, Eric A. Whitsel, Adolfo Correa, Luigi Ferrucci, Riccardo E. Marioni, Steve Horvath

**Affiliations:** 1 Dept. of Human Genetics, David Geffen School of Medicine, University of California Los Angeles, Los Angeles, CA 90095, USA; 2 San Diego Institute of Science, Altos Labs, San Diego, CA 92121, USA; 3 Population Sciences in the Pacific Program (Cancer Epidemiology), University of Hawaii Cancer Center, University of Hawaii at Manoa, Honolulu, HI 96813, USA; 4 Department of Epidemiology, UCLA Fielding School of Public Health, Los Angeles, CA 90095, USA; 5 Public Health Sciences Division, Fred Hutchinson Cancer Research Center, Seattle, WA 98109, USA; 6 Lothian Birth Cohorts, Department of Psychology, University of Edinburgh, Edinburgh, EH8 9JZ, Scotland, UK; 7 Longitudinal Studies Section, Translational Gerontology Branch, National Institute on Aging, National Institutes of Health, Baltimore, MD 21224, USA; 8 Geriatric Unit, Local Health Unit Tuscany Centre, Firenze, Tuscany 40125, Italy; 9 Dept. of Epidemiology, Gillings School of Global Public Health, University of North Carolina, Chapel Hill, NC 27516-8050, USA; 10 Department of Environmental Health, Harvard T.H. Chan School of Public Health, Boston, MA 02115, USA; 11 Department of Psychiatry and Behavioral Sciences, University of California, San Francisco, CA 94143-0848, USA; 12 Department of Epidemiology, Harvard T.H. Chan School of Public Health, Boston, MA 02115, USA; 13 Dept. of Medicine, School of Medicine, University of North Carolina, Chapel Hill, NC 27599, USA; 14 Departments of Medicine and Population Health Science, Jackson Heart Study, University of Mississippi Medical Center, Jackson, MS 39216, USA; 15 Centre for Genomic and Experimental Medicine, Institute of Genetics and Cancer, University of Edinburgh, Edinburgh, EH4 2XU, Scotland, UK; 16 Dept. of Biostatistics, Fielding School of Public Health, University of California Los Angeles, Los Angeles, CA 90095, USA

**Keywords:** DNA methylation, epigenetic clock, mortality, healthspan

## Abstract

We previously described a DNA methylation (DNAm) based biomarker of human mortality risk *DNAm GrimAge*. Here we describe version 2 of GrimAge (trained on individuals aged between 40 and 92) which leverages two new DNAm based estimators of (log transformed) plasma proteins: high sensitivity C-reactive protein (logCRP) and hemoglobin A1C (logA1C). We evaluate GrimAge2 in 13,399 blood samples across nine study cohorts. After adjustment for age and sex, GrimAge2 outperforms GrimAge in predicting mortality across multiple racial/ethnic groups (meta P=3.6x10^-167^ versus P=2.6x10^-144^) and in terms of associations with age related conditions such as coronary heart disease, lung function measurement FEV1 (correlation= -0.31, P=1.1x10^-136^), computed tomography based measurements of fatty liver disease. We present evidence that GrimAge version 2 also applies to younger individuals and to saliva samples where it tracks markers of metabolic syndrome.

DNAm logCRP is positively correlated with morbidity count (P=1.3x10^-54^). DNAm logA1C is highly associated with type 2 diabetes (P=5.8x10^-155^). DNAm PAI-1 outperforms the other age-adjusted DNAm biomarkers including GrimAge2 in correlating with triglyceride (cor=0.34, P=9.6x10^-267^) and visceral fat (cor=0.41, P=4.7x10^-41^).

Overall, we demonstrate that GrimAge version 2 is an attractive epigenetic biomarker of human mortality and morbidity risk.

## INTRODUCTION

We previously established DNA methylation based (DNAm) GrimAge for predicting mortality risk and showed it outperformed several widely-used DNAm biomarkers of aging [1]. While first generation clocks such as the pan tissue clock (Horvath, 2013) [2] and Hannum et al.’s blood based clock [3] estimate chronological age, second generation clocks estimate mortality risk e.g. the mortality risk score (Zhang et al., 2017 [4]), DNAm PhenoAge (Levine et al. [5], 2018), DNAm GrimAge [1] (2019), and longitudinal data based clocks such as DunedinPoAm [6] and Pace of Aging [7].

Comparative studies in epidemiological cohorts show that DNAm GrimAge often outperforms the aforementioned clocks in terms of a) predicting mortality risk and b) associations with age-related conditions research groups ([8–17]). GrimAge has been used to study many conditions including COVID [13], autism [15], major depression disorder [18], post-traumatic stress disorder (PTSD).

Here we describe a second version of GrimAge, GrimAge2, and demonstrate that it outperforms the original GrimAge with respect to its strength of association with a host of age-related conditions including mortality risk, computed tomography data, cognitive assessments, lifestyle factors, and applicability to saliva. We validate version 2 of GrimAge in almost 13,400 blood samples across nine human cohorts with participants of Hispanic-, European-, and African ancestries.

### Review of version 1 of GrimAge

The first version of GrimAge was defined as a composite biomarker (weighted linear combination) of seven DNAm surrogates of plasma proteins, a DNAm-based estimator of smoking pack-years, age, and sex. GrimAge relied on the fact that some (but not all) plasma protein levels can be estimated based on cytosine methylation levels.

In the following, we denote a DNAm-based surrogate marker by adding the prefix “DNAm” to the respective variable name. To adjust for confounding by chronological age, we define age adjusted measures of DNAm-based variables as the residuals resulting from regressing the DNAm variable on chronological age. For example, we defined the age adjusted version of GrimAge, referred to as age acceleration AgeAccelGrim (in units of year), based on DNAm GrimAge [1]. Thus, a positive (or negative) value of AgeAccelGrim indicates that the DNAm GrimAge is higher (or lower) than expected based on chronological age. We use the same terminology to define AgeAccelGrim2 based on DNAm GrimAge2.

DNAm GrimAge was established based on a two-stage approach [1]. We trained and tested the GrimAge using individuals from the Framingham heart study (FHS) Offspring Cohort [19]. In the first stage, we established DNAm surrogates of plasma proteins as well as smoking pack-years (DNAm PACKYRS). In the second stage, we developed a predictor of mortality by regressing time-to-death due to all-cause mortality (dependent variable) on the following covariates: DNAm surrogates selected from the first stage, chronological age (Age) and sex (Female: 1 indicates females, 0 males), and batch effect as needed.

## RESULTS

### GrimAge version 2

The first version of DNAm GrimAge was defined as a linear combination of chronological age (Age), an indicator of female sex (Female), and eight DNAm biomarkers including DNAm PACKYRS and seven DNAm proteins that are implicated in kidney function, mitochondria dysfunction, inflammation, etc. (Supplementary Note 1). The 1030 unique CpGs underlying version 1 of GrimAge are proximal to genes which play a role in MHC class II, cytokine-mediated signaling pathway and other gene sets from GO, KEGG and PANTHER [1].

We used the same set of 1030 CpGs to construct version 2 of GrimAge. We randomly split the Framingham Heart Study data into training (n=1833) and test (n=711) data (Methods). The mean age of individuals in the training set and test set was 66 and 67 years, respectively. These participants in the training and test datasets have similar demographic profiles and number of years for follow-up (Table 1).

**Table 1:** Overview of the cohorts for validating DNAmGrimAge2.

**Study**	**Race^2^**	**N**		**Female**	**Death**	**Age**	**Follow-up**
		**Samples**	**Subjects**				
**Training data**							
**FHS training**	White	1833	1833	54%	13%	66.1±9.06 [59,73]	7.9±1.67 [7.4,9.89]
**Validation data**							
**FHS test**	White	711	711	54%	14%	66.8±8.62 [61,73]	7.7±1.77 [7.2,8.78]
**WHI BA23**	White	998	998	100%	67%	68.3±6.26 [65,72.77]	19.1±6.22 [15,23.92]
	AfricanA	676	676	100%	52%	63±6.61 [57.9,67.7]	19.5±6.81 [15.7,24.58]
	Hispanic	433	433	100%	43%	62.2±6.87 [56.5,67.5]	20.7±5.78 [18.2,24.48]
**WHI EMPC**	White	1096	1096	100%	48%	64.3±7.1 [58.9,69.79]	21±5.95 [18.2,24.96]
	AfricanA	558	558	100%	45%	62.5±6.98 [57.7,67.46]	21±5.67 [18.8,24.77]
	Hispanic	318	318	100%	30%	61.2±6.21 [56.5,65.96]	22±4.82 [21.9,24.59]
**JHS**	AfricanA	1746	1746	63%	16%	56.2±12.32 [46.5,65.35]	11.7±2.55 [11.2,13.11]
**InCHIANTI**	White	1430	728	56%	37%	67.4±16.17 [61,78]	10±4.87 [5.4,14.58]
**BLSA**	White	572	556	46%	32%	70.9±14.08 [62,82]	6.1±4.18 [2.1,9.32]
**LBC21**	White	692	469	60%	94%	82.3±4.31 [79,86.56]	8.8±5.2 [4.6,12.57]
**LBC36**	White	2796	1044	50%	30%	73.6±3.67 [70.3,76.63]	9.7±4.09 [6,13.05]
**NAS**	White	1373	732	0%	38%	74.5±6.99 [69,79]	10.5±4.71 [6,15]
**Summary^1^**	White, African A, Hispanic	13,399	10,065	71%	39%	67.9±11.33 [61.8,76]	13±6.9 [7.8,16.91]

^1^Summary statistics are based on the nine validation datasets.

^2^AfricanA denotes African American.

The table summarizes the characteristics of 1833 and 13,399 blood samples used in our training and validation process. The training dataset was based on the 1833 individuals from Framingham Heart Study Offspring Cohort (FHS). The validation datasets consist of 10,065 individuals came from nine independent cohorts: FHS test dataset, Women’s Health Initiatives (WHI) BA23, WHI EMPC, Jackson Heart Study (JHS), InCHIANTI (baseline and the third follow-up), Baltimore Longitudinal Study of Aging (BLSA), Lothian Birth Cohort 1921 (LBC21) and LBC 1936 (LBC36), and Normative Aging Study (NAS). The studies cohorts were used in our validation analysis stratified by racial group within each cohort. Age and follow-up time (from methylation profile to last visit/death in units of years) are presented in the format of mean ±SD [25^th^, 75^th^]. Proportion of females are based on individual level.

We started out by developing two new DNAm based estimators of high sensitivity C-reactive protein (CRP) and hemoglobin A1C, respectively. CRP is a widely used biomarker of inflammation while hemoglobin A1C levels are used to assess the short term history of blood glucose levels.

To arrive at DNA methylation based surrogates of these plasma proteins, we used two elastic net regression models to predict log-transformed (base e) versions of high-sensitivity C-reactive protein (log CRP) and hemoglobin A1C (log A1C), respectively. Both elastic net regression models used the following candidate covariates: 1030 CpGs, Age and Female. The two elastic net regression models selected 132 CpGs (for log CRP) and 86 CpGs (for log A1C), respectively (Supplementary Table 1). The predicted values resulting from these regression models will be denoted by DNAm logCRP and DNAm logA1C, respectively. The Pearson correlation coefficients between the DNAm variables and their target proteins are biased in the training dataset (Supplementary Figure 1A, 1B) due to overfitting. Our unbiased analysis in the test dataset leads to the following: Pearson correlations r=0.48 for DNAm logCRP and r=0.34 for DNAm logA1C (Supplementary Figure 1C, 1D).

**Figure 1:**
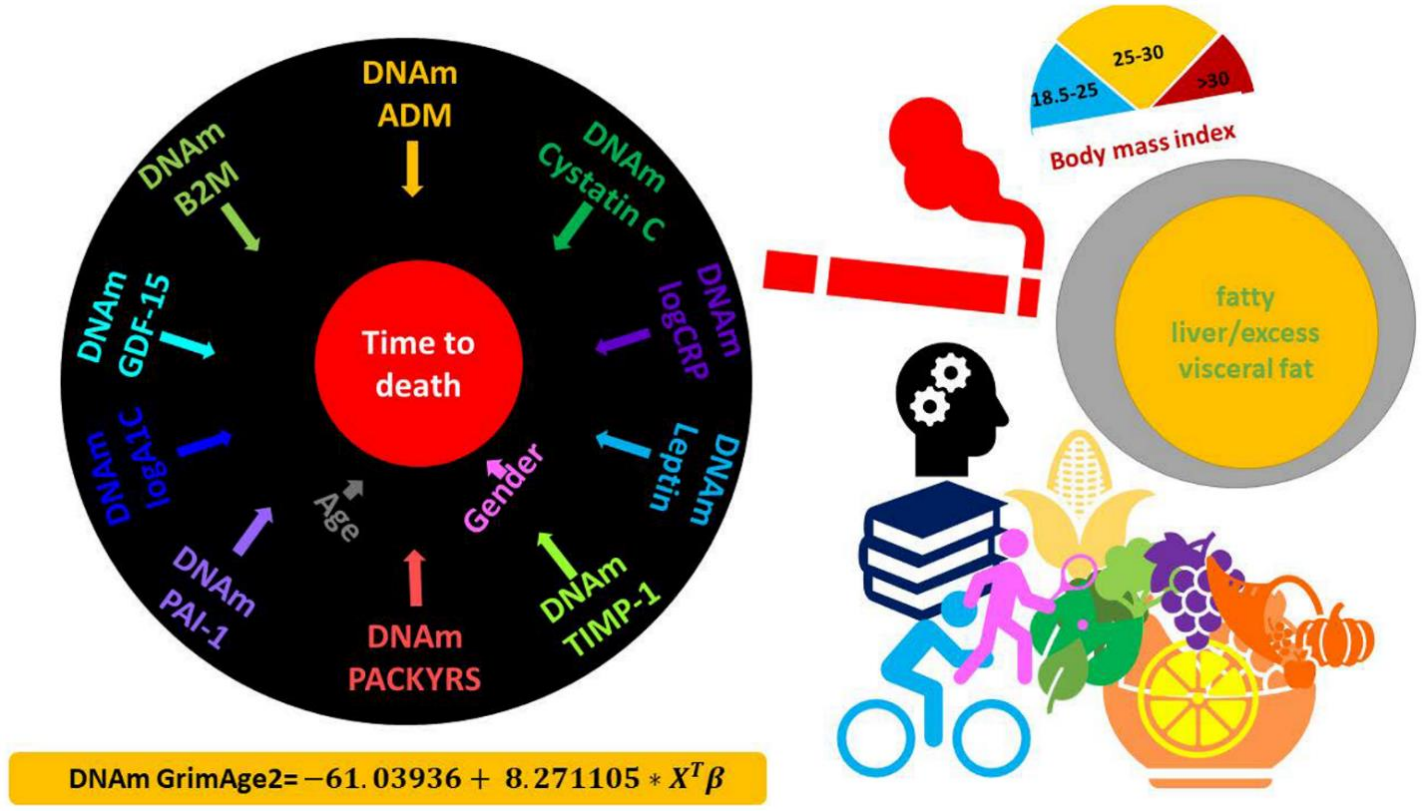
**DNAm GrimAge2.** The left panel displays the components of GrimAge2 trained by Cox regression with an elastic net penalty. The elastic net regression model automatically selected the following covariates: chronological age (Age), gender (Female), and ten DNAm based surrogates for smoking pack-years (DNAm PACKYRS), adrenomedullin levels (DNAm ADM), beta-2 microglobulin (DNAm B2M), cystatin C (DNAm Cystatin C), growth differentiation factor 15 (DNAm GDF-15), leptin (DNAm Leptin), log-scale high sensitivity C-reactive protein (DNAm logCRP), log-scale hemoglobin A1C (DNAm logA1C), plasminogen activation inhibitor 1 (DNAm PAI-1), tissue inhibitor metalloproteinase 1 (DNAm TIMP-1). The linear combination of the covariate values X^T^β was linearly transformed to be in units of years, as described in the bottom. Technically speaking, DNAm GrimAge2 is an epigenetic clock for mortality risk. Metaphorically speaking, it estimates biological age in units of years. The right panel displays selective factors including diet, lifestyle and clinical biomarkers that were significantly associated with age acceleration measure of GrimAge2 or age-adjusted DNAm biomarkers underlying GrimAge2 in our downstream analysis.

To define GrimAge2 we used a Cox regression model to regress time-to-death (due to all-cause mortality) on the following candidate covariates: eleven DNAm-based surrogates of plasma proteins, DNAm PACKYRS, Age, Female (Methods, Supplementary Table 1). We remind the readers that the first version of GrimAge was based on Age, Female, DNAm PACKYRS, and seven DNAm-based proteins: adrenomedullin (ADM), beta-2-microglobulim (B2M), cystatin C (Cystatin C), GDF-15, leptin (Leptin), PAI-1, and tissue inhibitor metalloproteinases 1 (TIMP-1, Supplementary Note 1). Interestingly, the Cox regression model with a elastic net penalty picked up the exactly same seven DNAm proteins, DNAm PACKYRS, as well as the two new biomarkers (DNAm logCRP and DNAm logA1C). Thus, GrimAge2 is based on 12 covariates: 10 DNAm based biomarkers and 2 demographic characteristics: Age, Female (Figure 1). The linear combination of covariates resulting from the elastic net Cox regression model can be interpreted as an estimate of the logarithm of the hazard ratio of mortality. We calibrated this parameter into an age estimate by performing a linear transformation whose slope and intercept terms were chosen by forcing the mean and variance of DNAm GrimAge2 to match that of chronological age in the training data (Figure 1).

### Pairwise correlations between DNAmGrimAge2 and its components

DNAm GrimAge2 correlates positively with its underlying components DNAm GDF15, DNAm TIMP1, DNAm CystatinC, DNAm B2M and chronological age (Pearson correlation r between 0.79 and 0.89, Supplementary Figure 2B). The new DNAm surrogate markers for logCRP and logA1C are positively correlated with DNAm GrimAge2 (r=0.58 and r=0.47) but only weakly with chronological age (r ~0.26). The fact that leptin levels are higher in females [20, 21] explains the strong correlation between DNAm Leptin and Female (r=0.88, Supplementary Figure 2B). Leptin suppresses hunger and is expected to exhibit a negative correlation with mortality/morbidity risk. Indeed, DNAm Leptin exhibits negative correlations with DNAm GrimAge2. The fact that GrimAge2 is defined as a mortality risk predictor explains its high correlation (r=0.42, Supplementary Figure 2B) with the deviance residuals from the Cox proportional hazards model (Methods).

### Independent validation data

We compared the old and new versions of GrimAge in independent validation in datasets consisting of n=13,399 blood samples from 10,065 individuals from nine epidemiological cohorts including the FHS *test* data (Table 1 and Supplementary Note 2). The validation datasets consist of three racial/ethnic groups: 63% European ancestry (72% of all blood samples considered due to repeated measurements), 30% African Americans (22% blood samples) and 7% Hispanic ancestry (6% blood samples). The mean age at blood draw was 67.9 years with a standard deviation of SD=11.33. The mean follow-up time was 13.0 (SD=6.90) years with a mortality rate of 39%. More females (71%) than males were present in our validation data.

To demonstrate that DNAm GrimAge2 is more strongly associated with mortality risk than DNAm GrimAge, we applied both biomarkers to nine different study cohort studies: 1) the test data from the FHS, 2) BA23 and 3)EMPC study from the Women’s Health Initiative (WHI) with three racial groups, 4) African Americans from the Jackson Heart Study (JHS), 5) the InCHIANTI cohort study, 6) individuals of European ancestry from Baltimore Longitudinal Study of Aging (BLSA), 7) Lothian Birth Cohort 1921 (LBC1921) and 8) Lothian Birth Cohort 1936 (LBC1936), and 9) individuals of European ancestry from Normative Aging Study (NAS, only recruiting male participants).

We also applied new and old GrimAge clocks to saliva samples.

### Relation to age

Chronological age is highly correlated with DNAmGrimAge (***r*** ~0.78 to 0.95) and DNAmGrimAge2 (***r*** ~0.72 to 0.94, Supplementary Figure 3) at each cohort except LBC1921 and LBC1936, in which the low correlation estimates reflect that all subjects of the Lothian *Birth* cohorts were born in the same years - either 1921 or 1936 i.e. there is minimal variation in ages in these cohorts. The age correlation is lower with DNAm GrimAge2 compared with DNAm GrimAge, which may reflect the addition of two new variables (DNAm logCRP and DNAm logA1C). Unless indicated otherwise, we used the age-adjusted versions of GrimAge, i.e. the age acceleration measures AgeAccelGrim2 and AgeAccelGrim. The two GrimAge acceleration measures are highly correlated (***r***~0.92 to 0.97, Supplementary Figure 4).

We also defined age-adjusted versions of our DNA-based surrogate markers (for smoking pack-years and the nine plasma protein levels). To interpret the effect size of DNAm protein, we scaled the DNAm based estimators of plasma proteins based on the distributions in the FHS training data (Supplementary Table 1.2), e.g. the scaled version of DNAm logCRP is denoted as s.DNAmlogCRP and one unit of s.DNAmlogCRP denotes one standard deviation of DNAm logCRP.

### Mortality risk analysis

We find that AgeAccelGrim2 is significantly associated with race in both WHI BA23 (Supplementary Figure 5A, Kruskal-Wallis P=4.9x10^-13^) and WHI EMPC (Supplementary Figure 5C, P=4.4x10^-15^). Both cohorts show the same trend: African-Americans have higher values of AgeAccelGrim2 than Hispanics and Caucasians. African-American and Hispanic women are on average 1.7 years (P=1.5x10^-13^) and 0.5 years (P=4.2x10-3) older than Caucasian women according to AgeAccelGrim2 evaluated in the WHI BA23. A similar pattern can be observed for the original version of GrimAge (Supplementary Figure 5B, 5D). We briefly mention that different patterns can be observed for other epigenetic clocks and Caucasians [22].

We find that GrimAge2 outperforms GrimAge across a broad category of lifespan and healthspan related variables as summarized in Table 2.

**Table 2:** Summary of lifespan and healthspan associations with GrimAges.

**Measure**	**Effect size**	**AgeAccelGrim2**	**AgeAccelGrim**	**Location**
**Time-to-death**				
All^1^	Hazard ratio	1.10 (P=3.6e-167)	1.10 (P=2.0e-144)	Figure 2
Smokers^1^	Hazard ratio	1.10 (P=4.2e-104)	1.10 (P=3.0e-91)	Figure 3
Non smokers^1^	Hazard ratio	1.09 (P=4.4e-43)	1.10 (P=8.1e-34)	Figure 3
Adjusted for blood cell composition^2^	Hazard ratio	1.09 (P=5.2e-123)	1.09 (P=1.1e-104)	Supplementary Figure 16
**Time-to-CHD**	Hazard ratio	1.08 (P=4.5e-28)	1.08 (P=2.7e-24)	Figure 4
**Comorbidity**	--	Stouffer’s P=3.0e-27	Stouffer’s P=5.7e-22	Figure 5
**Type 2 diabetes**	Odds ratio	1.07 (P=1.1e-30)	1.05 (P=2.8e-15)	Supplementary Figure 8
**Disease free**	--	Stouffer’s P=7.2e-16	Stouffer’s P=1.1e-10	Supplementary Figure 10
**Mean carotenoids**	bicor	-0.29 (P=8.4e-42)	-0.25 (P=4.5e-32)	Figure 6
**log2(C-reactive protein)**	bicor	0.32 (P=9.9e-276)	0.26 (P=6.2e-178)	Figure 6
**FEV1**	bicor	-0.31 (P=1.1e-136)	-0.29 (P=2.1e-119)	Figure 6
**log2 (Waist/hip ratio)**	bicor	0.23 (P=3.9e-69)	0.18 (P=3.6e-45)	Figure 6
**Current smoker**	bicor	0.35 (P=4.5e-299)	0.36 (P=1.1e-363)	Figure 6
**Liver attenuation (Hounsfield unit)**	bicor	-0.27 (P=1.18e-14)	-0.24 (P=2.79e-10)	Figure 7
**Visceral adipose tissue (CM^3^)**	bicor	0.22 (P=7.15e-12)	0.20 (P=2.75e-12)	Figure 7
**HOMA-IR^3^**	bicor	0.16 (5.27e-04)	0.14 (9.74e-03)	Figure 8
**Granulocyte**	Pearson correlation	0.29 (P=1.2e-232)	0.22 (P=1.1e-126)	Supplementary Figure 15
**CD4+T**	Pearson correlation	0.26 (P=3.7e-192)	-0.22 (P=6.1e-126)	Supplementary Figure 15

^1^refers to all and stratified by smoking group.

^2^refers to Cox regression models additionally adjusted for blood cell composition/count variables.

^3^GrimAge models were applied to saliva methylation data.

The table briefly summarizes our investigations for the associations of GrimAge2 and GrimAge with (1) mortality analysis across all validation datasets, stratification of smoking status, and specific Cox regression models adjusted for blood cell composition/counts and (2) a broad category of healthspan outcomes. The columns list names of mortality or healthspan related outcomes, type of effect size in association analysis, summary statistics in the format of effect size (meta P value) or Stuffer’s P value for AgeAccelGrim2 and AgeAccelGrim, and the location of corresponding figure. Abbreviation: bicor denotes a robust correlation coefficient (biweight midcorrelation [26]).

All of our statistical analyses adjusted for obvious confounders such as racial/ethnic group, age, sex, or batch of data generation (e.g. in the LBC1936, Methods). We applied fixed effects meta analysis models (weighted by inverse variance) to combine the results for predicting all-cause mortality risk (time-to-death) from a total of 15 strata formed within the nine epidemiological cohorts.

**Figure 2:**
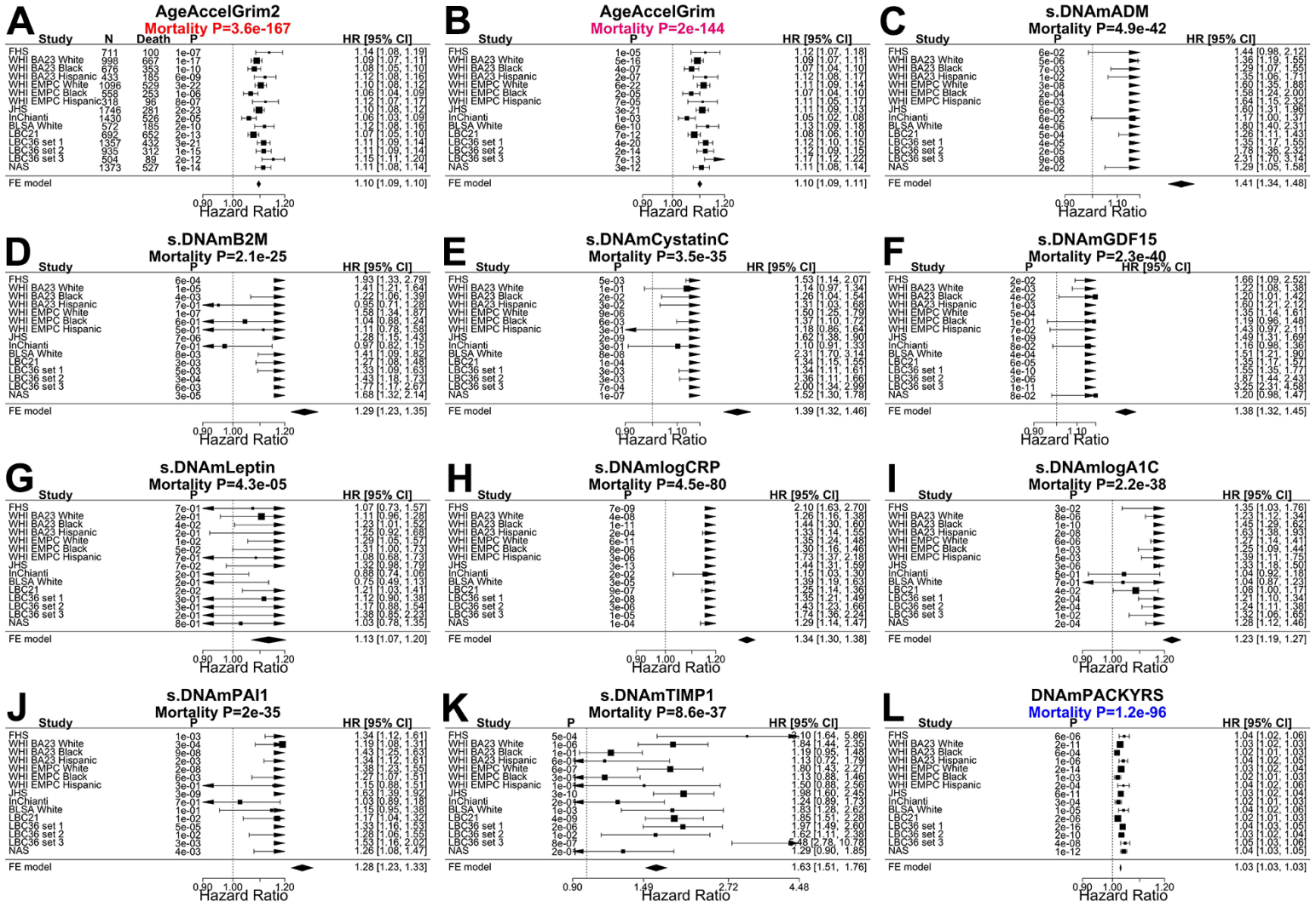
**Meta analysis forest plots for predicting time-to-death due to all-cause mortality.** Fixed effect meta analysis was performed to combine mortality analysis across 15 strata from 9 study cohorts: FHS test data, Women’s Health Initiatives (WHI) BA23, WHI EMPC, Jackson Heart Study (JHS), InCHIANTI (baseline and the third follow-up), Baltimore Longitudinal Study of Aging (BLSA), Lothian Birth Cohort 1921 (LBC21) and LBC 1936 (LBC36), and Normative Aging Study (NAS). Each panel reports a meta analysis forest plot for combining hazard ratios predicting time-to-death based on a DNAm based biomarker (reported in the figure heading) across different strata formed by racial group within cohort and set within LBC36. (**A**, **B**) display the results for AgeAccelGrim2 and AgeAccelGrim. Each row reports a hazard ratio (for time-to-death) and a 95% confidence interval resulting from a Cox regression model in each of 15 strata. (**C**–**L**) display the results for (age-adjusted) DNAm based surrogate markers of (**C**) adrenomedullin (ADM), (**D**) beta-2 microglobulin (B2M), (**E**) cystatin C (Cystatin C), (**F**) growth differentiation factor 15 (GDF-15), (**G**) leptin, (**H**) log scale of C reactive protein (CRP), (**I**) log scale of hemoglobin A1C, (**J**) plasminogen activation inhibitor 1 (PAI-1), (**K**) tissue inhibitor metalloproteinase 1 (TIMP-1) and (**L**) smoking pack-years (PACKYRS). The sub-title of each panel reports the meta analysis P-value. (**A**, **B**) Each hazard ratio (HR) corresponds to a one-year increase in AgeAccel. (**C**–**K**) Each hazard ratio corresponds to an increase in one-standard deviation. (**L**) Hazard ratios correspond to a one-year increase in pack-years. The most significant meta analysis P-value is marked in red (AgeAccelGrim2), followed by hot pink (AgeAccelGrim) and blue (DNAm PACKYRS), respectively.

**Figure 3:**
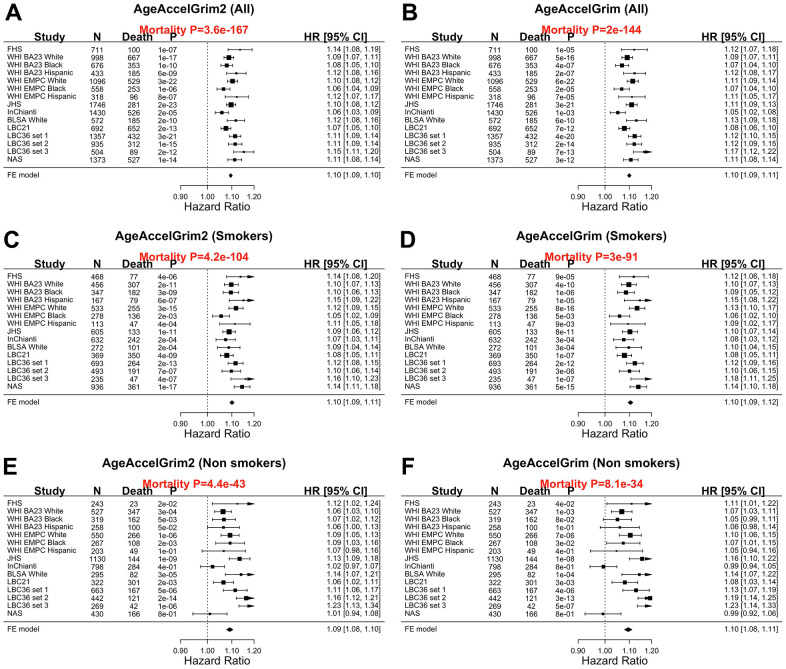
**Meta analysis forest plots for predicting all-cause mortality in all, smokers and non-smokers.** Fixed effect models meta analysis was performed to combine mortality analysis across 15 strata from 9 study cohorts. Analysis was performed across different strata formed by racial groups within cohort and set within LBC36, using (**A**, **B**) all individuals, (**C**, **D**) smokers (former and current), and (**E**, **F**) non-smokers, respectively. Each panel reports a meta-analysis forest plot for combining hazard ratios predicting time-to-death based on AgeAccelGrim2 (on the left panel) and AgeAccelGrim (on the right panel). The sub-title of each panel reports the meta analysis P-value. Each hazard ratio (HR) corresponds to a one-year increase in AgeAccel measure.

Our meta-analysis shows that AgeAccelGrim2 (meta P-value=3.6x10^-167^ for AgeAccelGrim2, Figure 2A) is a more significant predictor of time-to-death (due to all-cause mortality) than the original AgeAccelGrim (meta P-value=2.6x10^-144^ for AgeAccelGrim, Figure 2B). The same applies when the analysis is restricted to former/current smokers (Figure 3C, 3D), never-smokers (Figure 3E, 3F), or specific racial groups. For instance, in postmenopausal African American women from the WHI BA23 study, a one-year increase in age acceleration is associated with a hazard ratio HR=1.08 (Cox regression P=1.0x10^-10^) for AgeAccelGrim2, which is more significant than that for AgeAccelGrim (HR=1.07, P=4.0x10^-7^, Figure 2A, 2B). The improvements of version 2 can be observed in all strata except for data set 2 from LBC1936. However, the two versions of GrimAge work almost equally well in this exception once the analysis is stratified by smoking status (Figure 3C–3F). In particular, a one-year increase in AgeAccelGrim2 (P=4.0x10^-7^) and AgeAccelGrim (P=3.0x10^-6^) are associated with the same hazard ratio (HR=1.10) for mortality risk in data set 2 of LBC1936 (Figure 3C, 3D).

### Heart disease and time to cancer

We also compared the two versions of GrimAge with respect to predicting incident time-to-coronary heart disease (time-to-CHD), time to congestive heart failure (time-to-CHF). After adjustment for age, sex, race, batch, Cox regression models revealed that AgeAccelGrim2 has more significant associations with time-to-CHD (P-values: 4.5x10^-28^ vs 2.7x10^-24^, Figure 3A, 3B) and time to congestive heart failure (P-values: 4.2x10^-15^ vs 6.9x10^-10^, Supplementary Figure 6). Both versions of GrimAge lead to similar Cox regression p-values in predicting time-to-any cancer (meta P-values: 1.1x10^-10^, vs 5.6x10^-10^, Supplementary Figure 7).

### Comorbidity index and healthspan

**Figure 4:**
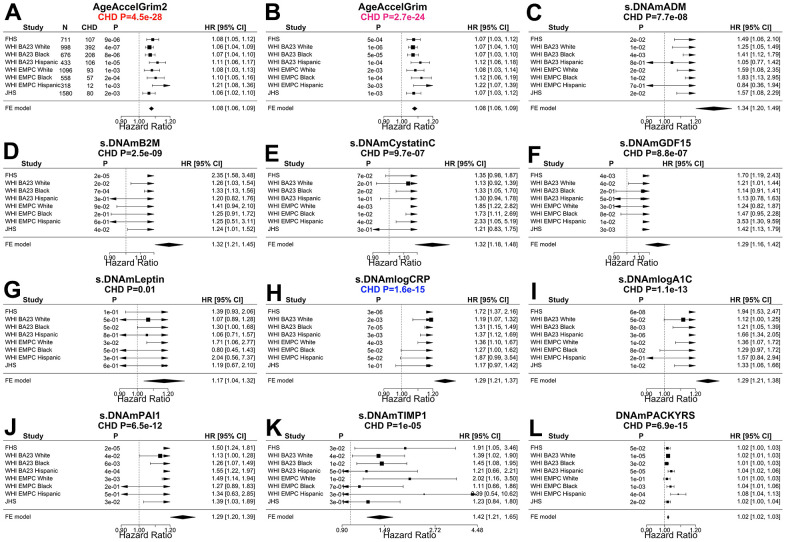
**Meta analysis forest plots for predicting time-to-coronary heart disease.** Fixed effect models meta analysis was performed to combine Cox regression analysis of coronary heart disease (CHD) across 8 strata from 4 study cohorts. Each panel reports a meta analysis forest plot for combining hazard ratios predicting time-to-CHD based on a DNAm based biomarker (reported in the figure heading) across different strata formed by racial groups within the cohort. (**A**, **B**) Results for AgeAccelGrim2 and AgeAccelGrim. Each row reports a hazard ratio (for time-to-CHD) and a 95% confidence interval resulting from a Cox regression model in each strata. (**C**–**L**) display the results for (age-adjusted) DNAm based surrogate markers of (**C**) adrenomedullin (ADM), (**D**) beta-2 microglobulin (B2M), (**E**) cystatin C (Cystatin C), (**F**) growth differentiation factor 15 (GDF-15), (**G**) leptin, (**H**) log scale of C reactive protein (CRP), (**I**) log scale of hemoglobin A1C, (**J**) plasminogen activation inhibitor 1 (PAI-1), (**K**) tissue inhibitor metalloproteinase 1 (TIMP-1) and (**L**) smoking pack-years (PACKYRS). The sub-title of each panel reports the meta analysis P-value. (**A**, **B**) Each hazard ratio (HR) corresponds to a one-year increase in AgeAccel. (**C**–**K**) Each hazard ratio corresponds to an increase in one-standard deviation. (**L**) Hazard ratios correspond to a one-year increase in pack-years. The most significant Meta analysis P-value is marked in red (AgeAccelGrim2), followed by hot pink (AgeAccelGrim) and blue (DNAm logCRP), respectively.

AgeAccelGrim2 greatly outperforms AgeAccelGrim when it comes to associations with a comorbidity index (defined as the total number of age-related conditions, Methods): Stouffer meta analysis P=3.0x10^-37^ for AgeAccelGrim2 versus P=5.7x10^-22^ for AgeAccelGrim, Figure 4A, 4B). The superior performance of GrimAge2 can also be observed when focusing on individual age-related conditions: type 2 diabetes (meta P values: 1.1x10^-30^ versus 2.8x10^-15^, odds ratios [OR]: 1.07 vs 1.05, Supplementary Figure 8A, 8B), hypertension status (meta P values: 8.8x10^-20^ versus P=2.2x10^-13^, OR: 1.05 vs 1.04, Supplementary Figure 9A, 9B), disease free status (meta P=7.2x10^-16^ versus P-value=1.1x10^-10^, Supplementary Figure 10A, 10B) and physical functioning level (meta P=2.0x10^-26^ versus P=1.3x10^-17^, Supplementary Figure 11A, 11B).

### Age at menopause

We have previously shown that age at menopause in women is negatively associated with epigenetic age acceleration [23, 24]. Here we performed the regression analysis of epigenetic age acceleration measures (as dependent variables) on age at menopause (as an independent variable) and potential confounders. We found that both AgeAccelGrim2 and AgeAccelGrim were higher on average for those with an earlier age at menopause. One year earlier in age at menopause was associated with 0.08 additional years of AgeAccelGrim2 (meta P-value=5.4x10^-16^) and 0.07 years of AgeAccelGrim (meta P-value=8.5x10^-16^, Supplementary Figure 12A, 12B).

### DNAm estimates of CRP, A1C, and PAI-1

**Figure 5:**
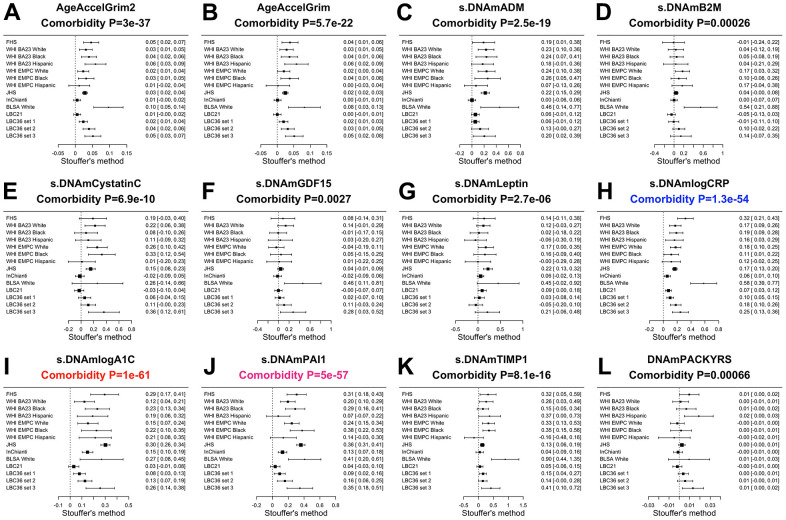
**Meta analysis of associations with total number of age-related conditions.** Each panel reports a meta analysis forest plot based on Stouffer’s method for combining regression analysis Z statistics between the comorbidity index and the DNAm-based biomarker (reported in the figure heading) across different strata, which are formed by racial group within cohort and set within LBC36. (**A**, **B**) display the results for AgeAccelGrim2 and AgeAccelGrim. (**C**–**L**) display the results for scaled DNAm based surrogate markers of (**C**) adrenomedullin (ADM), (**D**) beta-2 microglobulin (B2M), (**E**) cystatin C (Cystatin C), (**F**) growth differentiation factor 15 (GDF-15), (**G**) leptin, (**H**) log scale of C reactive protein (CRP), (**I**) log scale of hemoglobin A1C, (**J**) plasminogen activation inhibitor 1 (PAI-1), (**K**) tissue inhibitor metalloproteinase 1 (TIMP-1) and (**L**) smoking pack-years (PACKYRS). The sub-title of each panel reports the meta analysis p-value. Each row reports a beta coefficient β and a 95% confidence interval resulting from a (linear-mixed) regression model in each strata (defined by cohort racial group). (**A**, **B**) Each β corresponds to a one-year increase in AgeAccel. (**C**–**K**) Each β corresponds to an increase in one-standard deviation. (**L**) β corresponds to a one-year increase in pack-years. The most significant meta-analysis P-value is marked in red (DNAm logA1C), followed by hot pink (DNAm PAI1) and blue (DNAm logCRP), respectively.

Our previous study revealed that DNAm PAI-1 (plasminogen activator inhibitor 1) is associated with a host of age-related conditions [1]. Here we show that the two new DNAm biomarkers DNAm logCRP and DNAm logA1C exhibit comparable patterns with many age-related conditions. These three DNAm based surrogates of plasma proteins are sometimes superior to AgeAccelGrim2 for their strength of association with age-related traits such as the comorbidity index: Stouffer P-value=1.0x10^-61^ for DNAm logA1C, P=1.3x10^-54^ for DNAm logCRP, P=5.0x10^-57^ for DNAmPAI-1, and P=3.0x10^-37^ for AgeAccelGrim2 (Figure 5). Compared to AgeAccelGrim2, these three biomarkers show stronger positive associations with type 2 diabetes (led by DNAm logA1C: meta P-value =5.8x10^-155^, Supplementary Figure 8), hypertension (led by DNAm PAI-1: meta P-value=5.8x10^-43^, Supplementary Figure 9), and disease free status (led by DNAm logCRP: meta P-value=4.0x10^-21^ but not in DNAm logA1C, Supplementary Figure 10).

Lower values of DNAm logCRP (meta P-value=6.5x10^-33^) and AccelGrim2 (meta P-value=2.0x10^-26^, Supplementary Figure 10) are associated with higher levels of physical functioning. These three age-adjusted DNA based biomarkers of plasma proteins are also associated with time-to-CHD (Figure 4), time-to-CHF, time-to-any cancer, and early age at menopause (Supplementary Figures 7, 8, 11) but P values are higher (i.e. less statistically significant) than those observed for AgeAccelGrim2 with one exception: time to CHF where age-adjusted DNAm logCRP (P=6.0x10^-16^) and AgeAccelGrim2 (4.2x10^-15^, Supplementary Figure 6) show comparable associations.

### GrimAge analysis of diet and clinical biomarkers

Here we revisit the cross sectional relationships between GrimAge and dietary variables, clinical biomarkers, educational attainment [1, 25].

Our previous cross sectional analysis was based on approximately n=4000 postmenopausal women from the WHI. Here we greatly increased the sample size to n=13,420 blood samples from nine validation datasets. In total, we investigated 61 variables including 27 self-reported diet, 9 dietary biomarkers based on blood samples, and 19 clinical biomarkers for vital signs, metabolic traits, and markers of inflammation, cognitive function, lung function, anthropometric traits (Methods and Supplementary Table 2.1).

We correlated our DNAm based biomarkers with clinical plasma based biomarkers for inflammation/infection including interleukin 6 in plasma [IL-6], tumor necrosis factor [TNFA]), lung function (forced expiratory volume in one second [FEV1]), and cognitive function based on Mini–Mental State Examination (MMSE).

We also investigated oral supplements (vitamins, selenium, etc.) and biomarkers of aging such as leukocyte telomere length (LTL) and hand grip strength.

**Figure 6:**
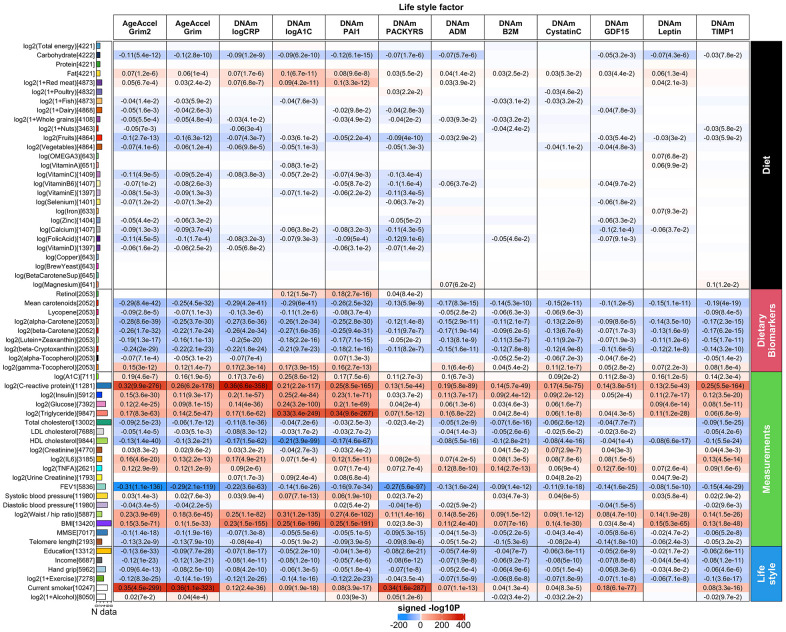
**Meta cross-sectional correlations with diet, clinical biomarkers and lifestyle factors.** Robust correlation coefficients (biweight midcorrelation [26]) between 1) AgeAccelGrim2, AgeAccelGrim, and ten age-adjusted underlying DNAm-based surrogate biomarkers underlying DNAmGrimAge2, and 2) 61 variables including 27 self-reported diet, 9 dietary biomarkers, 19 clinically relevant measurements related to vital signs, metabolic traits, inflammatory markers, cognitive function, lung function, central adiposity and leukocyte telomere length, and 6 lifestyle factors including hand grip strength. The y-axis lists variables in the format of name (sample size), followed by a bar plot denoting number of studies. Variables are arranged by category displayed on the right annotation. The x-axis lists AgeAccelGrim2, AgeAccelGrim, followed by DNAm estimates of log CRP, log A1C, PAI-1, smoking pack-years, etc. Each cell presents meta bicor estimates and P-value, provided P<0.1. The color gradient is based on -log10 P-values times sign of bicor. P-values are unadjusted. An analogous analysis stratified by gender can be found in Supplementary Figure 12.

We used a robust correlation test (biweight midcorrelation bicor) that is less sensitive to outlier data points [26]. Our analysis was stratified by sex and racial group within each cohort. The results of different strata were meta-analyzed using the inverse variance weighted fixed effects models (Methods, Figure 6 and Supplementary Tables 2.2–2.13). In general, AgeAccelGrim2 has more significant associations than AgeAccelGrim (Figure 6 and Supplementary Tables 2.2–2.13). Inflammation biomarkers such as CRP levels showed stronger positive correlations with AgeAccelGrim2 (meta bicor=0.32, P-value=9.9x10^-276^) than with AgeAccelGrim (meta bicor=0.26 and P-value= 6.2x10^-178^, Figure 6). Body fat distribution measures such as waist to hip ratio showed stronger positive correlation with AgeAccelGrim2 (meta bicor=0.23 and P-value=3.9x10^-69^) than with AgeAccelGrim (meta bicor=0.18 and P-value=3.6x10^-45^, Figure 6). Similarly, measures of lipid, insulin or glucose metabolism (triglyceride, HDL, hemoglobin A1C, insulin and glucose), TFNA, IL-6, plasma creatinine and body mass index (BMI) had stronger associations with AgeAccelGrim2 than AgeAccelGrim. AgeAccelGrim2 correlates with lung functioning (FEV1: meta bicor= -0.31, P-value=1.1x10^-136^), brain functioning (mini mental state exam [MMSE]: meta bicor=-0.10, P-value=1.4x10^-18^), leukocyte telomere length (LTL: meta bicor= -0.13, P-value=3.2x10^-9^) and hand grip strength (meta bicor= -0.09, P-value=6.4x10^-13^, Figure 6). The original measure of AgeAccelGrim exhibits weaker correlations with these variables except for LTL (meta bicor= -0.13, P-value=7.9x10^-10^ for AgeAccelGrim). FEV1 shows strong correlation with (age-adjusted) DNAm PACKYRS (meta bicor= -0.27 and P-value=5.6x10^-97^); however, it has even stronger associations with AgeAccelGrim2 (meta bicor= -0.31 and P-value=1.1x10^-136^) and AgeAccelGrim (meta bicor= -0.29 and P-value=2.1x10^-119^, Figure 6).

The identified associations with dietary variables and lifestyle factors are in general more significant for AgeAccelGrim2 than for AgeAccelGrim. AgeAccelGrim2 correlates negatively with plasma based biomarkers measuring vegetable consumption including mean carotenoid levels (meta bicor=-0.29, P-value=8.4x10^-42^, Figure 6). Far less significant associations could be observed for self-reported measures of fruit- and vegetable intake, which highlights the limitations of self-reported measures of dietary intake. AgeAccelGrim2 was inversely related to (self-reported) proportion of carbohydrate, fruit/vegetable consumption, and various supplements including calcium, vitamin C, and folic acid. AgeAccelGrim2 was positively related to self-reported fat intake but with protein intake.

Lastly, higher levels of education and income are associated with lower AgeAccelGrim2.

### DNAm plasma proteins versus diet and clinical biomarkers

All (age-adjusted) DNAm-based biomarkers correlated with a large number of variables across the diet and clinical biomarker outcome categories (Figure 6 and Supplementary Tables 2.3–2.12). Age-adjusted DNAm PAI-1, DNAm logCRP and DNAm logA1C and DNAm PACKYRS stand out. Insulin, glucose and triglyceride are more strongly associated with DNAm PAI-1 or DNAm logA1C than with AgeAccelGrim2. For example, triglyceride levels have a positive correlation with DNAm PAI-1 (meta bicor=0.34 and P-value=9.6x10^-267^) that is double the magnitude of its association with AgeAccelGrim2 (meta bicor=0.17 and P-value=8.3x10^-63^). As expected, the highest correlation with CRP is DNAm logCRP (meta bicor=0.36 and P-value=6.6x10^-358^) and the highest correlation with A1C is DNAm logA1C (meta bicor=0.25 and P=8.6x10^-12^). As noted, the latter one was only based on 711 individuals from FHS test data. The analysis stratified by sex can be found in Supplementary Figure 13.

### Computed tomography measures of fatty organs

Computed tomography imaging techniques provide “shadow images of fat” that can be used for the indirect quantification of organ quality (e.g. liver). Radiographic pixels measure the density of an organ (referred to as attenuation) in Hounsfield units (HU). Computed tomography scans are used for diagnosing fatty liver disease: a low density/attenuation value (low HU) is associated with *high* fat content in the liver. Previously, we analyzed CT scan data from liver, spleen, paraspinal muscle, visceral adipose tissue (VAT), and subcutaneous adipose tissue (SAT) from FHS [27, 28]. Volumetric measures of adipose tissue are also available for SAT and VAT volume measures (in units of CM^3^). With the exception of muscle, CT values exhibit only weak correlations with chronological age (Supplementary Figure 14).

**Figure 7:**
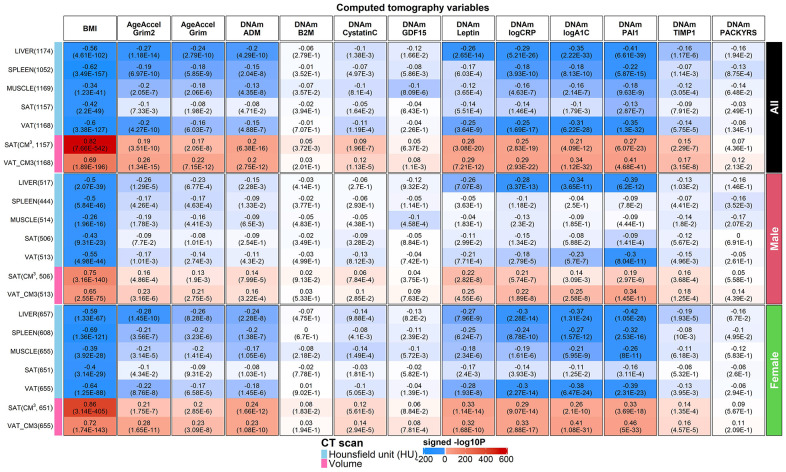
**Computed tomography variables versus BMI and age-adjusted DNAm biomarkers in the FHS.** Robust correlation coefficients (biweight midcorrelation [26]) between 1) AgeAccelGrim2, AgeAccelGrim, and ten age-adjusted DNAm-based surrogate biomarkers underlying DNAmGrimAge2, and 2) seven computed tomography-derived organ density measures (Hounsfield units) or volumetric measures for subcutaneous adipose tissue (SAT CM^3^) or visceral adipose tissue (VAT CM^3^). The y-axis lists computed tomography variables in the format of name (sample size in FHS), annotated by variable type. The x-axis lists body mass index (BMI), AgeAccelGrim2, AgeAccelGrim, followed by DNAm variables in alphabetical order. Each cell presents bicor (P-value). P-values are unadjusted and reported based on linear mixed analysis with pedigree as random effect to avoid confounding by pedigree structure. The color gradient is based on -log10 P-values times sign of bicor. We applied the correlation analysis to males and females, respectively, and then combined the results via fixed effect models weighted by inverse variance (listed in the top rows, denoted as “ALL”). The heatmap presents the results based on ALL and stratification results by gender, annotated on the right side.

Previously, we showed that AgeAccelGrim and DNAm PAI-1 were strongly associated with CT-derived measures of adiposity [1]. Here we revisit this analysis using GrimAge2 (Figure 7).

We find that AgeAccelGrim2 outperforms AgeAccelGrim when it comes to associations with CT-derived measures of adiposity in both genders (Figure 7). For example, both AgeAccelGrim2 and AgeAccelGrim are negatively correlated with liver density (bicor= -0.27 [P=1.18x10^-14^] and bicor=-0.24 [P=2.79x10^-10^]) and positively correlated with VAT volume (bicor=0.26 [P=1.34x10^-15^] and bicor=0.22 [P=7.15x10^-12^], Figure 7).

The strong marginal correlations between AgeAccelGrim2 and CT measures are not confounded by BMI or sex as can be seen by several multivariate regression models that regressed AgeAccelGrim2 (dependent variable) on BMI, sex, and several CT derived measures of organ density and fat volume (Methods, Models I-IV in Supplementary Table 3.1). Even after adjusting for potential confounders, AgeAccelGrim2 exhibits a significant association with liver density (P=5.3x10^-6^), spleen density (P=0.04) but not muscle density (P=0.17 in Model I in Supplementary Table 3.1). A multivariate model analysis, which adjusts for sex, age, and BMI reveals that AgeAccelGrim2 exhibits more significant associations for VAT volume (P= 7.5x10^-6^) than SAT, which supports the widely held view that VAT is more dangerous than SAT. AgeAccelGrim2 is more sensitive to volumetric measures of VAT (in units of cm^3^ and P=2.1x10^-3^) compared to density based VAT (in units of HU, P>0.9, Supplementary Table 3.1). A comprehensive multivariate model (Model IV) that includes both organ density measures and volumetric measures of SAT/VAT reveals that liver density (P=2.6x10^-4^) exhibits the most significant association with AgeAccelGrim2. All multivariate regression models show that BMI is no longer associated with AgeAccelGrim2 after adjusting for liver density, which suggests that liver density mediates the relationship between BMI and AgeAccelGrim2 (Supplementary Table 3.2).

Age-adjusted DNAm-based surrogate markers of PAI-1, exhibit the strongest associations with the CT measures, followed by the surrogates of our two new proteins, A1C and CRP (Figure 7). These three DNAm based proteins outperform AgeAccelGrim2 when it comes to the association with CT-derived measures of adiposity (liver fat and measures of SAT and VAT in Figure 7 and Supplementary Tables 3.1, 3.3–3.5). For example, DNAm PAI1 is highly significantly associated all the CT measures including positive correlations with VAT volume (*r*=0.41, P=4.68x10^-41^) and SAT volume (*r*=0.27, P=6.07x10^-23^), and negative correlations with liver density (*r*=-0.41, P=6.61x10^-39^), VAT density (*r*=-0.35, P=1.3x10^-32^), and spleen density (*r*=-0.22, P=5.87x10^-15^, Figure 7). A multivariate regression analysis of age-adjusted PAI-1 (dependent variable) reveals highly significant associations with liver density (P=6.3x10^-16^ in Model I) and VAT volume (P=1.0x10^-13^, Model II in Supplementary Table 3.3) even after adjusting for BMI and other confounders. Including all CT variables as covariates in a multivariate model reveals significant associations with liver density (P=1.40x10^-9^), VAT volume (P=9.3x10^-8^), and SAT volume (P=0.02, Model IV in Supplementary Table 3.3). Model III shows that DNAm PAI1 is more associated with VAT and SAT in volume measures than with density measures (Supplementary Table 3.3). Similar results were observed for DNAm logCRP but not DNAm logA1C (Supplementary Tables 3.4, 3.5). Our analysis shows that DNAm logA1C is more significant related to SAT density (P=3.0x10^-5^) than to SAT volume (P>0.4) and similar statements apply to VAT density (P=8.0x10^-5^) and VAT volume (P=8.0x10^-3^, Supplementary Table 3.5).

Finally, the surrogates of ADM, TIMP-1, leptin exhibit relatively weak correlations with the CT based measures (Figure 7).

Overall, our results suggest that fatty liver and excess VAT are the most significant CT-based correlates of (age-adjusted) DNAm PAI-1, DNAm logCRP, DNAm logA1C and AgeAccelGrim2.

### Association with blood cell composition

DNAm data allow one to estimate several quantitative measures of blood cell types (both proportions and counts) as described in Methods [22, 29]. We previously showed that AgeAccelGrim and several age-adjusted DNAm biomarkers underlying GrimAge exhibited significant correlations with these imputed measures of blood cell composition. Not surprisingly, AgeAccelGrim2 and AgeAccelGrim exhibit similar patterns for their associations with blood cell composition (Supplementary Figures 15–17 and Supplementary Tables 4.1–4.3). The current results are based on a much larger sample size (n>11,600 across our validation datasets) than our previous study (n ~6000). AgeAccelGrim2 was positively correlated with a DNAm based estimates of granulocytes (*r*=0.29, P=1.2x10^-232^, Supplementary Figure 15A, 15B and Supplementary Table 4.1), plasma blasts (*r*=0.26, P=3.7x10^-181^) and negatively correlated with CD4+T cells (*r* = -0.26, P=3.7x10^-192^) and CD8 naïve cells (*r* = -0.22, P=2.5x10^-135^).

Similar to our previous findings, age-adjusted DNAm TIMP-1 exhibits the most significant correlations with the measures of blood cell composition (e.g. proportion of granulocytes *r*=0.40, P=2.1x10^-495^, Supplementary Figure 15K). The TIMP-1 protein plays a role in promoting cell proliferation in a wide range of cell types and may also have an anti-apoptotic function [30]. Significant associations can also be observed for age-adjusted DNAm logCRP (proportion of granulocytes *r*=0.36, P=5.8x10^-384^), and age-adjusted DNAm Cystatin C (proportion of CD4+ T cells counts *r* =-0.29, P=1.3x10^-231^). By contrast, age-adjusted DNAm A1C is not associated with blood cell composition (Supplementary Figure 15I).

The improved performance of AgeAccelGrim2 compared to AgeAccelGrim1 does not reflect confounding by blood cell composition as can be seen from our multivariate Cox regression models that adjusted for seven imputed measures of blood cell counts or proportions (Supplementary Figure 16). AgeAccelGrim2 (P=5.2x10^-123^) still outperforms AgeAccelGrim when it comes to the association with time-to-death (P=1.1x10^-104^, Supplementary Figure 16A, 16B) after adjusting for blood cell composition. The same can be observed when predicting time-to-CHD where AgeAccelGrim2 (P=1.2x10^-20^) outperforms AgeAccelGrim (P=9.2x10^-18^, Supplementary Figure 17A, 17B). A one standard deviation increase in DNAm logA1C or in DNAm logCRP approximately increases the hazard ratio for CHD by 30% (Figure 4H, 4I). This increased HR is only lowered by 2% (from 1.29 to 1.28 for DNAm logCRP and from 1.29 to 1.27 for DNAm logA1C) after adjusting for blood cell counts (Supplementary Figure 17H, 17I).

Stratifying the analysis by sex indicates that our results are not sex-specific (Supplementary Figures 18, 19 and Supplementary Tables 4.2, 4.3).

### Evaluation of younger individuals

Next, we examined the performance of GrimAge clocks on younger individuals (age < 40) using 173 individuals (minimum at 22 and mean age at 35.4 years) from JHS. As expected, AgeAccelGrim2 was still associated with age-related biomarkers including inflammation marker CRP (r=0.26 and P=5.5x10^-4^), dyslipidemia marker triglyceride levels (r=0.23 and P=2.8x10^-3^), and body mass index (r=0.25 and P=9.1x10^-4^, Supplementary Figure 20B–20D). AgeAccelGrim2 is also associated with life style factors such as alcohol assumption (r=0.33 and P=1.3x10-5, Supplementary Figure 20E) and smoking (P=6.0x10^-7^, Supplementary Figure 20E). The associations remain significant even after adjusting for age and gender in multivariate regression analysis (Supplementary Figure 20). However, DNAmGrimAge2 is not aligned with chronological age in younger individuals. Rather, it exhibits a systematic offset resulting in a median absolute error (MAE) of 11 years (Supplementary Figure 20A). The offset was lower for the original DNAmGrimAge (MAE=4.14 years, Supplementary Figure 20G). However, the original AgeAccelGrim showed less significant associations with all age-related conditions (Supplementary Figure 20H–20K) except for smoking.

### GrimAge clocks can be applied to saliva samples

We applied both versions of GrimAge to saliva samples from n=432 mothers from the NHLBI Growth and Health Study (NGHS) cohort [31]. The cohort was a longitudinal study conducted from 1985 to 2000 that studied various factors related to the development of obesity in pre-adolescents (Methods, Supplementary Note 2). Our methylation samples were profiled in saliva from two racial groups: 50% White (n=218) and 50% Black (n=214). The ages of the mothers ranged from 36 to 43 years. The low age correlation estimates with DNAm GrimAge2 (*r*=0.13) and DNAm GrimAge (*r*=0.17) reflect the relatively narrow age range (Supplementary Figure 21).

**Figure 8:**
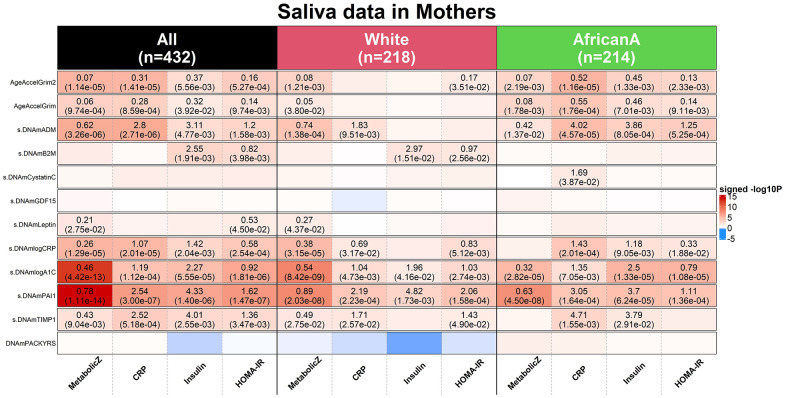
**Applications of DNAm GrimAges on saliva methylation data in NGHS.** DNAmGrimAge, DNAmGrimAge2 and its components were estimated in saliva methylation data from mothers. Linear regression analysis was performed to study the association between 1) dependent variables: clinically relevant measures: metabolic Z score, high sensitivity C-reactive protein (CRP), insulin resistant and HOMA for insulin resistance (HOMA-IR) [32] and 2) independent variables: AgeAccelGrim2, AgeAccelGrim, and nine scaled DNAm-based surrogates of proteins and DNAmPACKYRS. Regression models were performed in all mothers (n=432) and stratified by ethnic/racial groups: White (n=218) and African American (n=214). Analysis was adjusted for age and batch effect and adjusted for race as needed. The y-axis lists DNAm-based variables and the x-axis lists the clinically relevant measures. Each cell presents beta coefficient (P-value), provided P< 0.05 from the regression analysis. The color gradient is based on -log10 P-values times sign of beta coefficient. All P-values are unadjusted.

The mean value of DNAmGrimAge2 was 61.6 years which indicates that there is a systematic offset between blood and saliva sample (Supplementary Figure 21A). Systematic offsets can be adjusted for by using multivariate regression models that include an intercept term. Our multivariate linear regression analysis revealed significant associations between saliva based AgeAccelGrim2 (independent variable) and clinically relevant measures (dependent variables): metabolic stress (Z score scale), high sensitivity C-reactive protein, insulin resistance and HOMA for insulin resistance (HOMA-IR) [32] (Methods and Figure 8). By contrast, the original version AgeAccelGrim exhibited less significant associations with these biomarkers (Figure 8).

We briefly mention that age-adjusted DNAm-based surrogate markers of *saliva* PAI-1, log-scale A1C, log-scale CRP, ADM and TIMP1 show significant associations with those clinical measures as well. Analogous to what we found in analyzing CT scan data, saliva based DNAm PAI-1 and A1C are more sensitive biomarkers than AgeAccelGrim2 when it comes to metabolic stress: positive associations with DNAmPAI-1 (P=1.11x10^-14^), DNAmlogA1C (P=4.42x10^-13^) or AgeAccelGrim2 (P=1.14x10^-5^, Figure 8). Overall, this analysis shows that DNAmGrimAge2 is superior to DNAmGrimAge when it comes to studying the relationship between saliva methylation data and clinical biomarkers of metabolic stress.

### Polygenic risk score analysis

Recently, we performed a large-scale genome-wide association study (GWAS, n>40,000) on epigenetic biomarkers including AgeAccelGrim which described a polygenic risk score (PRS) for AgeAccelGrim in individuals of European ancestry [33]. Here, we repeated the PRS analysis in the WHI cohorts and showed that the PRS scores could explain 0.04% to 1.88% variation in AgeAccelGrim and 0.03% to 2.17% in AgeAccelGrim2 in postmenopausal women of European ancestry (Methods, Supplementary Figure 22). The PRS scores based on the SNPs with P<0.01 and P<0.05 tended to explain more variation in both versions of GrimAge acceleration measures.

### Epigenome-wide association study of mortality related traits

We carried out epigenome-wide association study (EWAS) for 1) AgeAccelGrim2, 2) AgeAccelGrim, 3) time-to-death and 4) time-to-CHD using our validation data. For the censored time variables (time-to-death and time-to-CHD), we evaluated three Cox regression models: model I is a basic model that adjusted for age, gender and batch effects; model II additionally adjusted for smoking pack-years, and model III additionally adjusted for blood cell composition (Methods).

The individual EWAS results for each cohort were combined via inverse variance weighted fixed effect models (Methods). A considerable number of CpGs exhibit highly significant associations with both AgeAccelGrim2 and AgeAccelGrim (Supplementary Figure 23A, 23B). The cg05575921 on chromosome (Chr) 5, near *AHRR,* shows the strongest negative correlation for both GrimAge clocks (meta P=3.6x10^-1253^ for AgeAccelGrim2 and P=1.5x10^-2023^ for AgeAccelGrim). The gene *AHRR* (Aryl Hydrocarbon Receptor Repressor) is implicated in regulation of cell growth and differentiation. The CpG cg23842572 on Chr17, near *MPRIP,* shows the strongest positive correlation with AgeAcceGrim2 (P=3.0x10^-424^) and the CpG cg13525276 on Chr14, near *TSHR,* shows the strongest positive correlation with AgeAccelGrim (P=4.7x10^-254^). *MPRIP* encodes a protein interacts with both myosin phosphatase and RhoA and *TSHR* encodes the receptor for the thyroid-stimulating hormone (TSH) or thyrotropin. These 3 genes were also identified in the EWAS of time-to-death (Supplementary Figure 23C–23E). Several studies previously showed hypomethylation of cg05575921 on *AHRR* was associated with smoking [34, 35]. Our analysis shows that cg05575921 is the best leading CpG hypomethylated associated with mortality in Model I (P=1.2x10^-69^). However, cg05575921 is still one of the top CpGs associated with mortality even after adjusting for smoking pack-years (top 10 on Model II, P= 1.6x10^-26^, Supplementary Figure 23D). Its association with mortality risk is not confounded by blood cell counts as it is the leading CpG associated with mortality in Model III (P=7.8x10^-53^, Supplementary Figure 23E). We also broadly viewed the correlation between EWAS of age acceleration from our GrimAge clocks and EWAS of time-to-death. EWAS results for time-to-death are strongly correlated with those for AgeAccelGrim2 (r=0.616 in mortality Model I and r=0.54 in Model II, Supplementary Figure 24A, 24B). The pairwise EWAS correlation is attenuated (r=0.264, Supplementary Figure 24C) when using Model III which removes the effect of blood cell composition. The EWAS results for time to CHD (n=6143) exhibit a weaker correlation with EWAS of AgeAccelGrim2 (Supplementary Figures 25, 26).

## DISCUSSION

Many studies have shown that the original version of GrimAge predicts mortality and morbidity risk (e.g. [8–17]). To arrive at version 2 of GrimAge, we developed two additional DNAm based surrogates for plasma proteins that are widely used in the clinic (DNAm logCRP and DNAm logA1C). Our comprehensive validation analysis show that GrimAge2 outperforms GrimAge with respect to its association with time-to-death, time-to-CHD, time-to-CHF, and assessing the associations with a host of age-related conditions: dysfunctions related to kidney, lung, metabolism, cognitive behavior, lipid, and vital signs, and CT-derived measures of adiposity. The reported associations remain highly significant even after adjusting for seven imputed measures of blood cell composition.

To evaluate the new version of GrimAge, our association analysis covered a broad category of age-related phenotypes including clinically relevant measures and lifestyle behaviors. These results confirm that AgeAccelGrim2 is more strongly associated with age related phenotypes than AgeAccelGrim. Further, our new estimators DNAm logCRP and DNAm logA1C, are associated with a host of age-related conditions. GrimAge2 was trained in 1833 individuals from the FHS cohort aged between 40 and 92 years old (median age at 65). Thus, it is expected to work well in older adults. We demonstrate that it can be applied to younger individuals, but it leads to a systematic offset compared to chronological age. This offset can be removed by using a suitable regression model.

For most protein markers (except for CRP and A1C), the protein measurement preceded the DNA methylation measurement by about 6.6 years. This suggests that the protein measurement (and the accompanying organ dysfunction) affected the methylation levels (as opposed to the other way around).

The first version of GrimAge (AgeAccelGrim) has been used in human clinical trials [36]. Our polygenic risk scores correlate only weakly with AgeAccelGrim2, similar to what has been observed for AgeAccelGrim [33]. Unlike genetic factors, lifestyle factors (as reflected in smoking, mean carotenoid levels, adiposity, educational level) exhibit strong correlations with AgeAccelGrim2. Lifestyle factors also relate to our DNAm based estimates of logCRP, logA1C, PAI-1, and smoking pack-years.

We also showed that GrimAge2 can be applied to saliva methylation data but leads to a noticeable offset.

GrimAge2 will not replace existing clinical biomarkers. Rather, GrimAge2 complements existing clinical biomarkers when evaluating an individual’s aging rate.

## MATERIALS AND METHODS

### Framingham Heart Study cohort for training DNAmGrimAge2

The FHS offspring cohort [19] is a large-scale longitudinal study started in 1948, initially investigating risk factors for cardiovascular disease (CVD, Supplementary Note 2). Previously, we used 2,356 individuals from the FHS in training and testing DNAmGrimAge. In establishing DNAmGrimAge2, we used the same individuals plus about 200 more individuals from the same offspring cohort. Those individuals were excluded in establishing the first GrimAge clock due to lack of protein measures [1]. To build the new mortality clock, we applied more stringent quality controls to remove technical outliers. It yielded a total of 2,544 individuals from 939 pedigrees. We assigned 2/3 pedigrees (1833 individuals/622 pedigrees) to the training data and 1/3 pedigrees (711 individuals from 317 pedigrees) to the test data (Table 1).

The FHS cohort contains medical history and measurements, immunoassays at exam 7, and blood DNA methylation profiling at exam 8. The technology of immunoassay was based on Luminex xMAP assay, an extension of the enzyme-linked immunosorbent assay (ELISA) performed with multiple analyte-specific capture antibodies bound to a set of fluorescent beads. The measurement of observed CRP, A1C and smoking pack aligned with the measurement of methylation array at exam 8 in FHS offspring cohort. But the measurement of the other seven plasma proteins (exam 7) preceded the measurement of blood DNAm data (exam 8) by 6.6 years, suggesting that the DNAm profiles may not represent a highly accurate snapshot of the status of these proteins at the time of blood collection.

The DNA methylation profiling was based on the Illumina Infinium HumanMethylation450K BeadChip.

### Two-stage approach for establishing DNAmGrimAge2

### Stage 1: develop DNAmlogCRP and DNAmlogA1C

The training dataset was used to build the two new DNAm based surrogate markers for the log scale of C-reactive protein (logCRP) and log scale of hemoglobin. Both plasma proteins were measured on exam 8. CRP levels were measured based on an immuno-turbidometric array. We scaled the CRP and A1C variables before log transformation and defined extreme values based on the raw values of the observations whose scale values were ≤6 and the closest to 6. The range of winsorized CRP is between 0.14 and 54.01 mg/L and the range of winsorized A1C level is between 4.7% and 10%. We applied log-transformation on the winsorized variables. Our previous DNAmGrimAge involves 1030 CpGs for establishing the surrogate of DNAm proteins or smoking pack-years. Each plasma protein (log CRP or log A1C) was regressed on the 1030 CpGs, chronological age (at exam 8) and sex (an indicator of female) using the elastic net regression model implemented in the R package *glmnet*. Ten-fold cross validation was performed in the FHS training data to specify the underlying tuning parameter λ.

### Stage 2: define DNAmGrimAge2

In the second stage, we added chronological age, gender, DNAmlogCRP and DNAmlogA1C, the other 10 previously defined DNAm biomarkers to build a new GrimAge—*DNAmGrimAge2*. All the 12 DNAm biomarkers are moderately correlated with their targets (protein or smoking pack-years). The correlation estimates between DNAm biomarkers and their corresponding targets have a distribution of 0.64±0.12 [0.43, 0.86] (mean±SD [range]) in the training dataset and a distribution of 0.42±0.09 [0.34, 0.66] in the test dataset (Supplementary Table 1). The correlation estimates between DNAm biomarkers and chronological ages have a broad range in both training (0.48±0.31 [0.06, 0.92]) and test dataset (0.45±0.35 [0.05, 0.90], as listed in Supplementary Table 1. Of those, DNAmLeptin shows the lowest age correlation (*r* ~0.05) and DNAmTIMP1 shows the highest age correlation *r*~0.90). Regardless of whether the protein measures (based on immune assay) or self-report smoking pack-years were available or not, we estimated the 12 DNAm surrogates for all the FHS individuals (1833 in the training and 711 in the test data).

### Definition of DNAm GrimAge

We used an elastic net Cox regression model [37] to regress time-to-death (due to all-cause mortality) since exam 7 on the 12 DNAm based surrogate markers (Supplementary Table 1.1), chronological age, and sex. The elastic net model selected all the available covariates except for DNAm CD56 and DNAm EFEMP1. As part of stage 2, we validated the accuracy of the DNAm based surrogate markers for their observed counterparts in the FHS test dataset. However, the mortality predictor (DNAmGrimAge2) was only fit in the FHS training dataset (N=1833). In the training dataset, we performed 10-fold cross validation to specify the value of the tuning parameter λ.

### Calibration of DNAm GrimAge into units of years

The final elastic net Cox model is listed in Table 3 results in an uncalibrated DNAmGrimAge2 estimate, which can be interpreted as the linear combination of the covariates, *X^T^β*, or alternatively as the logarithm of the hazard ratio, *h*(*t*)/*h*_0_(*t*) = *X^T^β*, where *h*_0_(*t*) is the baseline hazard at time *t*. The linear combination, *X^T^β*, can be interpreted as an uncalibrated version of DNAm GrimAge. To facilitate an intuitive interpretation as a physiological age estimator, we linearly transformed it so that the resulting estimate would be in units of years. Toward this end, we imposed the following requirement: the mean and variance of the resulting value of DNA GrimAge2, should be the same as the mean and variance of the age variable in the FHS training data (average of exam 7 and exam8). This resulted in the following transformation

DNAm GrimAge2 =−61.03936 + 8.271105 * *X^T^β*.

**Table 3:** Cox elastic net regression model.

**Covariate (*X*)**	**Abbreviation**	**Coefficients (β)**
DNAm adrenomedullin	DNAmADM	0.00609
DNAm beta-2-microglobulin	DNAmB2M	2.79E-07
DNAm cystatin-C	DNAmCystatin C	4.08E-06
DNAm growth differentiation factor 15	DNAmGDF-15	0.00035
DNAm leptin	DNAmLeptin	-2.03E-05
DNAm log C-reactive protein	DNAmlogCRP	1.90266
DNAm log hemoglobin A1C	DNAmlogA1C	0.40359
DNAm plasminogen activator inhibitor 1	DNAmPAI-1	0.02941
DNAm tissue inhibitor metalloproteinases 1	DNAmTIMP-1	3.67E-06
DNAm pack-years	DNAmPACKYRS	0.00014
Chronological age	Age	0.02676
Female	Female	-0.14212

The table lists the finalized covariates in the final Cox regression model with elastic net penalty and their coeffects. A linear combination of *X^T^*β yields an estimate of logarithm of proportional hazard, which is the raw value of DNAmGrimAge2 before calibration. The finalized DNAm GrimAge2 is based on transforming the raw variable into a distribution in units of year The columns report the name of the covariate (e.g. DNAm based biomarker), its abbreviation and coefficient under the final Cox regression model with tuning parameter λ determined by 10-fold cross validation.

A completely unbiased evaluation of DNAm GrimAge2 is achieved in eight large-scale cohorts independent from the FHS test, as described below.

### Software

GrimAge2 approach is implemented in our online software, https://dnamage.clockfoundation.org/.

### Mortality risk: *mortality.res*

Formally, mortality.res is defined as the deviance residual from a Cox regression model for time-to-death due to all-cause mortality. The variable mortality.res can be interpreted as a measure of “excess” mortality risk compared to the baseline risk in a test data.

### Validation data

We validated DNAmGrimAge2, DNAmGrimAge and their components in 13,399 blood samples from 10,065 individuals across 1) FHS test and the other eight cohorts: 2) BA23 and 3) EMPC study from the Women’s Health Initiative (WHI) with three racial groups, 4) African Americans from the Jackson Heart Study (JHS), 5) the InCHIANTI cohort study, 6) individuals of European ancestry from Baltimore Longitudinal Study of Aging (BLSA), 7) Lothian Birth Cohort 1921 (LBC1921) and 8) LBC 1936 (LBC1936), and 9) individuals of European ancestry from Normative Aging Study (NAS, only recruiting male participants). Table 1 lists the characteristics of the samples. Descriptions of each study cohort including characteristics of participants, phenotype data and molecular array samples can be found in Supplementary Note 2. Methylation arrays were profiled in Illumina 450k for all cohorts except for the JHS which used the EPIC array. Methylation beta values were generated using the Bioconductor *minfi* package with Noob background correction [38] for all the validation data except WHI, INS and NAS, which were based on other algorithms such as BMIQ [39] or SeSAMe [40] (Supplementary Note 2).

### Multivariate regression analysis for validation

We validated our new mortality clock DNAm GrimAge2 in two parts. In the first part, we focused on validating the new clock using multivariate regression analysis that adjusted for potential confounders including sex. Here we only analyzed the associations with age-related phenotypes such as mortality. In the second part, we validate the new clock in a broader category of variables including diet and other lifestyle factors that are not necessarily related to chronological age. Here, we addressed different effect sizes between males and females along with sex-stratified analyses. The details for the second part are described in the next section, In the first part, our validation analysis involved i) Cox regression for time to death, for time-to-CHD, for time to CHF, time-to-any cancer ii) linear regression for our DNAm based measures (independent variable) associated with and number of age-related conditions (dependent variable), respectively, iii) linear regression for age at menopause (independent variable) associated with our DNAm measure (dependent variable), with only one exception for the relationship with DNAm PACKYRS (as an independent variable), iv) logistic regression analysis for estimating the odds ratios of our DNAm based measure associated with hypertension, type 2 diabetes, and disease free status. The variable of “number of age-related conditions” includes arthritis, cataract, cancer, CHD, CHF, emphysema, glaucoma, lipid condition, osteoporosis, type 2 diabetes, etc. (see Supplementary Note 1). In our validation analysis, we used AgeAccelGrim2, AgeAccelGrim, and used the scaled measures of seven DNAm surrogates for plasma proteins based on the mean and standard deviation (SD) of the FHS training dataset such that the effect size was approximately corresponding to one SD. All the models were adjusted for age, female, and adjusted for batch effect as needed. To avoid the bias due to familial correlations from pedigrees in the FHS cohort or the intra-subject correlations from the repeated measures in InCHIANTI, LBC1921, LBC1936 and NAS, we accounted for the correlations accordingly in all the analyses in the following. In Cox regression analysis, we used robust standard errors, the Huber sandwich estimator, implemented in R *coxph* function. We used linear mixed models with a random intercept term, implemented in *lme* R function. We used generalized estimation equation models (GEE), implemented in R *gee* function, for our logistic regression models. Analysis was performed across different strata formed by racial groups at each study cohort, with up to 15 strata for the meta analyses (Table 1). For the meta analyses, we used fixed effect models weighted by inverse variance to combine the results across validation study sets into a single estimate by using the *metafor* R function in most situations. We also used Stouffer’s meta-analysis method (weighted by the square root of the sample size) in specific situations where the harmonization of covariates across cohorts was challenging, e.g. when evaluating the number of age-related conditions and disease free status.

### Diet, clinical biomarkers and lifestyle factors

We performed a robust correlation analysis (biweight midcorrelation, bicor [26]) between our novel biomarkers (AgeAccelGrim2, AgeAccelGrim and its 10 age-adjusted components) and a total of 61 variables including 27 self-reported diet, 9 dietary biomarkers, 19 clinically relevant measurements, and 6 lifestyle factors including hand grip strength. The sample size for each variable is up to 13,420 across the nine validation datasets including the FHS test dataset. We combined the postmenopausal women from the WHI BA23 and WHI EMPC (roughly n= 4000 women). The 9 dietary biomarkers are only available in the WHI cohort. Blood biomarkers were measured from fasting plasma collected at baseline. Food groups and nutrients are inclusive, including all types and all preparation methods, e.g. folic acid includes synthetic and natural, dairy includes cheese and all types of milk. The individual variables of WHI are explained in [25].

The study variables are listed in Supplementary Table 2.1. We also included the individuals with African American (AfricanA) ancestry (n up to 216) from the BLSA cohort, who were excluded from mortality analysis due to the very low death rate (8%). For each study cohort, we stratified the samples based on ethnic_gender category. For instance, the BLSA samples were stratified to 4 strata: White_male, White_female, AfricanA_male, and AfricanA_female. The WHI samples were stratified by European-, African-, and Hispanic- ancestry groups. Ancestry information was verified using ancestry informative SNP markers. We conducted robust correlation (bicor) analysis stratified by study cohort/ethnicity/sex and meta-analyzed the results with fixed effect models weighted by inverse variance. The fixed effect models yield a meta estimate of bicor. As a caveat, the bicor analysis did not accommodate the intra-pedigree (e.g. FHS) or intra-subject correlation (e.g. LBC1921). We did not employ statistical analyses such as linear mixed models to accommodate these factors since some models failed to reach convergence due to the unbalanced design in the data structure or high intra-subject correlations. The patterns for the failures of convergence were heterogeneous in terms of study cohort or study variables (dependent or independent variables). Our robust correlation (bicor) results in individual strata were meta-analyzed across strata resulting in meta estimates of bicor and its P-value, which could be inflated by intra pedigree/subject correlations. The harmonization of educational level across cohorts was challenging since some cohorts report years of education while others simply report categorical variables for education status. Here correlation coefficients can be attractive since they are invariant with respect to linear transformations.

### Polygenic risk score analysis

We performed polygenic risk score (PRS) analysis in women of European ancestry from the WHI BA23 and AS315, using the GWAS results of AgeAccelGrim from our previous study [33]. The PRS analysis was restricted to the women of European ancestry since the GWAS results were based on the European ancestry meta-analyses on 34,710 individuals. The PRS scores were generated using default settings of the PRSice software [41] (clump-window = 250 kb, clump-*p* = 1; clump-r2 = 0.25). P-value thresholds for SNP associations were set at < 5 × 10^−8^, < 0.01, < 0.05, < 0.1, < 0.5, and 1. The linkage disequilibrium (LD) estimation was calculated using the target data (WHI). The qualities of genotyped and imputed SNPs in the WHI cohort were controlled by empirical MAF >=0.005, Hardy-Weinberg equilibrium (HWE) P-value >=1.0e-06 and MaCH impute r2≥0.6 [42]. Genotyped and imputed SNP array information are described in the Supplementary Note 2. We performed linear regression analyses of AgeAccelGrim2 (or AgeAccelGrim) on PRS to compute the proportions for the variation of the age acceleration measure explained by PRS at different thresholds. We report the proportion of R^2^ in percentages (%).

### Computed tomography data from the Framingham Heart Study

The computed tomography (CT) in liver, spleen, paraspinal muscle, subcutaneous adipose tissue (SAT), and visceral adipose tissue (VAT) were performed in n=2,803 individuals from the FHS Offspring, Third Generation and Omni 2 Cohort participants between September 2008 and December 2011 [27, 28]. Of those, 1,174 Offspring Cohort participants were included in our FHS study (869 in training and 305 in test data). The age at CT scan was in general slightly older than the age at blood draw for the DNA methylation profile (mean age difference= 3.7 years, ranging from 1.2 to 6.1 years).

Organ density measures, more precisely CT attenuation coefficients, reflect how easily a target can be penetrated by an X-ray. The Hounsfield unit (HU) scale is a linear transformation of the original linear attenuation coefficient measurement into one in which the radiodensity of distilled water is defined as zero Hounsfield units (HU). Radiation attenuation in liver, spleen, or muscle is inversely related to respective measures of fat content.

The CT measures from three areas of the liver, two areas of the spleen and two areas of the paraspinal muscle were averaged to determine the average Hounsfield units in liver, spleen and muscle, respectively. The CT-scan measures of visceral and subcutaneous adipose tissue are described in [28].

In our analysis, we first performed marginal robust correlation analysis (biweight midcorrelation, bicor coefficient) [26] to study the association between the CT-scan derived measures and DNAm based biomarkers. As sex affects adipose associated parameters, we performed the analysis in males and females, separately. Next we combined the results using fixed effects meta analysis. To adjust for potential confounders, we also performed four types of multivariate linear mixed effects models that included sex and BMI as fixed effects and pedigree structure as a random effect. In Model I, we regressed a DNAm based biomarker (e.g. AgeAccelGrim2) on CT derived covariates: liver density, spleen density, and paraspinal muscle density. In Model II, we regressed the DNAm based biomarker (dependent variable) on volumetric measures of adipose tissue (both SAT and VAT volume). In Model III, we regressed the DNAm based biomarker (dependent variable) on both volumetric (in units of cm3) and density (in units of HU) measures of adipose tissue (both SAT and VAT). This model allows us to assess which measure is more sensible for our DNAm biomarkers. In Model IV, we used all CT measures as covariates (i.e. liver, spleen and muscle density, SAT volume, and VAT volume). We did not include the density measures of SAT or VAT as Model III showed that they were not significant after adjusting for SAT/VAT volumes in most of our analysis. Also, it can protect the model fit in Model IV from the issue of multi-collinearity. We used the BMI measure assessed at exam 9 in the FHS, i.e. the closest exam following the CT-scan exam. We used all the FHS individuals from training and test dataset as our previous study showed the results were not biased by the training status [1].

### Application in saliva samples in National Growth and Health Study (NGHS) cohort

We applied our mortality clocks in 432 mothers from the NHLBI Growth and Health Study (NGHS) cohort [31]. The NGHS cohort was a longitudinal study conducted from 1985 to 2000 that investigated the racial differences in factors relating to the development of obesity in Black and White pre-adolescent girls, who were recruited at age 9 or 10 years. A 30-year follow-up of the Contra Costa County cohort was conducted in 2016 [31] to assess midlife health and well-being. Methylation data from the Illumina 850k array were profiled in saliva samples from 688 individuals including mothers (n=442) and their most recent children (n=246). We only used mothers in our analysis. Of the 442 mothers, 10 women had either missing ethnic status, low confidence in the estimate of chronological age, or were technical outliers and removed from analysis, yielding 432 mothers for our study. The mothers in our study are balanced by ethnic/racial groups: White (n=218) and African American (n=214). More details of the NGHS cohort are described in Supplementary Note 2.

We performed multivariate linear regression analysis to study the association between 1) dependent variables: clinically relevant measures: metabolic Z score, high sensitivity C-reactive protein (CRP), insulin resistant and HOMA for insulin resistance (HOMA-IR) [32] and 2) independent variables: AgeAccelGrim2, AgeAccelGrim, and nine scaled DNAm-based surrogates of proteins and DNAm PACKYS. The HOMA-IR stands for homeostatic model assessment of insulin resistance defined by Matthews et al. [32, 43]. The equations for HOMA1-IR = (FPI × FPG)/22.5, where FPI is fasting plasma insulin concentration (mU/l) and FPG is fasting plasma glucose (mmol/l) [43]. Higher scores of HOMW-IR represent greater levels of insulin resistance. We applied the analysis in all mothers and stratified analysis by ethnic/racial group, respectively. All the analysis was adjusted for chronological age, batch effect and for race as needed.

### Meta analysis for EWAS of age acceleration of GrimAge clocks

We performed EWAS of AgeAccelGrim2 (and AgeAccelGrim) in each cohort stratified by gender and race. EWAS of epigenetic age acceleration was carried out with the R function *standardScreeningNumericTrait* from the R WGCNA package. AgeAccelGrim2 (AgeAccelGrim) was based on the residuals adjusted for pedigree correlation or intra-subject correlation via linear mixed analysis in the FHS, InChinanti, LBC21, LBC36 and NAS cohorts. EWAS results were combined via fixed effect models weighted by inverse variance with effect sizes based on correlation estimates, as implicated in R metafor.

### Meta analysis for EWAS of time-to-death and time-to-CHD

We performed EWAS of time-to-death on each cohort based on three Cox regression models of models. Model I is a basicmodel that adjusted for chronological age and sex (Female: 1 indicates females, 0 males), and batch effect, pedigree correlation or intrasubject correlation as needed. Model II adjusted for the same variables as in Model I plus smoking history based on pack-years. Model III adjusted for the same variables as in Model I plus 7 imputed blood cell compositions/counts: CD8 naïve, CD8pCD28nCD45Ran (exhausted cytotoxic T cells), plasma blasts, CD4+ T, nature killer cells, monocytes and granulocytes (Houseman estimates, Horvath estimates). Robust standard errors (the Huber sandwich estimator) was used if the Cox regression analysis involved pedigree correlation or intrasubject correlation. As information on smoking pack-years was missing in JHS, BLSA and LBC21, we used smoking status (never, past and current) in the Model II. EWAS results were combined via fixed effect models weighted by inverse variance with effect sizes based on beta values (log hazard ratios), from the Cox regression models, as implicated in R metafor.

For all the individual EWAS, we restricted the analysis to CpGs present on 450k array. For each CpG, individuals with extreme methylation levels (six standard deviations away from the mean) were set to missing. EWAS of AgeAccelGrim2/AgeAccelGrim using the FHS cohort was only performed on the 711 individuals from the test set. The meta analysis for AgeAccelGrim2/AgeAccelGrim was performed on n=12,430. The meta analysis was performed on n=13,260 for time-to-death and n=6,143 for time-to-CHD based on FHS, WHI BA23 and JHS cohorts.

## Supplementary Material

Supplementary Notes

Supplementary Figures

Supplementary Tables 1.1-2.1

Supplementary Table 2.2

Supplementary Table 2.3

Supplementary Table 2.4

Supplementary Table 2.5

Supplementary Table 2.6

Supplementary Table 2.7

Supplementary Table 2.8

Supplementary Table 2.9

Supplementary Table 2.10

Supplementary Table 2.11

Supplementary Table 2.12

Supplementary Table 2.13

Supplementary Tables 3.1-3.5

Supplementary Table 4.1

Supplementary Table 4.2

Supplementary Table 4.3
