# Supplementary material for: DNA methylation GrimAge version 2
Source: Aging (Albany NY). 2022 Dec 14;14(23):9484–549. doi: 10.18632/aging.204434 (PMC9792204; doi:10.18632/aging.204434)
Supplement: Supplementary Notes [file aging-14-204434-s001.pdf]

SUPPLEMENTARY MATERIALS

Supplementary Note 1: DNAm based surrogates for plasma proteins

The model of DNAm GrimAge2 is composed of nine DNAm based plasma proteins, DNAm based pack years, age and gender. Below we briefly describe these nine plasma proteins.

A1C (hemoglobin A1C, HbA1c; glycosylated hemoglobin; Glycated hemoglobin) is a blood test that shows average blood sugar (glucose) levels over the last 3 months. This biomarker is widely used in clinic to check for prediabetes or diabetes and help guide diabetes treatment over time (http://uclahealthib.staywellsolutionsonline.com/Bedside/167,a1c). Previous studies also indicated that higher levels of A1C were associated with cardiovascular heart disease and mortality [1, 2]. The log scale of A1C is a new component in DNAm GrimAge2.

ADM (adrenomedullin) is a vasodilator peptide hormone. Plasma ADM, initially isolated from adrenal gland, is increased in individuals with hypertension and heart failure [3]. A recent study showed that ADM was involved in age-related memory loss in mice and aging human brains [4].

B2M (Beta-2 microglobulin) is a component of major histocompatibility complex class 1 (MHC I) molecular. Plasma B2M is a clinical biomarker associated with cardiovascular disease, kidney function, inflammation severity [5]. B2M is a pro-aging factor associated with cognitive and regenerative function in aging process and suggests B2M may be targeted therapeutically in old age [6]. A previous study showed that systemic B2M accumulation in aging blood promoted age-related cognitive dysfunction and impairs mouse models [6].

Cystatin C or cystatin 3 (formerly gamma trace, post-gamma-globulin, or neuroendocrine basic polypeptide) is mainly used as a biomarker of kidney function. Plasma cystatin-C is a clinical relevant biomarker indicating kidney function [7]. Cystatin-C seems plays a role in cardiovascular disease [8] or amyloid deposition associated with Alzheimer's disease [9].

C-reactive protein (CRP) test is clinically used to find inflammation in your body that could be caused by different types of conditions such as an infection or autoimmune disorders like rheumatoid arthritis or inflammatory bowel disease, (https://uclahealthib.staywellsolutionsonline.com/Search/167,c_reactive_protein_serum). Several previous studies indicated that CPR protein concentration is associated with coronary heart disease, stroke, and non-vascular mortality (e.g. [10, 11]). The log scale of CRP is a new component in DNAm GrimAge2.

GDF-15 (growth differentiation factor 15) is one of transforming growth factor beta subfamily. GDF-15 has been implicated in aging and age- related disorders. It also plays a role in age-related mitochondria dysfunction [12].

Leptin is a hormone predominantly in adipose cells. Leptin plays a role in regulating energy balance by inhibiting hunger and is implicated in Alzheimer's disease [13].

Plasminogen activator inhibitor antigen type 1(PAI-1) is the major inhibitor of tissue-type plasminogen activator and unokinase plasminogen activator. PAI-1, released in response to inflammation process, plays a central role in a number of age-related subclinical (i.e., inflammation, atherosclerosis, insulin resistance) and clinical conditions (i.e., obesity, comorbidities) [14].

TIMP-1 or TIMP metallopeptidase inhibitor 1 is a tissue inhibitor of metalloproteinases. It is also involves chromatin structures, promoting cell proliferation in a wide range of cell types, and may also have an anti-apoptotic function [15].

Supplementary Note 2: Description of datasets

Our study participants with blood samples came from nine independent cohorts: Framingham Heart Study Offspring Cohort (FHS), Women's Health Initiatives (WHI) BA23, WHI EMPC, Jackson Heart Study (JHS), InCHIANTI (baseline and the third follow-up), Baltimore Longitudinal Study of Aging (BLSA), Lothian Birth Cohort 1921 (LBC21) and LBC 1936 (LBC36), and Normative Aging Study (NAS). We also studied saliva samples collected from an independent study: the NHLBI Growth and Health Study (NGHS) cohort. Below we describe each study cohort/datasets in more details.

Study 1: Framingham Heart Study cohort

The FHS cohort [16] is a large-scale longitudinal study started in 1948, initially investigating the common factors of characteristics that contribute to cardiovascular disease (CVD), https://www.framinghamheartstudy.org/index.php. The study initially enrolled participants living in the town of Framingham, Massachusetts, who were free of overt symptoms of CVD, heart attack or stroke at enrollment. In 1971, the study started the FHS

Offspring Cohort to enroll a second generation of the original participants 'adult children and their spouses (n= 5124) to conduct similar examinations [17]. Participants from the FHS Offspring Cohort were eligible for our study if they attended both the seventh and eighth examination cycles and consented to having their molecular data used for the study. We used the 2,544 participants from the group of Health/Medical/Biomedical (IRB, MDS) consent with available DNA methylation array data. The FHS data are available in dbGaP (accession number: phs000363.v16.p10 and phs000724.v2.p9).

We computed the total number of age-related conditions based on dyslipidemia, hypertension, cardiovascular disease (including coronary heart disease [CHD] or congestive heart failure [CHF]), type 2 diabetes, cancer and arthritis. Time to CHD or time to CHF was truncated at zero if it occurred before exam 8. Deaths among the FHS participants that occurred prior to January 1, 2013 were ascertained using multiple strategies, including routine contact with participants for health history updates, surveillance at the local hospital and in obituaries of the local newspaper, and queries to the National Death Index. Death certificates, hospital and nursing home records prior to death, and autopsy reports were requested. When cause of death was undeterminable, the next of kin were interviewed. The date and cause of death were reviewed by an endpoint panel of 3 investigators.

DNA methylation quantification

Peripheral blood samples were collected at the 8th examination. Genomic DNA was extracted from buffy coat using the Gentra Puregene DNA extraction kit (Qiagen) and bisulfite converted using the EZ DNA Methylation kit (Zymo Research Corporation). DNA methylation quantification was conducted in two laboratory batches using the Illumina Infinium HumanMethylation450 array (Illumina). Methylation beta values were generated using the Bioconductor *minfi* package with Noob background correction [18].

Studies 2 and 3 :Women's Health Initiative

The WHI is a national study that enrolled postmenopausal women aged 50-79 years into the clinical trials (CT) or observational study (OS) cohorts between 1993 and 1998 [19, 20]. We included 4,079 WHI participants with available phenotype and DNA methylation array data: 2,107 women from "*Broad Agency Award 23*" (WHI BA23) and 1,972 women from "*Epigenetic Mechanisms of PM-Mediated CVD Risk*" (WHI EMPC). WHI BA23 focuses on identifying miRNA and genomic biomarkers of coronary heart disease (CHD), integrating the biomarkers into diagnostic and prognostic predictors of CHD and other related phenotypes, and other objectives can be found in https://www.whi.org/researchers/data/WHIStudies/StudySites/BA23/Pages/home.aspx. WHI EMPC is a study of epigenetic mechanisms underlying associations between ambient particulate matter (PM) air pollution and cardiovascular disease [21]. WHI EMPC and BA23 span three WHI sub-cohorts including GARNET, WHIMS and SHARe. 936 EMPC participants were not in any of the WHI GWAS (either GARNET, WHIMS, SHARe, MOPMAP, HIPFX, or GECCO). The largest overlap was with SHARE and GARNET. There was almost no overlap with WHIMS and MOPMAP. The death status was based on the variable DEATHALL (All Discovered Death) as listed in the form "All Discovered Death Outcome Detail (Form 124/120)". This variable does not censor deaths that occur after the participants 'last consent period. The original WHI study began in the early 1990s and concluded in 2005. Since 2005, the WHI has continued as Extension Studies (Ext1), which are annual collections of health updates and outcomes in active participants. The second Extension Study (Ext2) enrolled 93,500 women in 2010 and follow-up of these women continues annually. Death was adjudicated for clinical trial (CT) and observational study (OS) participants through Ext1. In Ext2, death is only adjudicated for the Medical Record Cohort (MRC). Non MRC cause of death is determined by the initial cause of death form (form 120).

The total number of age-related conditions was based on Alzheimer's disease, amyotrophic lateral sclerosis, arthritis, cancer, cataract, CVD, glaucoma, emphysema, hypertension, and osteoporosis.

DNA methylation quantification for BA23

In brief, bisulfite conversion using the Zymo EZ DNA Methylation Kit (Zymo Research, Orange, CA, USA) as well as subsequent hybridization of the HumanMethylation450k Bead Chip (Illumina, San Diego, CA), and scanning (iScan, Illumina) were performed according to the manufacturers protocols by applying standard settings. DNA methylation levels (β values) were determined by calculating the ratio of intensities between methylated (signal A) and un-methylated (signal B) sites. Specifically, the β value was calculated from the intensity of the methylated (M corresponding to signal A) and un-methylated (U corresponding to signal B) sites, as the ratio of fluorescent signals $\beta = \text{Max}(M,0)/[\text{Max}(M,0)+\text{Max}(U,0)+100]$. Thus, β values range from 0 (completely un-methylated) to 1 (completely methylated).

DNA methylation quantification for WHI EMPC

Illumina Infinium HumanMethylation450 BeadChip data from the Northwestern University Genomics Core

Facility for WHI EMPC participants sampled in stages 1a, 1b, and 2 were quality controlled, normalized and batch adjusted. Beta-mixture quantile normalization was implemented using BMIQ [22] and empirical Bayes methods of batch adjustment for stage and plate were implemented in ComBat [23].

SNP array data

WHI SNP array data were generated under different sub-study groups: GARNET, SHARe and WHIM. The genotyped SNPs were profiled in different platforms. The information is presented in the format of platform (dbGAP access number): Illumina HumanOmni1-Quad v1-0 B (phs000200.v10.p3), Illumina HumanOmniExpressExome-8v1_B (phs000200.v10.p3), Affymetrix 6.0 (phs000200.v10.p3) and Affymetrix 6.0 (phs000200.v10.p3). More details can be found in our earlier GWAS study [24].

Lifestyle factors and dietary assessment in the Women's Health Initiative (WHI)

WHI participants completed self-administered questionnaires at baseline which provided personal information on a wide range of topics, including sociodemographic information (age, education, race, income), and current health behaviors (recreational physical activity, tobacco and alcohol exposure, and diet). Participants also visited clinics at baseline where certified Clinical Center staff collected blood specimens and measured anthropometrics (weight, height, hip and waist circumferences) and blood pressures (systolic, diastolic). Body mass index and waist to hip ratio were calculated from these measurements.

Dietary intake was assessed at baseline using the WHI Food Frequency Questionnaire [25]. Briefly, participants were asked to report on dietary habits in the past three months, including intake, frequency, and portion sizes of foods or food groups, along with questions concerning topics such as food preparation practices and types of added fats. Nutrient intake levels were then estimated from these responses. For current drinker, we use the threshold of more than one serving equivalent (14g) within the last 28 days.

Study 4: Jackson Heart Study

The JHS is a large, population-based observational study evaluating the etiology of cardiovascular, renal, and respiratory diseases among African Americans residing in the three counties (Hinds, Madison, and Rankin) that make up the Jackson, Mississippi metropolitan area [26] The age at enrollment for the unrelated cohort was 35-84 years; the family cohort included related individuals >21 years old. Participants provided extensive medical and social history, had an array of physical and biochemical measurements and diagnostic procedures, and provided genomic DNA during a baseline examination (2000-2004) and two follow-up examinations (2005-2008 and 2009-2012). Annual follow-up interviews and cohort surveillance are ongoing. In our analysis, we used the visits at baseline from 1747 individuals as part of project JHS ancillary study ASN0104, available with both phenotype and DNA methylation array data. Total numbers of age-related conditions were based on hypertension, type 2 diabetes, kidney dysfunction based on ever dialysis, and CVD.

DNA methylation quantification

Peripheral blood samples were collected at the baseline. Methylation beta values were generated using the Bioconductor *minfi* package with Noob background correction [18].

Study 5: Invecchiare in Chianti, aging in the Chianti area (InCHIANTI)

The InCHIANTI (Invecchiare in Chianti, aging in the Chianti area) cohort is a representative population-based study of older persons enrolling individuals aged 20 years and older from two areas in the Chianti region of Tuscany, Italy, http://inchiantistudy.net/wp/. One major goal of the study is to translate epidemiological research into geriatric clinical tools, ultimately advancing clinical applications in older persons. Of the cohort, 1430 observations from 728 individuals with both phenotype information and DNA methylation data were including in our studies. The observations were collected from baseline in 1998 and the third follow-up visit in 2007. All participants provided written informed consent to participate in this study. The study complied with the Declaration of Helsinki. The Italian National Institute of Research and Care on Aging Institutional Review Board approved the study protocol. We computed the total number of age-related conditions based on cancer, hypertension, myocardial infarction, Parkinson's disease, stroke and type 2 diabetes.

DNA methylation quantification

Genomic DNA was extracted from buffy coat samples using an AutoGen Flex and quantified on a Nanodrop1000 spectrophotometer prior to bisulfite conversion. Genomic DNA was bisulfite converted using Zymo EZ-96 DNA Methylation Kit (Zymo Research Corp., Irvine, CA) as per the manufacturer's protocol. CpG methylation status of 485,577 CpG sites was determined using the Illumina Infinium HumanMethylation450 BeadChip (Illumina Inc., San Diego, CA) as per the manufacturer's protocol and as previously described [27]. Initial data analysis was performed using GenomeStudio 2011.1 (Model M

Version 1.9.0, Illumina Inc.). Threshold call rate for inclusion of samples was 95%. Quality control of sample handling included comparison of clinically reported sex versus sex of the same samples determined by analysis of methylation levels of CpG sites on the X chromosome [27]. Methylation beta values were generated using SeSAMe [28].

Study 6: Baltimore Longitudinal Study of Aging (BLSA)

Established in 1958 The Baltimore Longitudinal Study of Aging (BLSA) is the longest-running scientific study of human aging in the United States [29], https://www.blsa.nih.gov/. The study population is a continuously enrolled cohort of community dwelling adults aged 20 or older who meet rigorous screening criteria. BLSA Participants return at age dependent intervals for study visits that include comprehensive clinical testing as well as evaluations of physical and cognitive function [30]. In the BLSA, blood samples were collected for DNA extraction. The mortality analysis was restricted to participants who self-identify as White (n=572). The downstream analysis including lifestyle factors was also performed among participants who self-identify as Black or African American (n=216). We computed the total number of age-related conditions based on the number of chronic diseases as defined in Fabbri et al. [31]. The BLSA data can be applied from https://www.blsa.nih.gov/.

DNA methylation quantification

DNA was quantified using Quant-iT Picogreen Reagent (Invitrogen, Grand Island, NY, USA) according to the manufacturer's instructions. 1 ug of DNA was bisulfite treated using the EZ-96 DNA methylation kit (Zymo Research, Irvine, CA, USA) according to the manufacturer's specifications for the 450k array. Converted genomic DNA was eluted in 22 μl of elution buffer. DNA methylation level was measured using Illumina Infinium HD Methylation Assay (Illumina) according to the manufacturer's instructions. Background subtraction was applied using the *preprocessIllumina* command in the *minfi* Bioconductor package [18].

There are a total 501 participants available for both DNA methylation and SNP array data remained in analysis.

Studies 7 and 8: Lothian Birth Cohorts (LBC) of 1921 and 1936

The Lothian Birth Cohorts (LBC) [32] of 1921 and 1936 are longitudinal studies of distribution and causes of cognitive functioning changes across most of the human life course, http://www.lothianbirthcohort.ed.ac.uk/. The participants of LBC1921 (born in 1921) took part in the Scottish Mental Survey (SMS) of 1932 while the participants of LBC1936 (born in 1936) took part in the SMS in 1947. Both surveys were associated with general intelligence tests for children at age 11 years and were carried out by the Scottish Council for Research in Education. The LBC1921 (n=550) began in 1999 and examined 5 waves at mean ages 79, 83, 87. 90 and 92 years while the LBC1936 (n=1091) began in 2004 and examined 5 waves at mean ages 70,73, 76, 79 and 82 years [32, 33].

We obtained DNA methylation data used in the earlier study for predicting all-cause mortality [34] in which SNP array data were also available for the study individuals. The LBC1921 is composed of 469 individuals across waves 1 and 3 individuals (n_{deaths}=451) and the LBC1936 is composed of 1044 individuals (n_{deaths}=378) across waves 1,2,3, and 4. All participants were of White Scottish ancestry. Following informed consent, venesected whole blood was collected for DNA extraction in both LBC1921 and LBC1936. Ethics permission for the LBC1921 was obtained from the Lothian Research Ethics Committee (Wave 1: LREC/1998/4/183). Ethics permission for the LBC1936 was obtained from the Multi-Centre Research Ethics Committee for Scotland (Wave 1: MREC/01/0/56), the Lothian Research Ethics Committee (Wave 1: LREC/2003/2/29). Written informed consent was obtained from all individuals. LBC methylation data have been submitted to the European Genome-phenome Archive under accession number EGAS00001000910.

DNA methylation quantification

As described in [34], DNA was extracted from 514 whole blood samples in LBC1921 and from 1,004 samples in LBC1936. Raw intensity data were backgroundcorrected and methylation beta-values generated using the R minfi package [18]. Quality control analysis was performed to remove probes with a low (<95%) detection rate at P <0.01. Manual inspection of the array control probe signals was used to identify and remove low quality samples. The Illumina-recommended threshold was used to eliminate samples with a low call rate (samples with <450,000 probes detected at P <0.01). As SNP genotyping was previously performed on LBC samples, genotypes derived from the 65 SNP control probes on the methylation array using the wateRmelon package [35] were compared to those obtained from the genotyping array to ensure sample integrity. Samples with a low match of genotypes with SNP control probes, which could indicate sample contamination or mix-up, were excluded (n = 9). Moreover, eight subjects whose predicted sex, based on XY probes, did not match reported sex were also excluded.

Study 9: Normative Aging Study

The Normative Aging Study (NAS) is a closed and ongoing cohort established in 1963 by the U.S. Veterans Administration in the Greater Boston Area [36]. The participants were aged 21–82 years and were free of any known chronic diseases at enrollment. They have undergone health examinations in a clinical center, including blood collection, every 3–5 years. We only analyzed participants who self-identify as White (98% of our samples). DNA methylation arrays were profiled in 1455 blood samples across 751 participants from first to 4th visit. Of the blood samples, 82 were entirely removed from our study based on our quality control for missingness in CpG sites (number of sites > 5000), yielding 732 participants (1373 blood samples) remained in our study. All study participants provided written informed consent before enrollment and sample collection. This study was approved by the Harvard T.H. Chan School of Public Health and the Institutional Review Boards of the Department of Veterans Affairs.

DNA methylation quantification

DNA samples were extracted using the IQAamp DNA Blood Kit (Qiagen, CA, U.S.) from the buffy coat of the whole blood collected between 1999 and 2013. We measured DNAm by the Illumina Infinium Human Methylation450K BeadChip (450 K; Illumina Inc., San Diego, CA, U.S.), which provides information on ~ 485,000 CpG sites. To minimize batch effects, we randomized the samples across 450 K BeadChip and 96-well plates based on a two-stage age-stratified algorithm so that age distributed similarly across plates [37]. Quality control analysis was quided by detection P values. More details for quality control can be found in the study from Wang et al. [38].

Saliva study: NHLBI Growth Health Study Cohort

The NHLBI Growth and Health Study (NGHS) cohort [39] was a longitudinal study conducted from 1985 to 2000 that investigated the racial differences in factors relating to the development of obesity in Black and White pre-adolescent girls. The study initially recruited girls 9 and 10 years of age from Richmond (CA, USA), Cincinnati (OH, USA), and Washington (D.C., USA). The NGHS Contra Costa County cohort (n = 887) was recruited in 1987-1988 from public and parochial schools in the Richmond Unified School District area. The area was chosen due to census data that showed approximately equal percentages of Black and White children with the smallest degree of income and occupational disparity between races. A 30-year follow-up of the Contra Costa County cohort was conducted in 2016 [39], enrolling eligible Black (n = 307) and White (n = 317) women from the original cohort approximately at 39 to 42 years of age to assess midlife health and well-being. Eligibility criteria included not being pregnant at the time of recruitment, not experiencing a pregnancy, miscarriage, or abortion in the three months prior to recruitment, and not living abroad, being incarcerated, or being otherwise institutionalized. Consenting participants participated in a baseline survey as well as biospecimen collection, which included saliva collection.

DNA methylation quantification

Methylation arrays were profiled in saliva samples from 688 individuals including mothers (n=442) and their most recent children (n=246). The saliva samples were analyzed at the Semel Institute UCLA Neurosciences Genomics Core (UNGC) using the Illumina 850k BeadChip. Genomic DNA was isolated using temperature denaturation and subjected to bisulfite conversion, PCR amplification, and DNA sequencing (EZ DNA Methylation-Gold Kit, Zymo Research). Of the 442 mothers, 10 women missing for ethnic status, with low confidence in the estimate of chronological age, or technical outliers were removed from our analysis, yielding 432 mothers (218 White and 214 Black) remained in our study.

Supplementary Methods: Estimation of blood cell counts based on DNAm levels

We estimated blood cell counts using two different software tools. First, Houseman's estimation method [40] was used to estimate the proportions of CD8+ T cells, CD4+ T, natural killer, B cells, and granulocytes (mainly neutrophils). Second, the Horvath blood cell estimation method, implemented in the advanced analysis option of the epigenetic clock software [41, 42], was used to estimate the percentage of exhausted CD8+ T cells (defined as CD28-CD45RA-), the number (count) of naïve CD8+ T cells (defined as CD45RA+CCR7+) and plasma blasts cells. We and others have shown that the estimated blood cell counts have moderately high correlations with corresponding flow cytometric measures [40, 43].

Supplementary Figures

In the figures, we use abbreviations for the names of our study cohorts as the following: FHS train and test datasets, Women's Health Initiatives (WHI) BA23, WHI EMPC, Jackson Heart Study (JHS), InCHIANTI (baseline and the third follow-up), Baltimore Longitudinal Study of Aging (BLSA), Lothian Birth Cohort 1921 (LBC21) and LBC 1936 (LBC36), and Normative Aging Study (NAS). The three racial/ethnic groups (notations) in our study cohorts are Caucasian

(White), African American (AfricanA) and Hispanic (Hispanic).

Supplementary References

1. Gerstein HC, Swedberg K, Carlsson J, McMurray JJ, Michelson EL, Olofsson B, Pfeffer MA, Yusuf S, and CHARM Program Investigators. The hemoglobin A1c level as a progressive risk factor for cardiovascular death, hospitalization for heart failure, or death in patients with chronic heart failure: an analysis of the Candesartan in Heart failure: Assessment of Reduction in Mortality and Morbidity (CHARM) program. Arch Intern Med. 2008; 168:1699–704. https://doi.org/10.1001/archinte.168.15.1699 PMID:18695086

2. Cavero-Redondo I, Peleteiro B, Álvarez-Bueno C, Rodriguez-Artalejo F, Martínez-Vizcaíno V. Glycated haemoglobin A1c as a risk factor of cardiovascular outcomes and all-cause mortality in diabetic and non-diabetic populations: a systematic review and meta-analysis. BMJ Open. 2017; 7:e015949. https://doi.org/10.1136/bmjopen-2017-015949 PMID:28760792

3. Wong HK, Cheung TT, Cheung BM. Adrenomedullin and cardiovascular diseases. JRSM Cardiovasc Dis. 2012; 1. https://doi.org/10.1258/cvd.2012.012003 PMID:24175071

4. Larrayoz IM, Ferrero H, Martisova E, Gil-Bea FJ, Ramírez MJ, Martínez A. Adrenomedullin Contributes to Age-Related Memory Loss in Mice and Is Elevated in Aging Human Brains. Front Mol Neurosci. 2017; 10:384. https://doi.org/10.3389/fnmol.2017.00384 PMID:29187812

5. Liabeuf S, Lenglet A, Desjardins L, Neirynck N, Glorieux G, Lemke HD, Vanholder R, Diouf M, Choukroun G, Massy ZA, and European Uremic Toxin Work Group (EUTox). Plasma beta-2 microglobulin is associated with cardiovascular disease in uremic patients. Kidney Int. 2012; 82:1297–303. https://doi.org/10.1038/ki.2012.301 PMID:22895515

6. Smith LK, He Y, Park JS, Bieri G, Snethlage CE, Lin K, Gontier G, Wabl R, Plambeck KE, Udeochu J, Wheatley EG, Bouchard J, Eggel A, et al. β2-microglobulin is a systemic pro-aging factor that impairs cognitive function and neurogenesis. Nat Med. 2015; 21:932–7. https://doi.org/10.1038/nm.3898 PMID:26147761

7. Ferguson TW, Komenda P, Tangri N. Cystatin C as a biomarker for estimating glomerular filtration rate. Curr Opin Nephrol Hypertens. 2015; 24:295–300. https://doi.org/10.1097/MNH.0000000000000115 PMID:26066476

8. van der Laan SW, Fall T, Soumaré A, Teumer A, Sedaghat S, Baumert J, Zabaneh D, van Setten J, Isgum I, Galesloot TE, Arpegård J, Amouyel P, Trompet S, et al. Cystatin C and Cardiovascular Disease: A Mendelian Randomization Study. J Am Coll Cardiol. 2016; 68:934–45. https://doi.org/10.1016/j.jacc.2016.05.092 PMID:27561768

9. Paterson RW, Bartlett JW, Blennow K, Fox NC, Shaw LM, Trojanowski JQ, Zetterberg H, Schott JM, and Alzheimer's Disease Neuroimaging Initiative. Cerebrospinal fluid markers including trefoil factor 3 are associated with neurodegeneration in amyloid-positive individuals. Transl Psychiatry. 2014; 4:e419. https://doi.org/10.1038/tp.2014.58 PMID:25072324

10. Kaptoge S, Di Angelantonio E, Lowe G, Pepys MB, Thompson SG, Collins R, Danesh J, and Emerging Risk Factors Collaboration. C-reactive protein concentration and risk of coronary heart disease, stroke, and mortality: an individual participant meta-analysis. Lancet. 2010; 375:132–40. https://doi.org/10.1016/S0140-6736(09)61717-7 PMID:20031199

11. Shrivastava AK, Singh HV, Raizada A, Singh SK. C-reactive protein, inflammation and coronary heart disease. The Egyptian Heart Journal. 2015; 67:89–97. https://doi.org/10.1016/j.ehj.2014.11.005

12. Fujita Y, Taniguchi Y, Shinkai S, Tanaka M, Ito M. Secreted growth differentiation factor 15 as a potential biomarker for mitochondrial dysfunctions in aging and age-related disorders. Geriatr Gerontol Int. 2016 (Suppl 1); 16:17–29. https://doi.org/10.1111/ggi.12724 PMID:27018280

13. Maioli S, Lodeiro M, Merino-Serrais P, Falahati F, Khan W, Puerta E, Codita A, Rimondini R, Ramirez MJ, Simmons A, Gil-Bea F, Westman E, Cedazo-Minguez A, and Alzheimer's Disease Neuroimaging Initiative. Alterations in brain leptin signalling in spite of unchanged CSF leptin levels in Alzheimer's disease. Aging Cell. 2015; 14:122–9. https://doi.org/10.1111/acel.12281 PMID:25453257

14. Cesari M, Pahor M, Incalzi RA. Plasminogen activator inhibitor-1 (PAI-1): a key factor linking fibrinolysis and age-related subclinical and clinical conditions. Cardiovasc Ther. 2010; 28:e72–91. https://doi.org/10.1111/j.1755-5922.2010.00171.x PMID:20626406

15. As, Chao C, Borgmann K, Brew K, Ghorpade A. Tissue inhibitor of metalloproteinases-1 protects human neurons from staurosporine and HIV-1-induced

apoptosis: mechanisms and relevance to HIV-1-associated dementia. Cell Death Dis. 2012; 3:e332. https://doi.org/10.1038/cddis.2012.54 PMID:22739984

16. Dawber TR, Meadors GF, MOORE FE Jr. Epidemiological approaches to heart disease: the Framingham Study. Am J Public Health Nations Health. 1951; 41:279–81. https://doi.org/10.2105/ajph.41.3.279 PMID:14819398

17. Kannel WB, Feinleib M, McNamara PM, Garrison RJ, Castelli WP. An investigation of coronary heart disease in families. The Framingham offspring study. Am J Epidemiol. 1979; 110:281–90. https://doi.org/10.1093/oxfordjournals.aje.a112813 PMID:474565

18. Aryee MJ, Jaffe AE, Corrada-Bravo H, Ladd-Acosta C, Feinberg AP, Hansen KD, Irizarry RA. Minfi: a flexible and comprehensive Bioconductor package for the analysis of Infinium DNA methylation microarrays. Bioinformatics. 2014; 30:1363–9. https://doi.org/10.1093/bioinformatics/btu049 PMID:24478339

19. Design of the Women's Health Initiative clinical trial and observational study. The Women's Health Initiative Study Group. Control Clin Trials. 1998; 19:61–109. https://doi.org/10.1016/s0197-2456(97)00078-0 PMID:9492970

20. Anderson GL, Manson J, Wallace R, Lund B, Hall D, Davis S, Shumaker S, Wang CY, Stein E, Prentice RL. Implementation of the Women's Health Initiative study design. Ann Epidemiol. 2003; 13:S5–17. https://doi.org/10.1016/s1047-2797(03)00043-7 PMID:14575938

21. Whitsel E. Epigenetic Mechanisms of PM-Mediated CVD Risk (WHI-EMPC; R01-ES020836). National Institutes of Health US Department of Health and Human Services Research Portfolio Online Reporting Tools. 2016.

22. Teschendorff AE, Marabita F, Lechner M, Bartlett T, Tegner J, Gomez-Cabrero D, Beck S. A beta-mixture quantile normalization method for correcting probe design bias in Illumina Infinium 450 k DNA methylation data. Bioinformatics. 2013; 29:189–96. https://doi.org/10.1093/bioinformatics/bts680 PMID:23175756

23. Johnson WE, Li C, Rabinovic A. Adjusting batch effects in microarray expression data using empirical Bayes methods. Biostatistics. 2007; 8:118–27. https://doi.org/10.1093/biostatistics/kxj037 PMID:16632515

24. Lu AT, Xue L, Salfati EL, Chen BH, Ferrucci L, Levy D, Joehanes R, Murabito JM, Kiel DP, Tsai PC, Yet I, Bell JT, Mangino M, et al. GWAS of epigenetic aging rates in blood reveals a critical role for TERT. Nat Commun. 2018; 9:387. https://doi.org/10.1038/s41467-017-02697-5 PMID:29374233

25. Patterson RE, Kristal AR, Tinker LF, Carter RA, Bolton MP, Agurs-Collins T. Measurement characteristics of the Women's Health Initiative food frequency questionnaire. Ann Epidemiol. 1999; 9:178–87. https://doi.org/10.1016/s1047-2797(98)00055-6 PMID:10192650

26. Taylor HA Jr, Wilson JG, Jones DW, Sarpong DF, Srinivasan A, Garrison RJ, Nelson C, Wyatt SB. Toward resolution of cardiovascular health disparities in African Americans: design and methods of the Jackson Heart Study. Ethn Dis. 2005; 15:S6–4. PMID:16320381

27. Moore AZ, Hernandez DG, Tanaka T, Pilling LC, Nalls MA, Bandinelli S, Singleton AB, Ferrucci L. Change in Epigenome-Wide DNA Methylation Over 9 Years and Subsequent Mortality: Results From the InCHIANTI Study. J Gerontol A Biol Sci Med Sci. 2016; 71:1029–35. https://doi.org/10.1093/gerona/glv118 PMID:26355017

28. Zhou W, Triche TJ Jr, Laird PW, Shen H. SeSAMe: reducing artifactual detection of DNA methylation by Infinium BeadChips in genomic deletions. Nucleic Acids Res. 2018; 46:e123. https://doi.org/10.1093/nar/gky691 PMID:30085201

29. Ferrucci L. The Baltimore Longitudinal Study of Aging (BLSA): a 50-year-long journey and plans for the future. J Gerontol A Biol Sci Med Sci. 2008; 63:1416–9. https://doi.org/10.1093/gerona/63.12.1416 PMID:19126858

30. Kuo PL, Schrack JA, Shardell MD, Levine M, Moore AZ, An Y, Elango P, Karikkineth A, Tanaka T, de Cabo R, Zukley LM, AlGhatrif M, Chia CW, et al. A roadmap to build a phenotypic metric of ageing: insights from the Baltimore Longitudinal Study of Aging. J Intern Med. 2020; 287:373–94. https://doi.org/10.1111/joim.13024 PMID:32107805

31. Fabbri E, An Y, Zoli M, Simonsick EM, Guralnik JM, Bandinelli S, Boyd CM, Ferrucci L. Aging and the burden of multimorbidity: associations with inflammatory and anabolic hormonal biomarkers. J Gerontol A Biol Sci Med Sci. 2015; 70:63–70. https://doi.org/10.1093/gerona/glu127 PMID:25104822

32. Deary IJ, Gow AJ, Pattie A, Starr JM. Cohort profile: the Lothian Birth Cohorts of 1921 and 1936. Int J Epidemiol. 2012; 41:1576–84. https://doi.org/10.1093/ije/dyr197 PMID:22253310

33. Taylor AM, Pattie A, Deary IJ. Cohort Profile Update: The Lothian Birth Cohorts of 1921 and 1936. Int J Epidemiol. 2018; 47:1042–2r. https://doi.org/10.1093/ije/dyy022 PMID:29546429

34. Marioni RE, Shah S, McRae AF, Chen BH, Colicino E, Harris SE, Gibson J, Henders AK, Redmond P, Cox SR, Pattie A, Corley J, Murphy L, et al. DNA methylation age of blood predicts all-cause mortality in later life. Genome Biol. 2015; 16:25. https://doi.org/10.1186/s13059-015-0584-6 PMID:25633388

35. Pidsley R, Y Wong CC, Volta M, Lunnon K, Mill J, Schalkwyk LC. A data-driven approach to preprocessing Illumina 450K methylation array data. BMC Genomics. 2013; 14:293. https://doi.org/10.1186/1471-2164-14-293 PMID:23631413

36. Bell B, Rose CL, Damon A. The Veterans Administration longitudinal study of healthy aging. Gerontologist. 1966; 6:179–84. https://doi.org/10.1093/geront/6.4.179 PMID:5342911

37. Dai L, Mehta A, Mordukhovich I, Just AC, Shen J, Hou L, Koutrakis P, Sparrow D, Vokonas PS, Baccarelli AA, Schwartz JD. Differential DNA methylation and $PM_{2.5}$ species in a 450K epigenome-wide association study. Epigenetics. 2017; 12:139–48. https://doi.org/10.1080/15592294.2016.1271853 PMID:27982729

38. Wang C, Cardenas A, Hutchinson JN, Just A, Heiss J, Hou L, Zheng Y, Coull BA, Kosheleva A, Koutrakis P, Baccarelli AA, Schwartz JD. Short- and intermediate-term exposure to ambient fine particulate elements and leukocyte epigenome-wide DNA methylation in older men: the Normative Aging Study. Environ Int. 2022; 158:106955. https://doi.org/10.1016/j.envint.2021.106955 PMID:34717175

39. Hamlat EJ, Adler NE, Laraia B, Surachman A, Lu AT, Zhang J, Horvath S, Epel ES. Association of subjective social status with epigenetic aging among Black and White women. Psychoneuroendocrinology. 2022; 141:105748. https://doi.org/10.1016/j.psyneuen.2022.105748 PMID:35397259

40. Houseman EA, Accomando WP, Koestler DC, Christensen BC, Marsit CJ, Nelson HH, Wiencke JK, Kelsey KT. DNA methylation arrays as surrogate measures of cell mixture distribution. BMC Bioinformatics. 2012; 13:86. https://doi.org/10.1186/1471-2105-13-86 PMID:22568884

41. Horvath S. DNA methylation age of human tissues and cell types. Genome Biol. 2013; 14:R115. https://doi.org/10.1186/gb-2013-14-10-r115 PMID:24138928

42. Horvath S, Levine AJ. HIV-1 Infection Accelerates Age According to the Epigenetic Clock. J Infect Dis. 2015; 212:1563–73. https://doi.org/10.1093/infdis/jiv277 PMID:25969563

43. Horvath S, Gurven M, Levine ME, Trumble BC, Kaplan H, Allayee H, Ritz BR, Chen B, Lu AT, Rickabaugh TM, Jamieson BD, Sun D, Li S, et al. An epigenetic clock analysis of race/ethnicity, sex, and coronary heart disease. Genome Biol. 2016; 17:171. https://doi.org/10.1186/s13059-016-1030-0 PMID:27511193

44. Langfelder P, Horvath S. WGCNA: an R package for weighted correlation network analysis. BMC Bioinformatics. 2008; 9:559. https://doi.org/10.1186/1471-2105-9-559 PMID:19114008
