# Supplementary material for: DNA methylation GrimAge version 2
Source: Aging (Albany NY). 2022 Dec 14;14(23):9484–549. doi: 10.18632/aging.204434 (PMC9792204; doi:10.18632/aging.204434)
Supplement: Supplementary Figures [file aging-14-204434-s002.pdf]

## SUPPLEMENTARY FIGURES

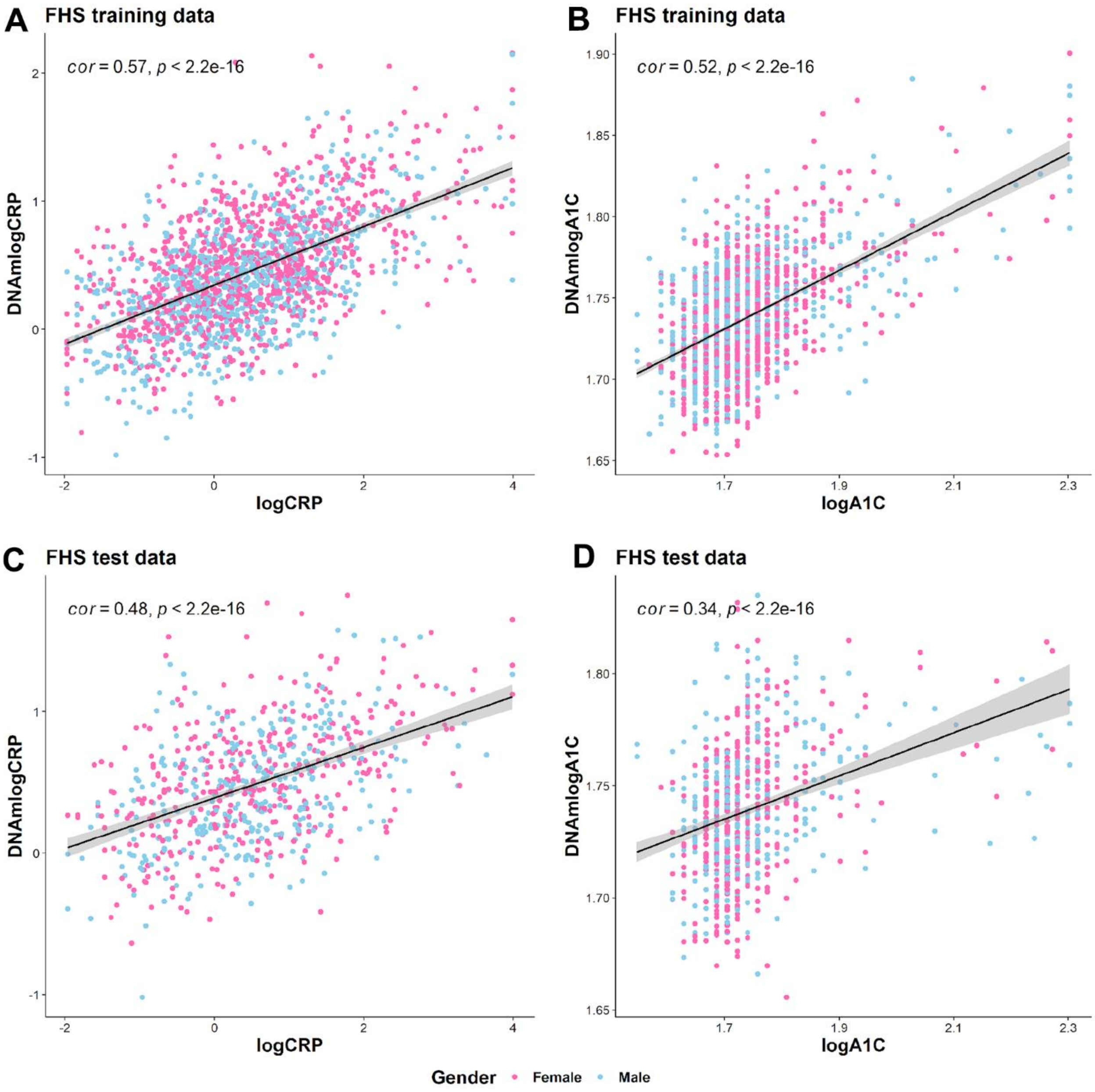


**Supplementary Figure 1. New DNAm proteins.** The top panels (**A**, **B**) and bottom panels (**C**, **D**) are based on FHS training and test dataset, respectively. (**A**, **C**) The panels depict scatter plots of log scale of C-reactive protein (CRP, x-axis) versus DNAmlogCRP (y-axis). (**B**, **D**) The panels depict scatter plots of log scale of hemoglobin A1C (x-axis) versus DNAmlogA1C (y-axis). The title of each panel reports the data set. The Pearson correlation coefficient (cor) and a corresponding correlation test p-value are report at each panel. The top panels are based on the training dataset (70% pedigrees) of Framingham Heart Study (FHS) pedigree data that were used to develop DNAm based biomarkers. The bottom panels are based on FHS test dataset with individuals from the remaining 30% pedigrees to test the predictive power of the DNAm biomarkers. The extreme values for the CRP and A1C variables were defined if their scaled values were ≥ 6 and were winsorized before the training process. The plots depict the log scale applying on the variables after winsorization.

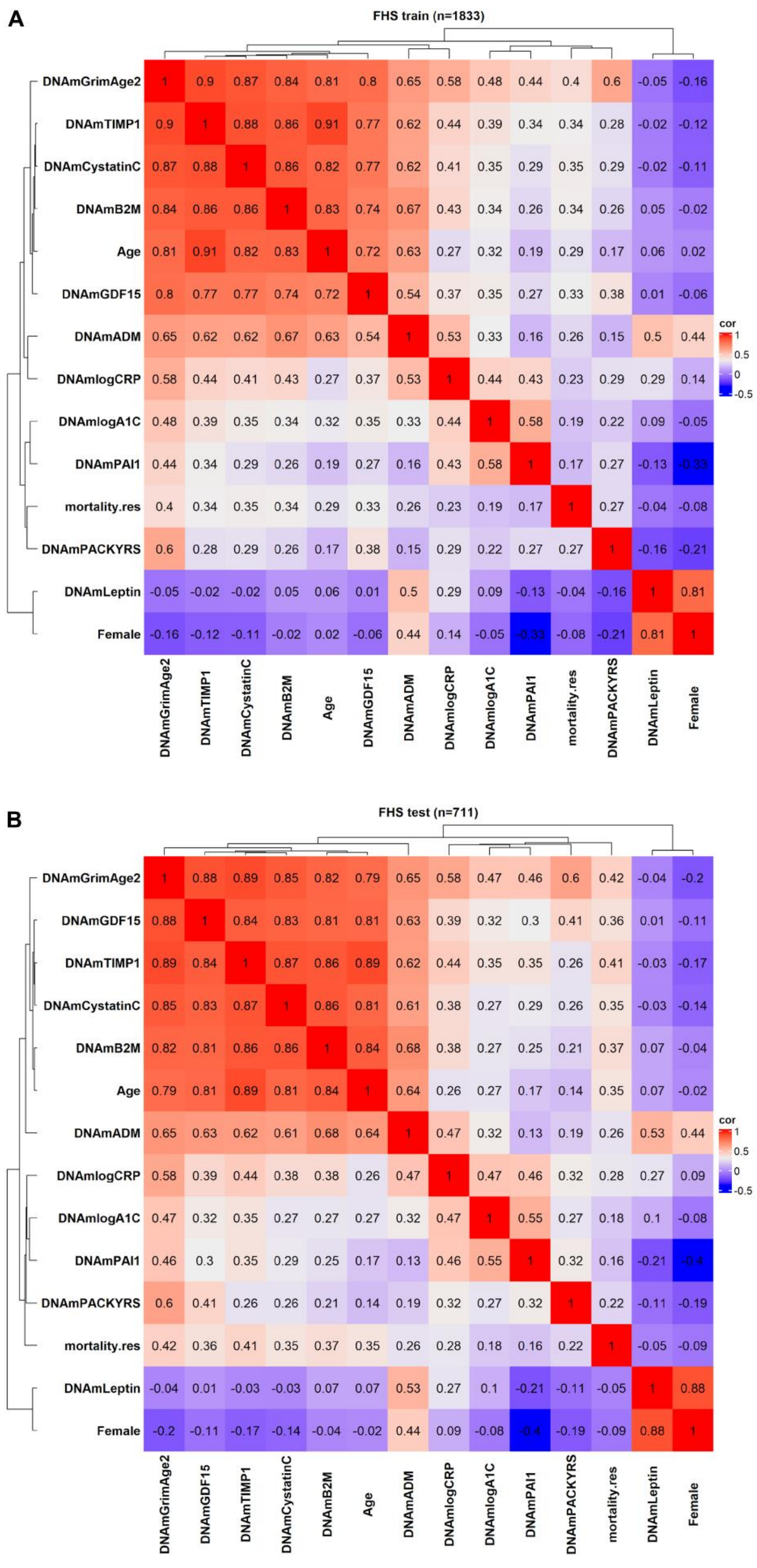


**Supplementary Figure 2. Correlation heatmap among DNAmGrimAge2.** The heatmap color-codes the pairwise Pearson correlations of DNAmGrimAge2 and its 10 components: (**A**) the heatmap based on the training dataset in FHS (n=1833), and (**B**) the heatmap based on the

test dataset in FHS (n=711). DNAm GrimAge2 is defined as a linear combination of chronological age (Age), sex (Female takes on the value 1 for females and 0 otherwise), and ten DNAm-based surrogate markers for smoking pack-years (DNAm PACKYRS), adrenomedullin levels (DNAm ADM), beta-2 microglobulin (DNAm B2M), cystatin C (DNAm Cystatin C), growth differentiation factor 15 (DNAm GDF-15), leptin (DNAm Leptin), ) log scale of C reactive protein (CRP), log scale of hemoglobin A1C,plasminogen activation inhibitor 1 (DNAm PAI-1), and tissue inhibitor metalloproteinase 1 (DNAm TIMP-1). The figure also includes an estimator of mortality risk, *mortality.res*, which can be interpreted as a measure of “excess” mortality risk compared to the baseline risk in the study data. Formally, mortality.res is defined as the deviance residual from a Cox regression model for time-to-death due to all-cause mortality. The rows and columns of the figure are sorted according to a hierarchical clustering tree. The shades of color (blue, white, and red) visualize correlation values from -1 to 1. Each square reports a Pearson correlation coefficient.

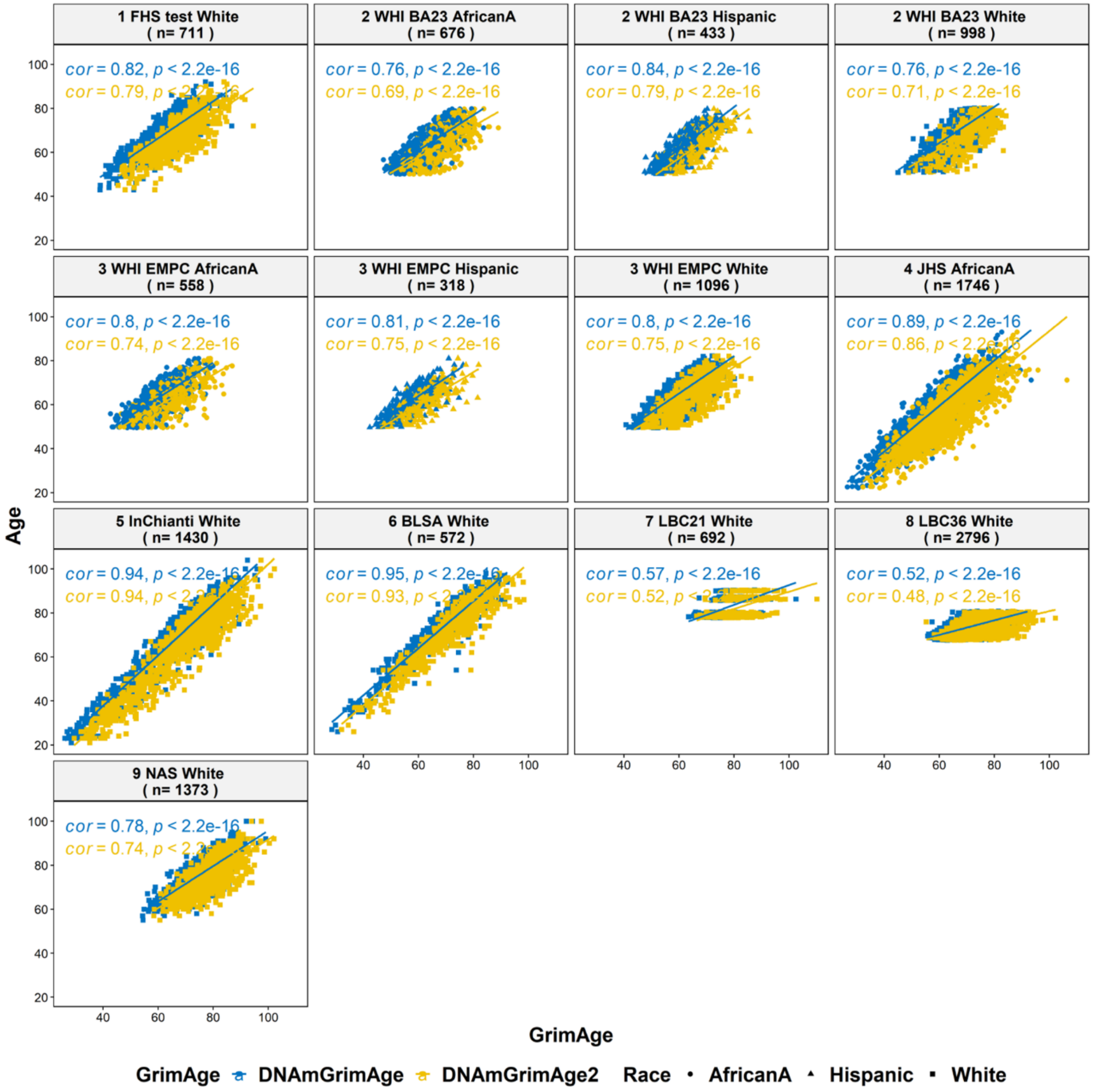


**Supplementary Figure 3. DNAm GrimAge(2) versus chronological age in different study cohorts.** Each panel depicts a scatter plot of GrimAge2/GrimAge (x-axis) versus chronological age at the time of the blood draw (x-axis). The title of each panel reports the data set and the sample size. The plots of the WHI cohorts are stratified by race/ethnic groups. The statistics Pearson correlation coefficient, and a corresponding correlation test p-value are reported at each panel stratified by DNAmGrimAge2 and DNAmGrimAge, respectively. Each point is marked by blue for DNAmGrimAge and yellow for DNAmGrimAge2, with a point shape based on race/ethnicity, as listed in legend. AfricanA denotes African American.

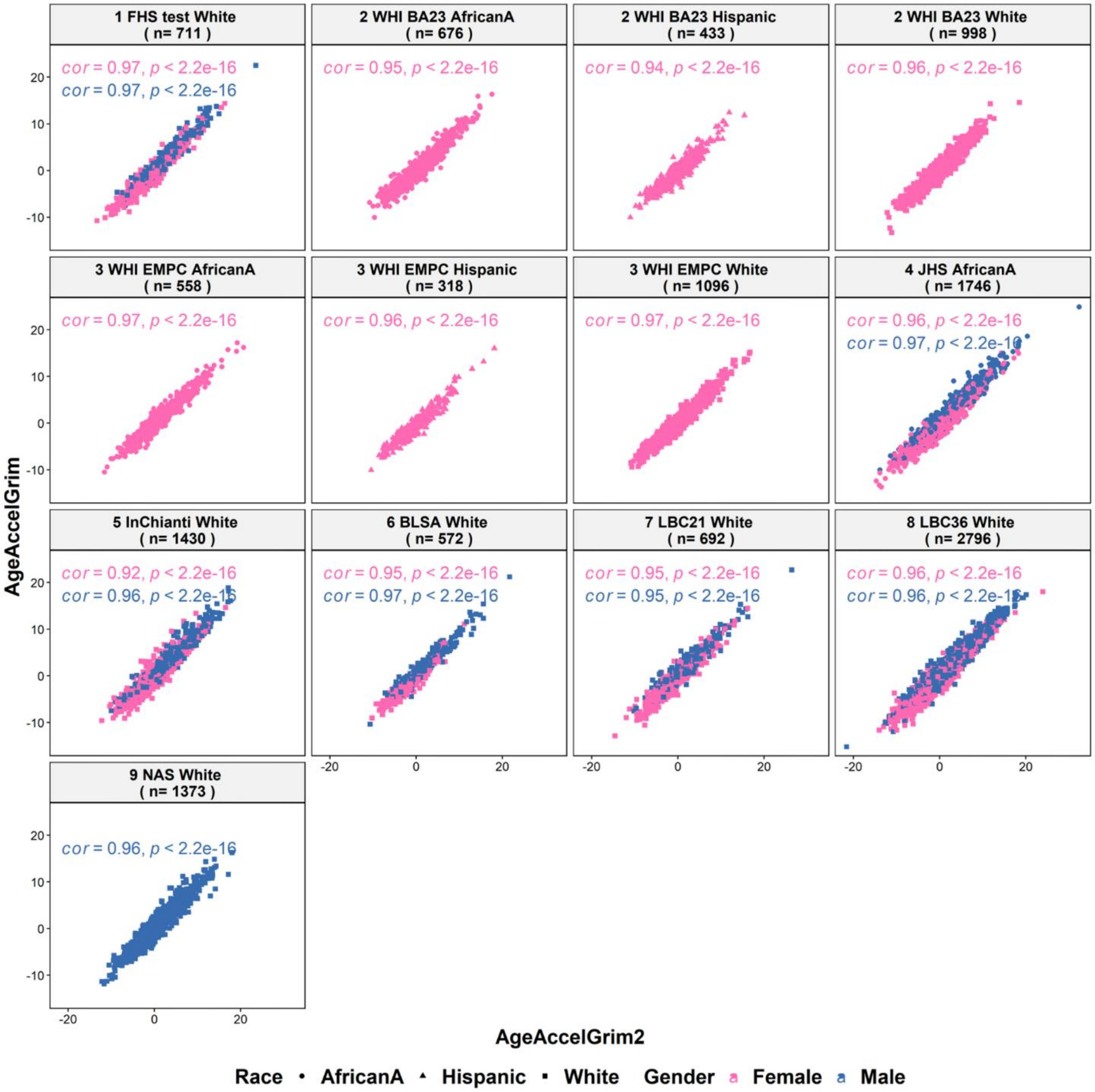


**Supplementary Figure 4. AgeAccelGrim2 versus AgeAccelGrim in different study cohorts.** Each panel depicts a scatter plot of AgeAccelGrim2(x-axis) versus AgeAccelGrim (y-axis) at the time of the blood draw. The title of each panel reports the data set and the sample size. The plots of the WHI cohorts are stratified by race/ethnic groups. The statistics Pearson correlation coefficient, and a corresponding correlation test p-value are reported at each panel stratified by gender. Each point is marked by blue for males and hot pink for females, with a point shape based on race/ethnicity, as listed in legend. AfricanA denotes African American.

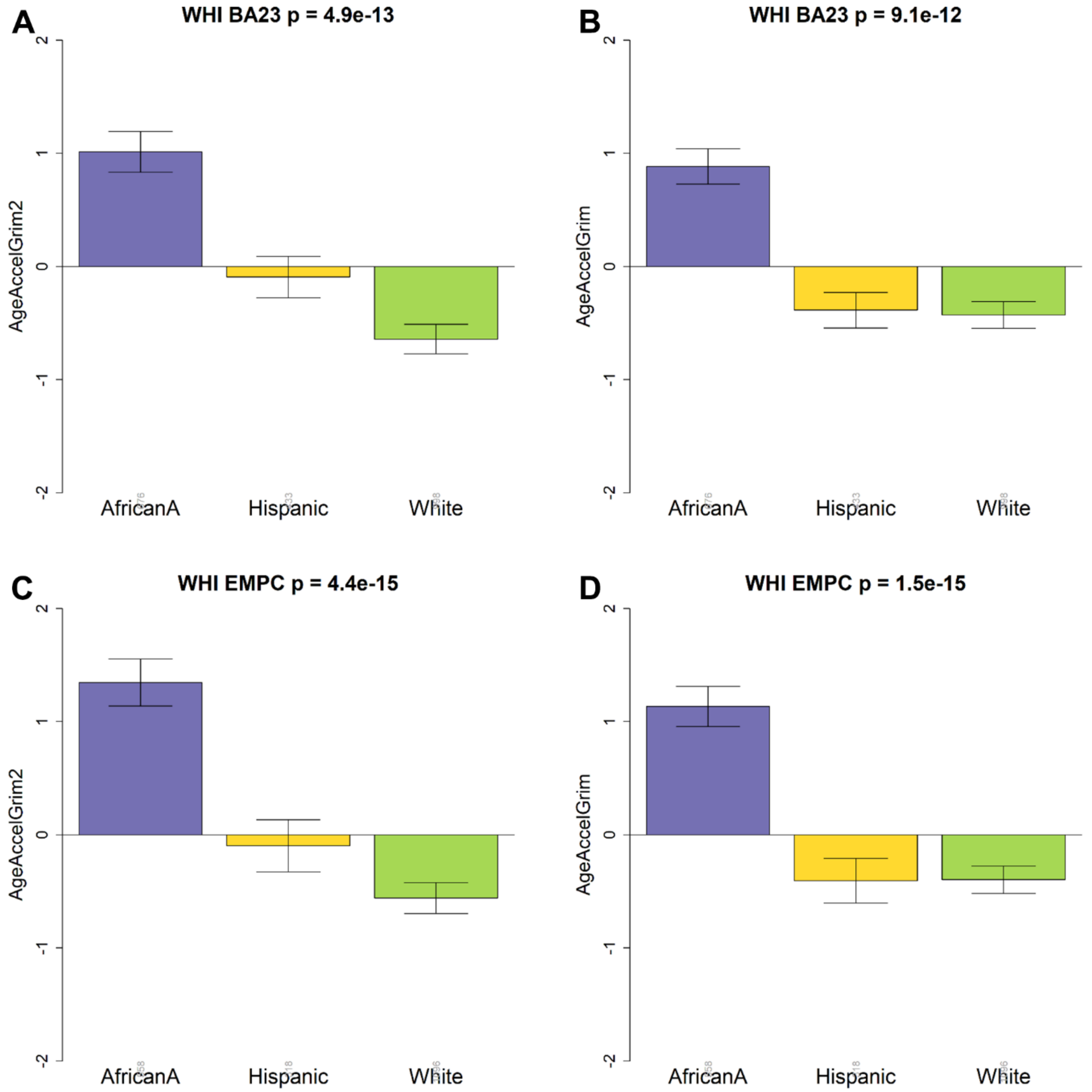


**Supplementary Figure 5. Association between epigenetic age acceleration of GrimAges versus ethnicity.** The figure presents bar plots for the associations between AgeAccelGrim2/AgeAccelGrim (y-axis) and three racial/ethnic group: African American (AfricanA), Hispanic and White. The upper (**A**, **B**)/lower (**C**, **D**) panels are based on WHI BA23/WHI EMPC datasets, respectively. The left/right panels display AgeAccelGrim2/AgeAccelGrim on y-axis, respectively. The bar plots report the p-value of a non-parametric group comparison test (Kruskal-Wallis). The y-axis of the bar plots depicts the mean and one standard error. The number under each bar presents number of individuals at each racial group.

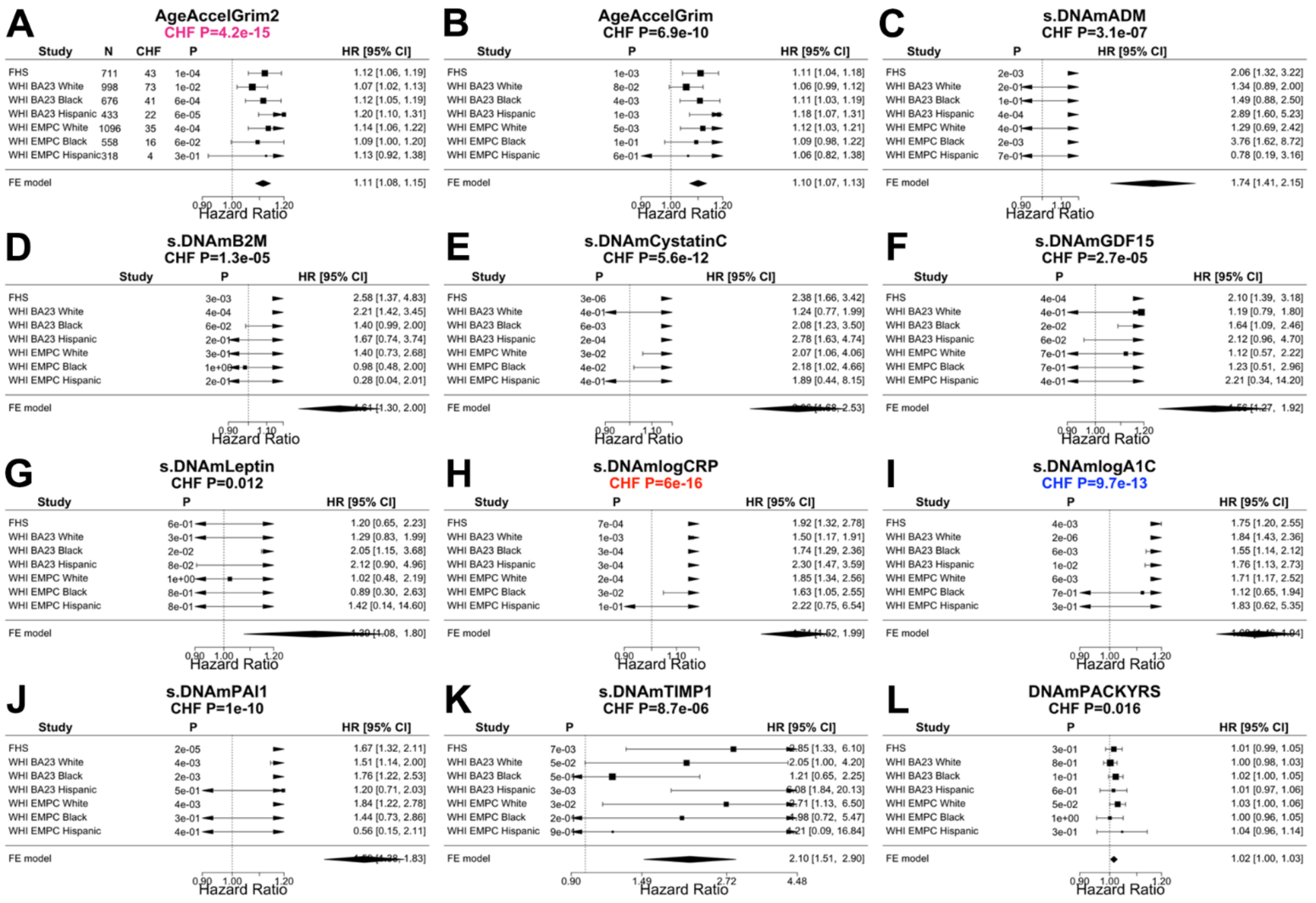

**Supplementary Figure 6. Meta analysis forest plots for predicting time-to-congestive heart failure.** Fixed effect models meta analysis was performed to combine Cox regression analysis of congestive heart failure (CHF) across 7 strata from 3 study cohorts. Each panel reports a meta analysis forest plot for combining hazard ratios predicting time-to-CHF based on a DNAm based biomarker (reported in the figure heading) across different strata formed by racial group within cohort. (**A**, **B**) display the results for AgeAccelGrim2 and AgeAccelGrim. Each row reports a hazard ratio (for time-to-CHF) and a 95% confidence interval resulting from a Cox regression model in each stratum. (**C**–**L**) display the results for (age-adjusted) DNAm based surrogate markers of (**C**) adrenomedullin (ADM), (**D**) beta-2 microglobulin (B2M), (**E**) cystatin C (Cystatin C), (**F**) growth differentiation factor 15 (GDF-15), (**G**) leptin, (**H**) log scale of C reactive protein (CRP), (**I**) log scale of hemoglobin A1C, (**J**) plasminogen activation inhibitor 1 (PAI-1), (**K**) tissue inhibitor metalloproteinase 1 (TIMP-1) and (**L**) smoking pack-years (PACKYRS). The sub-title of each panel reports the meta analysis P-value. (**A**, **B**) Each hazard ratio (HR) corresponds to a one-year increase in AgeAccel. (**C**–**K**) Each hazard ratio corresponds to an increase in one-standard deviation. (**L**) Hazard ratios correspond to a one-year increase in pack-years. The most significant meta analysis P-value is marked in red (DNAm logCRP), followed by hot pink (AgeAccelGrim2) and blue (DNAm logA1C), respectively.

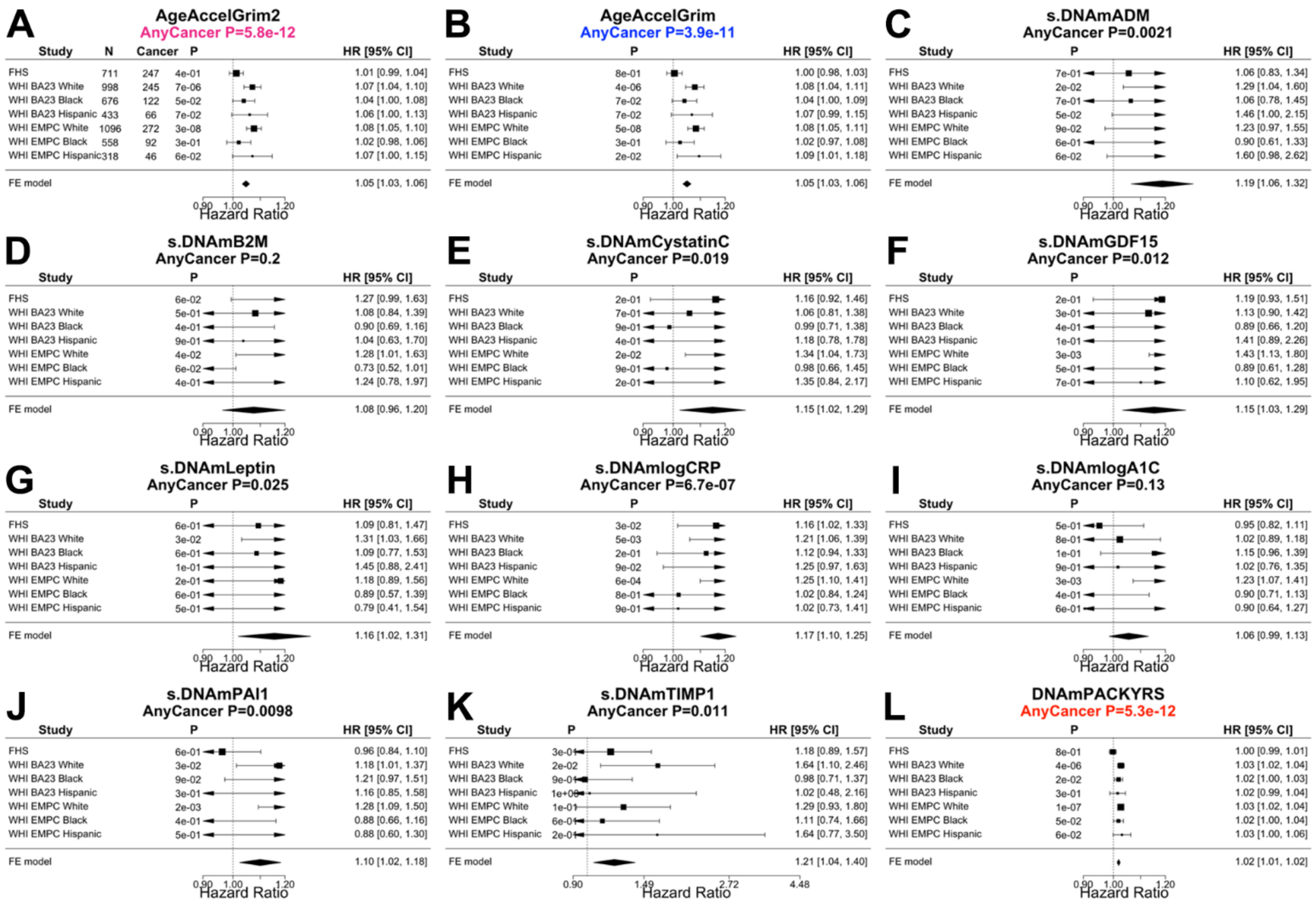

**Supplementary Figure 7. Meta analysis forest plots for predicting time-to-any cancer.** Fixed effect models meta analysis was performed to combine Cox regression analysis of any cancer across 7 strata from 3 study cohorts. Each panel reports a meta analysis forest plot for combining hazard ratios predicting time-to-any cancer based on a DNAm based biomarker (reported in the figure heading) across different strata formed by racial group within cohort. (**A**, **B**) display the results for AgeAccelGrim2 and AgeAccelGrim. Each row reports a hazard ratio (for time-to-any cancer) and a 95% confidence interval resulting from a Cox regression model in each strata (defined by cohort racial group). (**C**–**L**) display the results for (age-adjusted) DNAm based surrogate markers of (**C**) adrenomedullin (ADM), (**D**) beta-2 microglobulin (B2M), (**E**) cystatin C (Cystatin C), (**F**) growth differentiation factor 15 (GDF-15), (**G**) leptin, (**H**) log scale of C reactive protein (CRP), (**I**) log scale of hemoglobin A1C, (**J**) plasminogen activation inhibitor 1 (PAI-1), (**K**) tissue inhibitor metalloproteinase 1 (TIMP-1) and (**L**) smoking pack-years (PACKYRS). The sub-title of each panel reports the meta analysis p-value. (**A**, **B**) Each hazard ratio (HR) corresponds to a one-year increase in AgeAccelGrim. (**C**–**K**) Each hazard ratio corresponds to an increase in one-standard deviation. (**L**) Hazard ratios correspond to a 1 year increase in pack-years. A *non*-significant Cochran Q test p-value is desirable because it indicates that the hazard ratios don't differ significantly across the strata. The most significant meta analysis P value is marked in red (DNAm PACKYRS), followed by hot pink (AgeAccelGrim2) and blue (AgeAccelGrim), respectively.

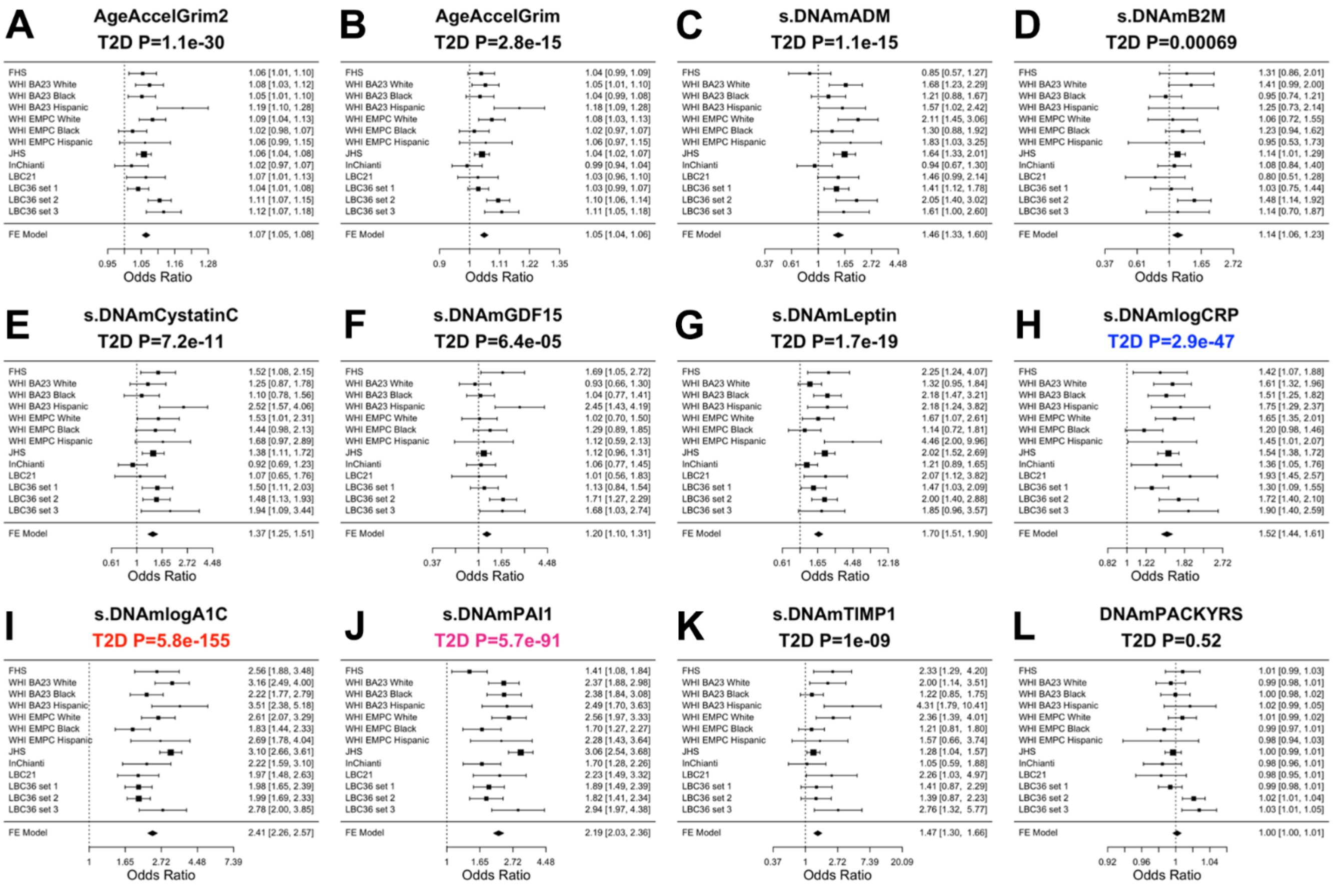


**Supplementary Figure 8. Meta analysis for associations with Type 2 diabetes.** Each panel reports a meta analysis forest plot based on Stouffer's method to combine association results between disease free status and the DNAm-based biomarker (reported in the figure heading) across different strata, which are formed by racial group within cohort. (**A**, **B**) displays the results for AgeAccelGrim2 and AgeAccelGrim. (**C**–**L**) display the results for scaled DNAm based surrogate markers of (**C**) adrenomedullin (ADM), (**D**) beta-2 microglobulin (B2M), (**E**) cystatin C (Cystatin C), (**F**) growth differentiation factor 15 (GDF-15), (**G**) leptin, (**H**) log scale of C reactive protein (CRP), (**I**) log scale of hemoglobin A1C, (**J**) plasminogen activation inhibitor 1 (PAI-1), (**K**) tissue inhibitor metalloproteinase 1 (TIMP-1) and (**L**) smoking pack-years (PACKYRS). The sub-title of each panel reports the meta analysis p-value. Each row reports an odds ratio (OR) and a 95% confidence interval resulting from a (GEE) logistic regression in each strata (defined by cohort racial or set group). (**A**, **B**) Each OR corresponds to a one-year increase in AgeAccel. (**C**–**K**) Each OR corresponds to an increase in one-standard deviation. (**L**) OR corresponds to a one-year increase in pack-years. The most significant meta analysis P-value is marked in red (DNAm logA1C), followed by hot pink (DNAm PAI1) and blue (DNAm logCRP), respectively.

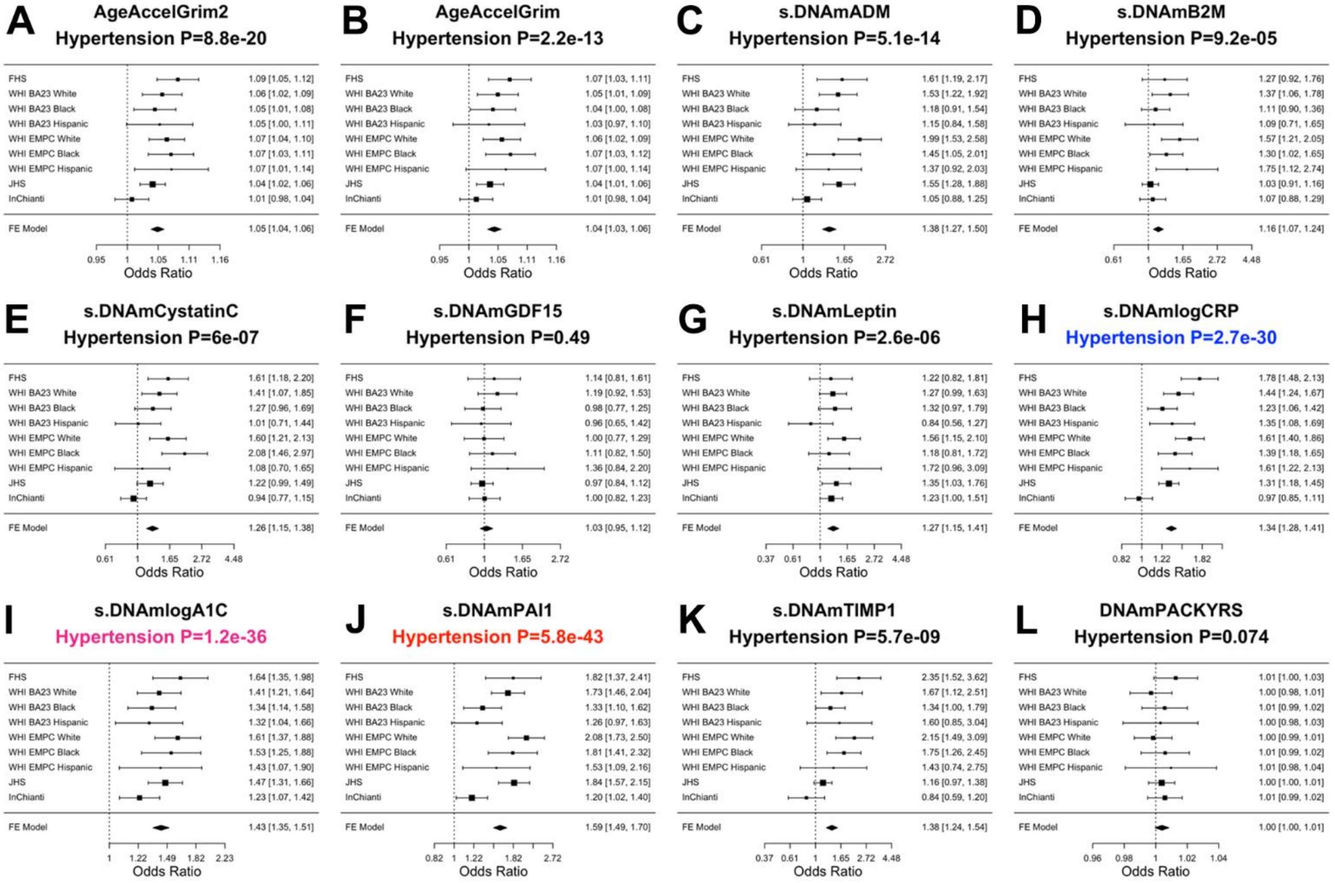


**Supplementary Figure 9. Meta analysis for associations with hypertension.** Each panel reports a meta analysis forest plot based on Stouffer's method to combine association results between disease free status and the DNAm-based biomarker (reported in the figure heading) across different strata, which are formed by racial group within cohort. (**A**, **B**) display the results for AgeAccelGrim2 and AgeAccelGrim. (**C**–**L**) display the results for scaled DNAm based surrogate markers of (**C**) adrenomedullin (ADM), (**D**) beta-2 microglobulin (B2M), (**E**) cystatin C (Cystatin C), (**F**) growth differentiation factor 15 (GDF-15), (**G**) leptin, (**H**) log scale of C reactive protein (CRP), (**I**) log scale of hemoglobin A1C, (**J**) plasminogen activation inhibitor 1 (PAI-1), (**K**) tissue inhibitor metalloproteinase 1 (TIMP-1) and (**L**) smoking pack-years (PACKYRS). The sub-title of each panel reports the meta analysis p-value. Each row reports an odds ratio (OR) and a 95% confidence interval resulting from a (GEE) logistic regression in each stratum (defined by cohort racial group). (**A**, **B**) Each OR corresponds to a one-year increase in AgeAccel. (**C**–**K**) Each OR corresponds to an increase in one-standard deviation. (**L**) OR corresponds to a one-year increase in pack-years. The most significant meta analysis P-value is marked in red (DNAm PAI-1), followed by hot pink (DNAm logA1C) and blue (DNAm logCRP), respectively.

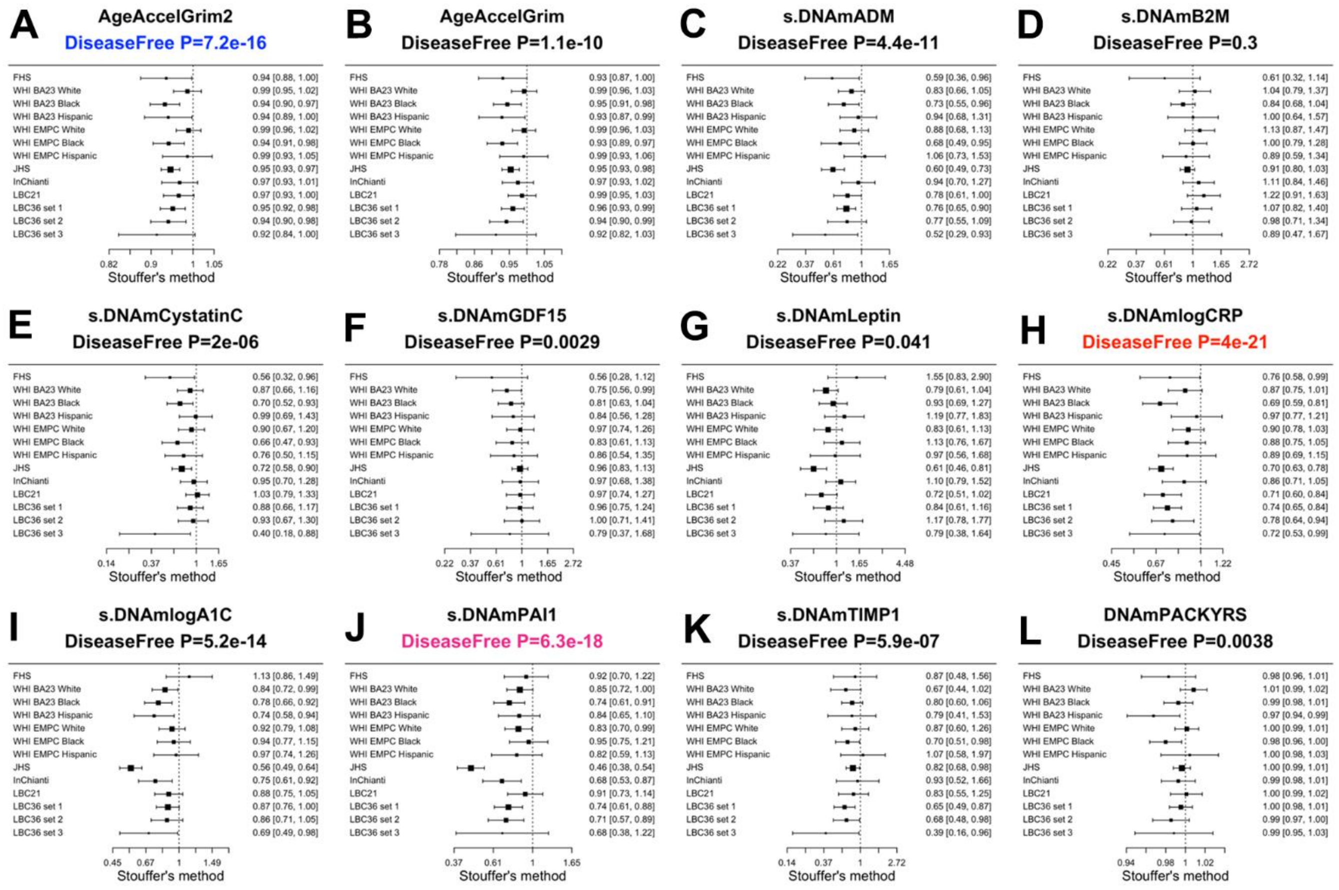

**Supplementary Figure 10. Meta analysis for associations with disease free status.** Each panel reports a meta analysis forest plot based on Stouffer's method to combine association results between disease free status and the DNAm-based biomarker (reported in the figure heading) across different strata, which are formed by racial group within cohort. (**A**, **B**) display the results for AgeAccelGrim2 and AgeAccelGrim. (**C**–**L**) display the results for scaled DNAm based surrogate markers of (**C**) adrenomedullin (ADM), (**D**) beta-2 microglobulin (B2M), (**E**) cystatin C (Cystatin C), (**F**) growth differentiation factor 15 (GDF-15), (**G**) leptin, (**H**) log scale of C reactive protein (CRP), (**I**) log scale of hemoglobin A1C, (**J**) plasminogen activation inhibitor 1 (PAI-1), (**K**) tissue inhibitor metalloproteinase 1 (TIMP-1) and (**L**) smoking pack-years (PACKYRS). The sub-title of each panel reports the meta analysis p-value. Each row reports an odds ratio (OR) and a 95% confidence interval resulting from a (GEE) logistic regression in each strata (defined by cohort racial/set group). (**A**, **B**) Each OR corresponds to a one-year increase in AgeAccel. (**C**–**K**) Each OR corresponds to an increase in one-standard deviation. (**L**) OR corresponds to a one-year increase in pack-years. The most significant meta analysis P-value is marked in red (DNAm logCRP), followed by hot pink (DNAm PAI1) and blue (AgeAccelGrim2), respectively.

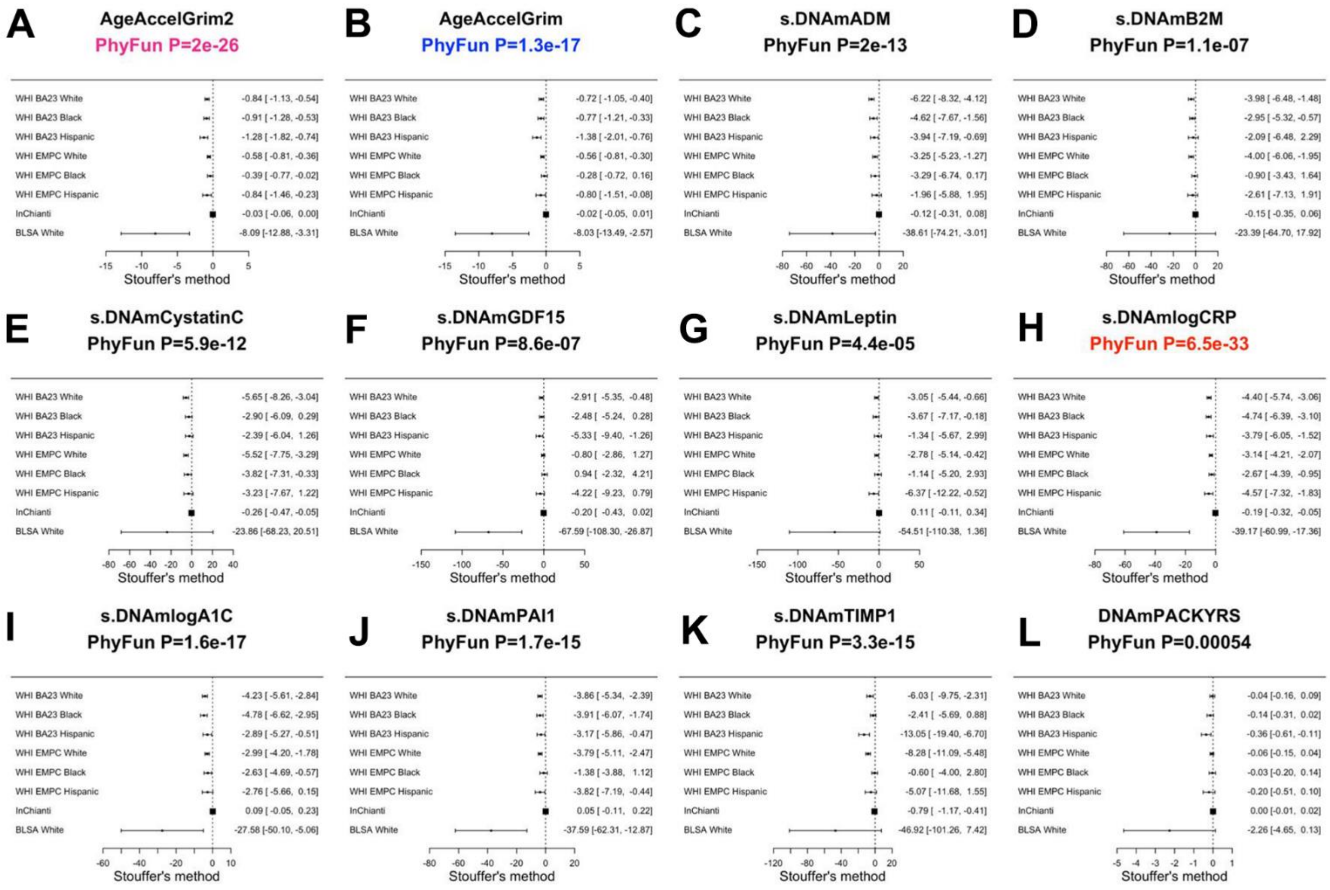


**Supplementary Figure 11. Meta analysis for associations with physical functioning level.** Each panel reports a meta analysis forest plot based on Stouffer's method to combine association results between physical functioning levels (dependent variable) and the DNAm-based biomarker (independent variable, reported in the figure heading) across different strata, which are formed by racial group within cohort. (**A**, **B**) display the results for AgeAccelGrim2 and AgeAccelGrim. (**C**–**L**) display the results for scaled DNAm based surrogate markers of (**C**) adrenomedullin (ADM), (**D**) beta-2 microglobulin (B2M), (**E**) cystatin C (Cystatin C), (**F**) growth differentiation factor 15 (GDF-15), (**G**) leptin, (**H**) log scale of C reactive protein (CRP), (**I**) log scale of hemoglobin A1C, (**J**) plasminogen activation inhibitor 1 (PAI-1), (**K**) tissue inhibitor metalloproteinase 1 (TIMP-1) and (**L**) smoking pack-years (PACKYRS). The sub-title of each panel reports the meta analysis p-value. Each row reports a beta coefficient β and a 95% confidence interval resulting from a (linear-mixed) regression model in each stratum (defined by cohort racial group). (**A**, **B**) Each β corresponds to a one-year increase in AgeAccel. (**C**–**K**) Each β corresponds to an increase in one-standard deviation. (**L**) β corresponds to a one-year increase in pack-years. The most significant meta analysis P-value is marked in red (DNAm logCRP), followed by hot pink (AgeAccelGrim2) and blue (AgeAccelGrim), respectively.

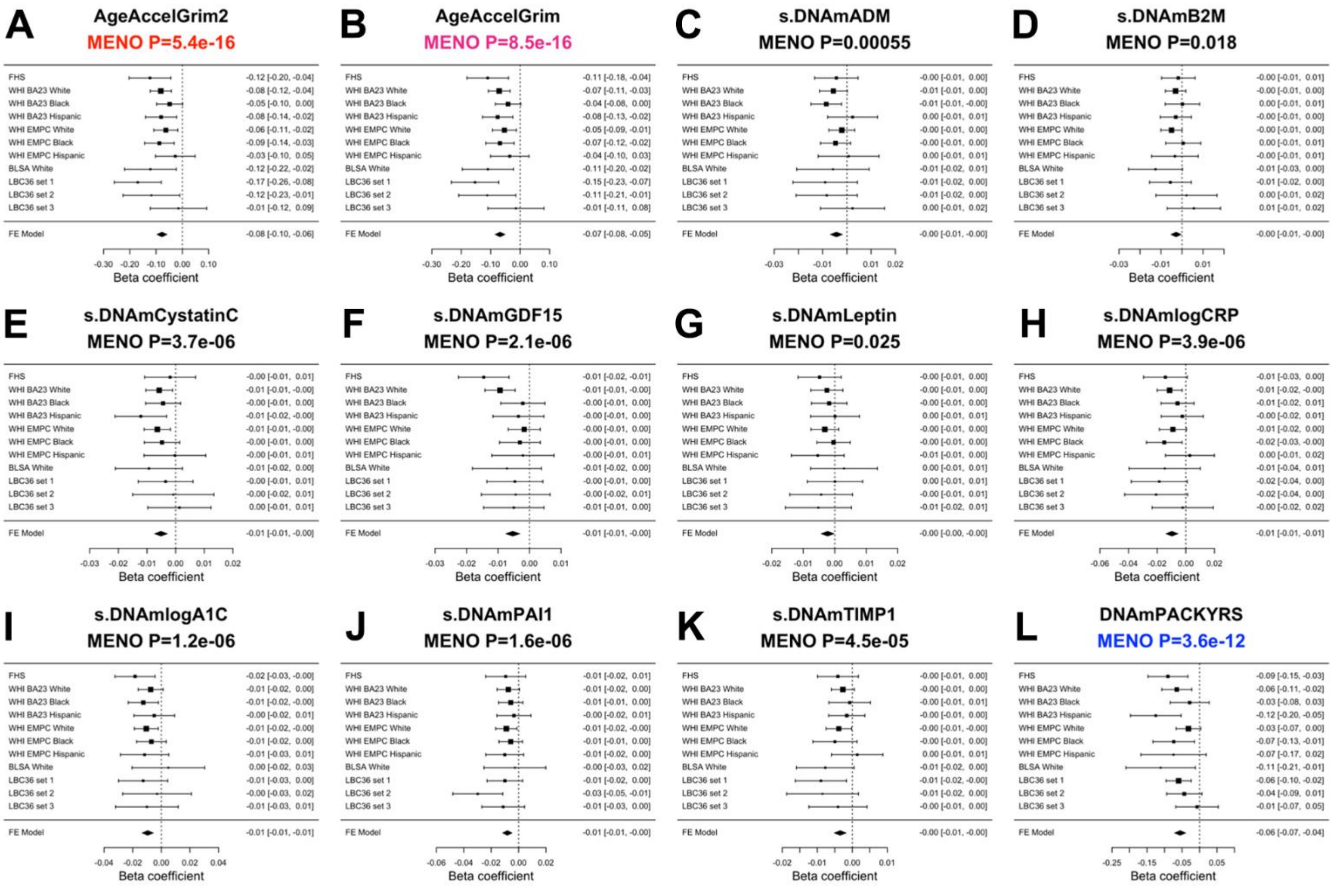


**Supplementary Figure 12. Meta analysis of age-at-menopause.** Each panel reports a meta analysis forest plot for combining regression coefficients (slopes) between age-at-menopause in women and the DNAm based biomarker (reported in the figure heading) across different strata, which are formed by racial group within cohort. Age at menopause was treated as independent variable as a causal effect on DNAm variables except for the association with DNAm PACKYRS, in which the pack year variable (independent variable) was used to predict age at menopause (dependent variable). (**A**, **B**) display the results for AgeAccelGrim2 and AgeAccelGrim. (**C**–**L**) display the results for scaled DNAm based surrogate markers of (**C**) adrenomedullin (ADM), (**D**) beta-2 microglobulin (B2M), (**E**) cystatin C (Cystatin C), (**F**) growth differentiation factor 15 (GDF-15), (**G**) leptin, (**H**) log scale of C reactive protein (CRP), (**I**) log scale of hemoglobin A1C, (**J**) plasminogen activation inhibitor 1 (PAI-1), (**K**) tissue inhibitor metalloproteinase 1 (TIMP-1) and (**L**) smoking pack-years (PACKYRS). The sub-title of each panel reports the meta analysis P-value. Each row reports a beta coefficient β and a 95% confidence interval resulting from a (linear-mixed) regression model in each stratum (defined by cohort racial group). Each β corresponds to a one-year late of age at menopause except for the regression analysis with respect to DNAm PACKYRS, in which β corresponds to a one-year increase in pack years. The most significant meta analysis P-value is marked in red (AgeaccelGrim2), followed by hot pink (AgeAccelGrim) and blue (DNAm PACKYRS), respectively.

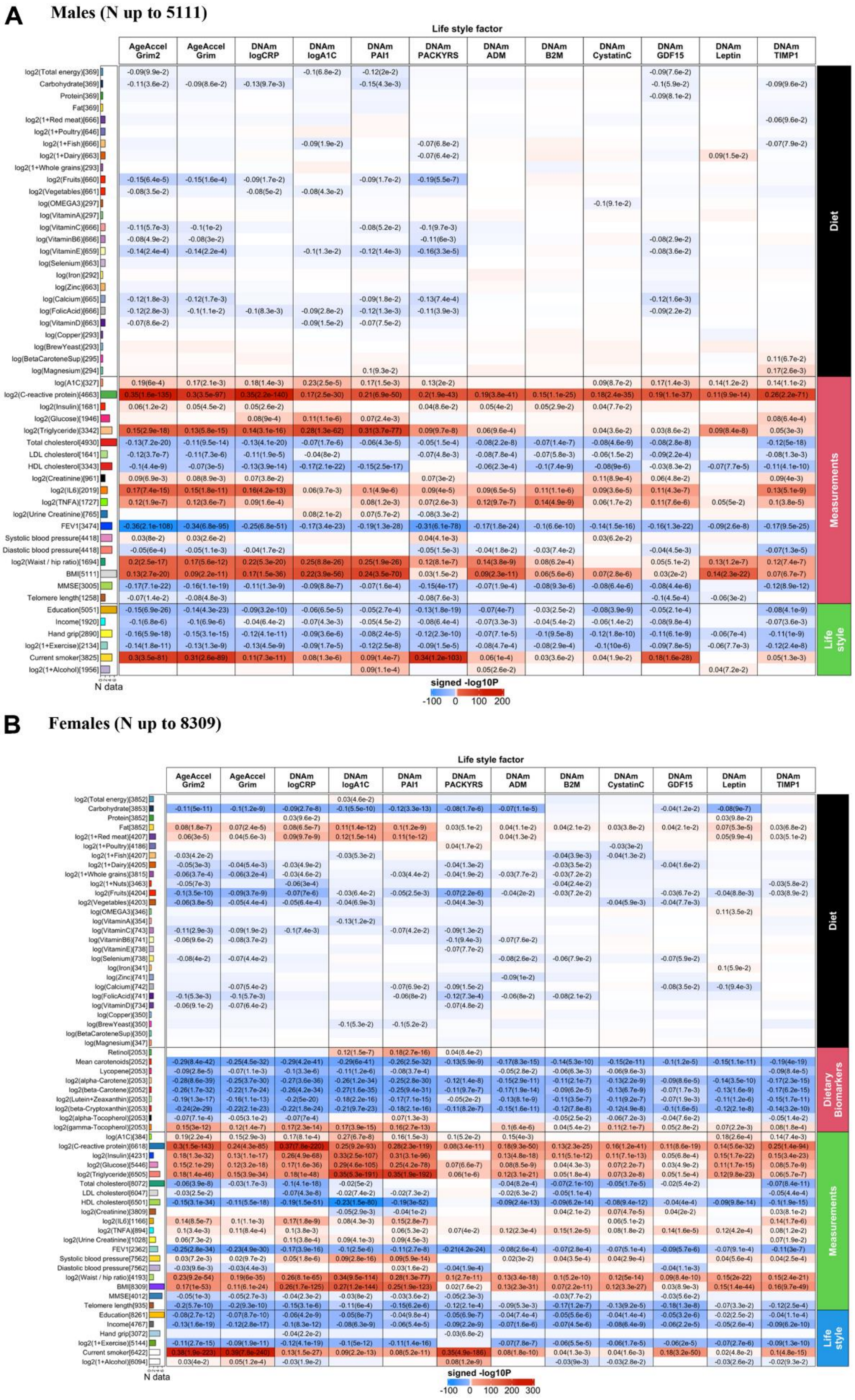


**Supplementary Figure 13. Meta cross sectional correlations between DNAm biomarkers and lifestyle factors.** Robust correlation coefficients (biweight midcorrelation [44]) between 1) AgeAccelGrim2, AgeAccelGrim, and ten age-adjusted underlying DNAm-

based surrogate biomarkers underlying DNAmGrimAge2, and 2) up to 61variables including 27 self-reported diet, 9 dietary biomarkers, 19 clinically relevant measurements related to vital signs, metabolic traits, inflammatory markers, cognitive function, lung function, central adiposity and leukocyte telomere length, and 6 life style factors including hand grip. Analysis was stratified by (**A**) Males and (**B**) Females, respectively. The y-axis lists lifestyle factor in the format of name (sample size), followed by a bar plot denoting number of studies. Variables are arranged by category displayed on the right annotation. The x-axis lists AgeAccelGrim2, AgeAccelGrim, followed by DNAm variables. The first few DNAm variables (log CRP, log A1C, PAI-1, smoking pack years) show strong correlation with the lifestyle factors in overall. Each cell presents meta bicor estimates and P-value, provided P<0.1. The color gradient is based on -log10 P-values times sign of bicor. P-values are unadjusted.

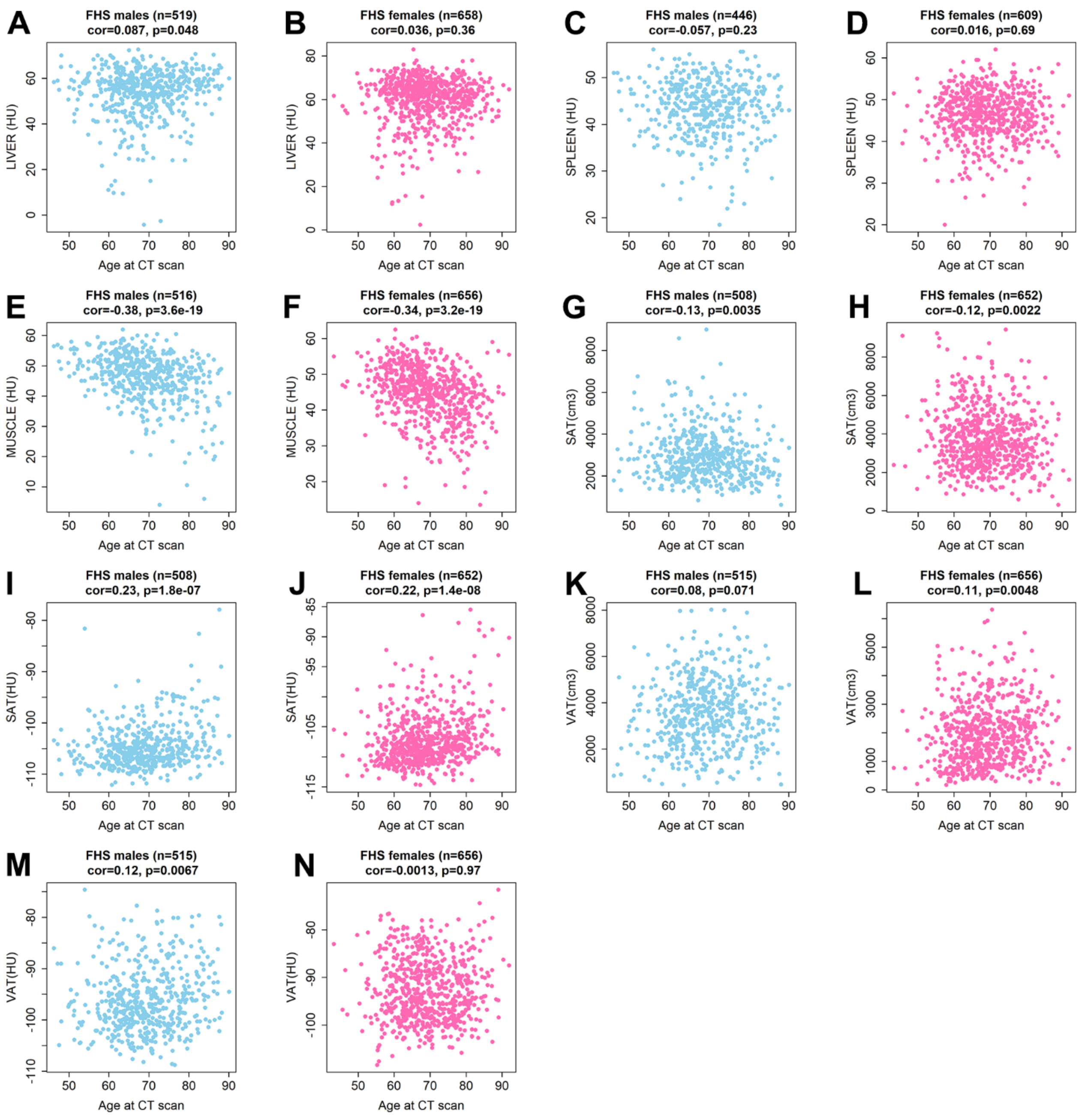


**Supplementary Figure 14. Correlation analysis of chronological age versus CT-scan fatty liver and adipose tissue density in the FHS.** We present the scatter plots of chronological age at computed tomography (CT) scan (x-axis) versus CT-scan derived measures in the FHS. The CT-scan measures included attenuation in (**A**, **B**) liver, (**C**, **D**) spleen, (**E**, **F**) paraspinal muscle, (**G**–**J**) subcutaneous adipose tissue (SAT) and (**K**–**N**) visceral adipose tissue (VAT). (**A**–**F**, **I**, **J**, **M**, **N**) are in Hounsfield (HU) unit, obtained from a linear transformation of attenuation coefficients. (**G**, **H**, **K**, **L**) are measures of volume in units of $cm^3$.

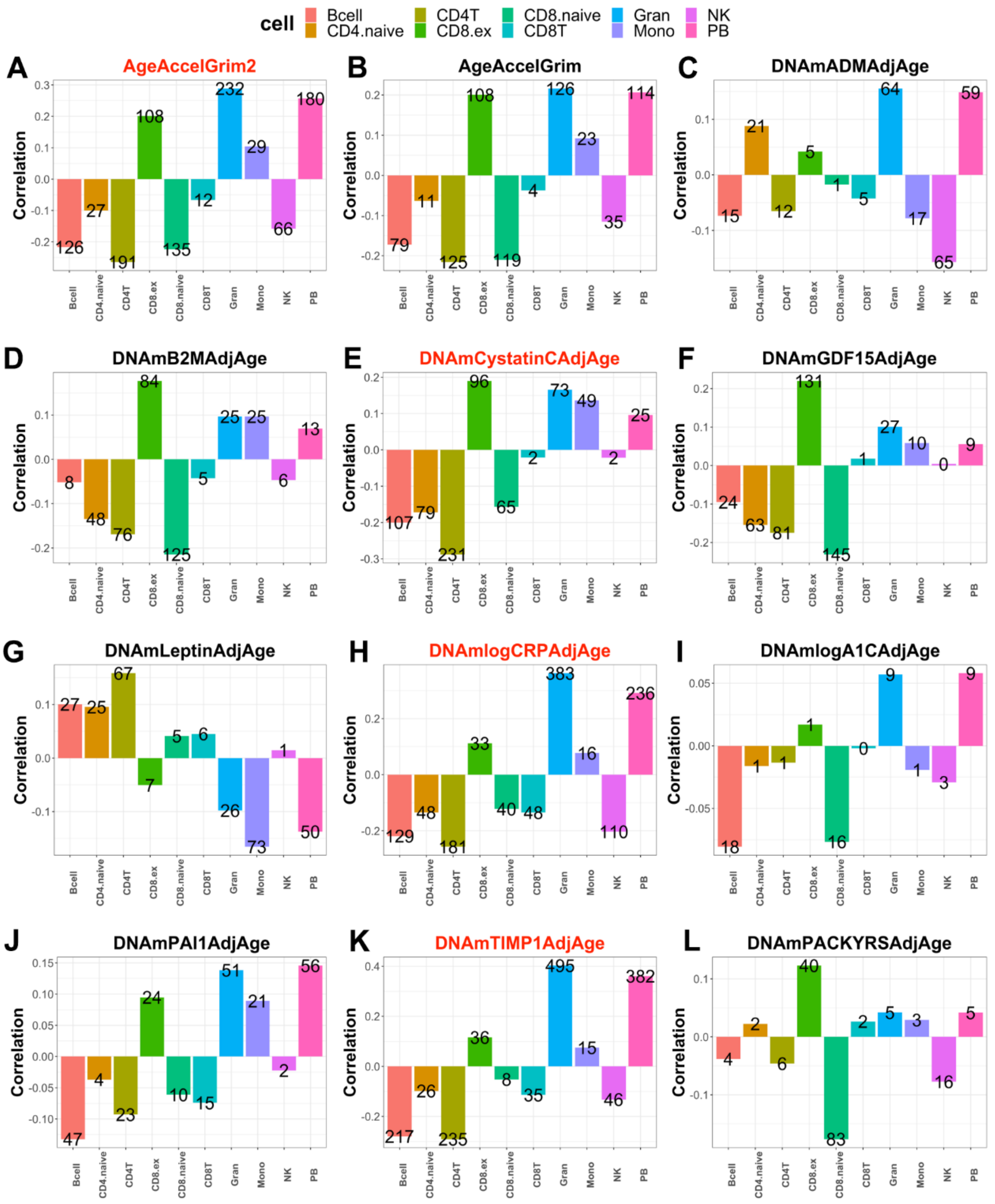


**Supplementary Figure 15. Measures of blood cell composition versus DNAm based biomarkers.** Each panel reports how the respective DNAm based biomarker (heading) relates to 10 imputed measures of blood cell counts. (**A**, **B**) display the results for AgeAccelGrim2 and AgeAccelGrim. (**C**–**L**) display the results for (age-adjusted) DNAm based surrogate markers of (**C**) adrenomedullin (ADM), (**D**) beta-2 microglobulin (B2M), (**E**) cystatin C (Cystatin C), (**F**) growth differentiation factor 15 (GDF-15), (**G**) leptin, (**H**) log scale of C reactive protein (CRP), (**I**) log scale of hemoglobin A1C, (**J**) plasminogen activation inhibitor 1 (PAI-1), (**K**) tissue inhibitor metalloproteinase 1 (TIMP-1) and (**L**) smoking pack-years (PACKYRS). The height of each bar corresponding to the statistical significance level (meta analysis p-value) of an association test between the blood cell measure and the age-adjusted DNAm biomarker. More precisely, the y-axis presents the meta analysis estimates of the Pearson correlation coefficients. The numbers displayed on top of each bar are minus logarithm (base 10) transformed meta P values. The title is marked by red if any absolute correlation >0.25. The association analysis is *not* confounded by chronological age because we used age adjusted DNAm based biomarkers. The fixed effects meta analysis was performed across the validation study sets (N=11672): FHS test, WHI BA23, JHS, InCHIANTI, BLSA, LBC21, LBC36 and NAS. Abbreviations for cell counts are listed in the following: nature killer (NK), monocyte (MONO) and granulocyte (Gran), CD8pCD28nCD45Ran (CD8.ex for exhausted cytotoxic T cells), and plasma blast (PB). The blood cell counts were imputed based on DNA methylation levels as described in [40, 42] and the Supplementary Methods section (above).

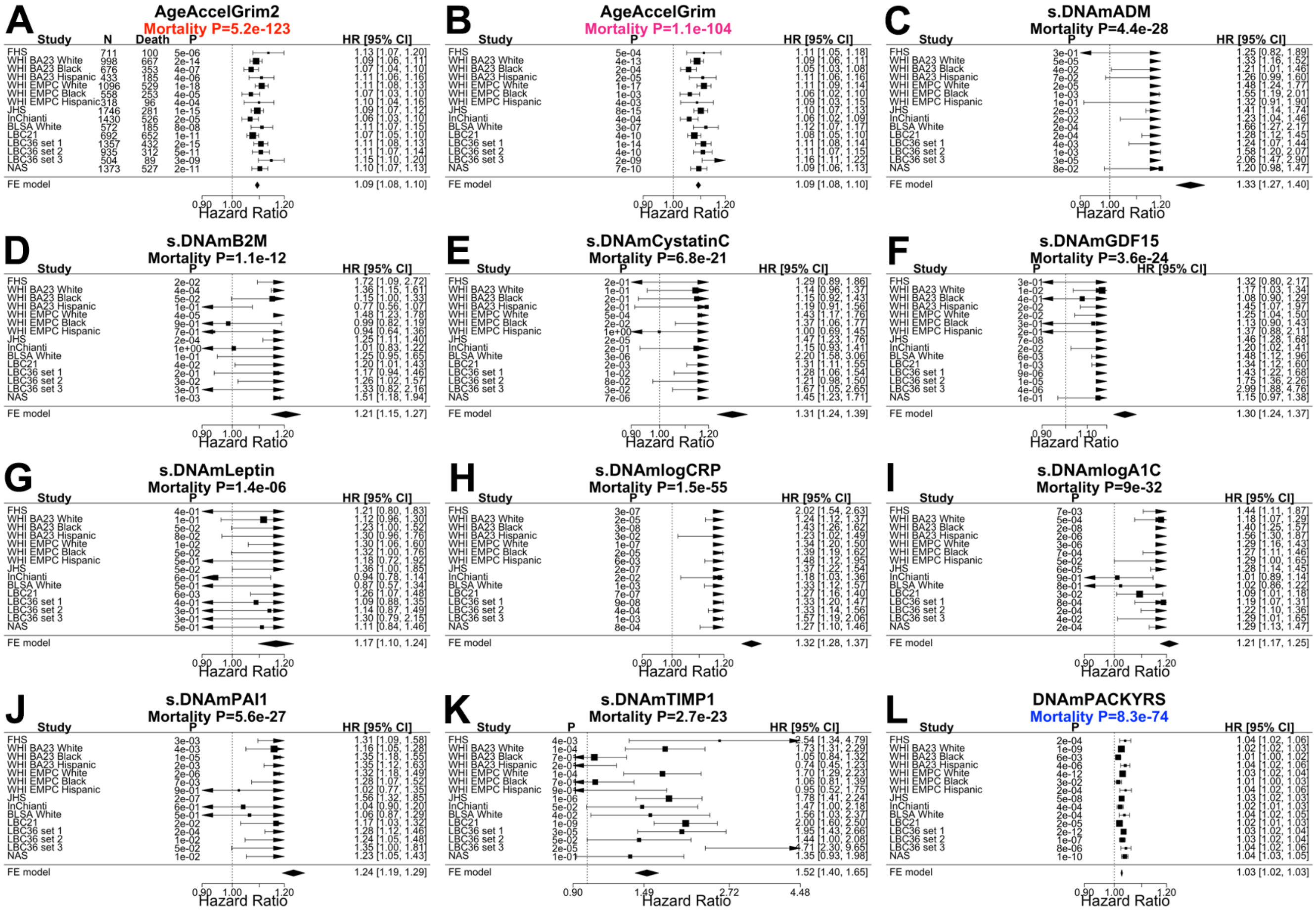

**Supplementary Figure 16. Meta analysis forest plots for predicting time-to-death adjusted for blood cell composition.** Each panel reports a meta analysis forest plot for combining hazard ratios predicting time-to-death based on a DNAm based biomarker (reported in the figure heading) across different strata formed by racial group within cohort and set within LBC36. Here we re-conducted the survival analysis as listed in Figure 2 and adjusted additional 7 imputed blood cell counts: CD8 naïve, CD8pCD28nCD45Ran, plasma blasts, CD4+ T, nature killer cells, monocytes and granulocytes. (**A**, **B**) display the results for AgeAccelGrim2 and AgeAccelGrim. Each row reports a hazard ratio (for time-to-death) and a 95% confidence interval resulting from a Cox regression model in each of 15 strata. (**C**–**L**) display the results for (age-adjusted) DNAm based surrogate markers of (**C**) adrenomedullin (ADM), (**D**) beta-2 microglobulin (B2M), (**E**) cystatin C (Cystatin C), (**F**) growth differentiation factor 15 (GDF-15), (**G**) leptin, (**H**) log scale of C reactive protein (CRP), (**I**) log scale of hemoglobin A1C, (**J**) plasminogen activation inhibitor 1 (PAI-1), (**K**) tissue inhibitor metalloproteinase 1 (TIMP-1) and (**L**) smoking pack-years (PACKYRS). The sub-title of each panel reports the meta analysis P-value. (**A**, **B**) Each hazard ratio (HR) corresponds to a one-year increase in AgeAccel. (**C**–**K**) Each hazard ratio corresponds to an increase in one-standard deviation. (**L**) Hazard ratios correspond to a one-year increase in pack-years. The most significant meta analysis P-value is marked in red (AgeAccelGrim2), followed by hot pink (AgeAccelGrim) and blue (DNAm PACKYRS), respectively.

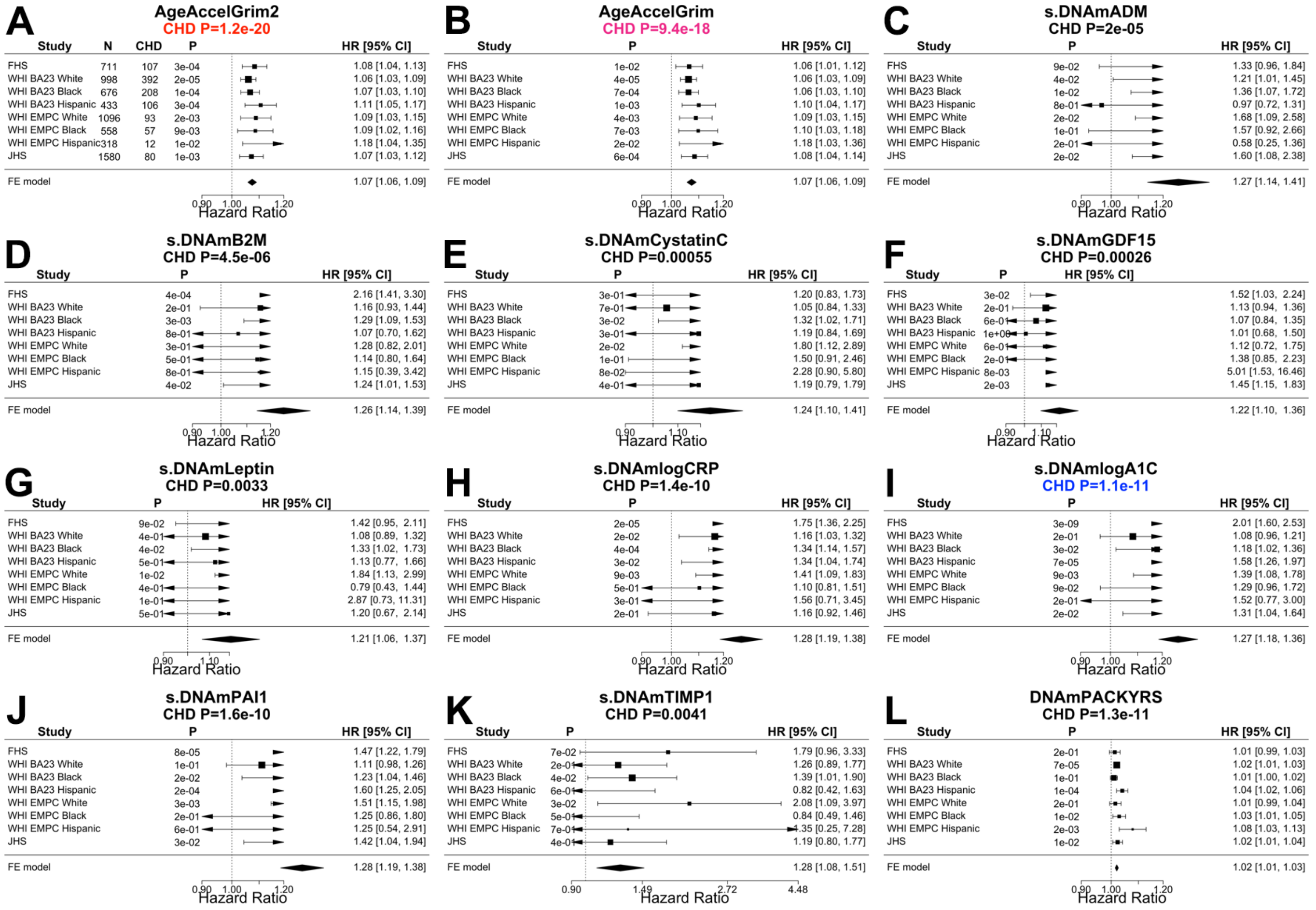

**Supplementary Figure 17. Meta analysis forest plots for predicting time-to- coronary heart disease adjusted for blood cell composition.** Each panel reports a meta analysis forest plot for combining hazard ratios predicting time-to-coronary heart disease (CHR) based on a DNAm based biomarker (reported in the figure heading) across different strata formed by racial group within cohort and set within LBC36. Here we re-conducted the survival analysis as listed in Figure 4 and adjusted additional 7 imputed blood cell counts: CD8 naïve, CD8pCD28nCD45Ran, plasma blasts, CD4+ T, natural killer cells, monocytes and granulocytes. (**A**, **B**) Results for AgeAccelGrim2 and AgeAccelGrim. Each row reports a hazard ratio (for time-to-CHD) and a 95% confidence interval resulting from a Cox regression model in each strata. (**C**–**L**) display the results for (age-adjusted) DNAm based surrogate markers of (**C**) adrenomedullin (ADM), (**D**) beta-2 microglobulin (B2M), (**E**) cystatin C (Cystatin C), (**F**) growth differentiation factor 15 (GDF-15), (**G**) leptin, (**H**) log scale of C reactive protein (CRP), (**I**) log scale of hemoglobin A1C, (**J**) plasminogen activation inhibitor 1 (PAI-1), (**K**) tissue inhibitor metalloproteinase 1 (TIMP-1) and (**L**) smoking pack-years (PACKYRS). The sub-title of each panel reports the meta analysis P-value. (**A**, **B**) Each hazard ratio (HR) corresponds to a one-year increase in AgeAccel. (**C**–**K**) Each hazard ratio corresponds to an increase in one-standard deviation. (**L**) Hazard ratios correspond to a one-year increase in pack-years. The most significant meta analysis P-value is marked in red (AgeAccelGrim2), followed by hot pink (AgeAccelGrim) and blue (DNAm logA1C), respectively.

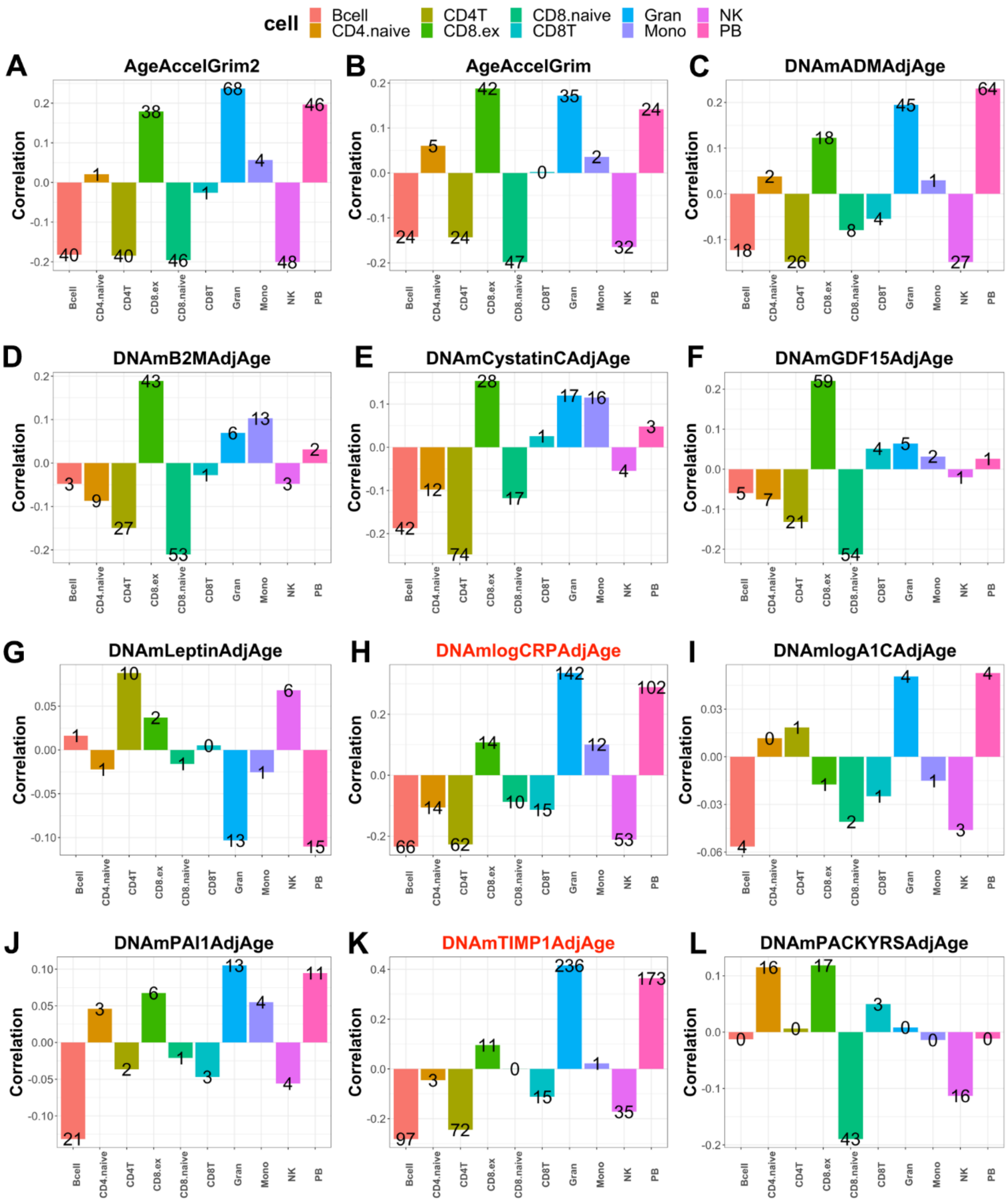


**Supplementary Figure 18. Measures of blood cell composition versus DNAm based biomarkers in males.** Each panel reports how the respective DNAm based biomarker (heading) relates to 10 imputed measures of blood cell counts. (**A**, **B**) display the results for AgeAccelGrim2 and AgeAccelGrim. (**C**–**L**) display the results for (age-adjusted) DNAm based surrogate markers of (**C**) adrenomedullin (ADM), (**D**) beta-2 microglobulin (B2M), (**E**) cystatin C (Cystatin C), (**F**) growth differentiation factor 15 (GDF-15), (**G**) leptin, (**H**) log scale of C reactive protein (CRP), (**I**) log scale of hemoglobin A1C, (**J**) plasminogen activation inhibitor 1 (PAI-1), (**K**) tissue inhibitor metalloproteinase 1 (TIMP-1) and (**L**) smoking pack-years (PACKYRS). The height of each bar corresponding to the statistical significance level (meta analysis p-value) of an association test between the blood cell measure and the age-adjusted DNAm biomarker. More precisely, the y-axis presents the meta analysis estimates of the Pearson correlation coefficients. The numbers displayed on top of each bar are minus logarithm (base 10) transformed meta P values. The title is marked by red if any absolute correlation >0.25. The association analysis is *not* confounded by chronological age because we used age adjusted DNAm based biomarkers. The fixed effects meta analysis was performed on males only across the validation study sets (N=5153): FHS test, JHS, InCHIANTI, BLSA, LBC21, LBC36 and NAS. Abbreviations for cell counts are listed in the following: nature killer (NK), monocyte (MONO) and granulocyte (Gran), CD8pCD28nCD45Ran (CD8.ex for exhausted cytotoxic T cells), and plasma blast (PB). The blood cell counts were imputed based on DNA methylation levels as described in [40, 42] and the Supplementary Methods section.

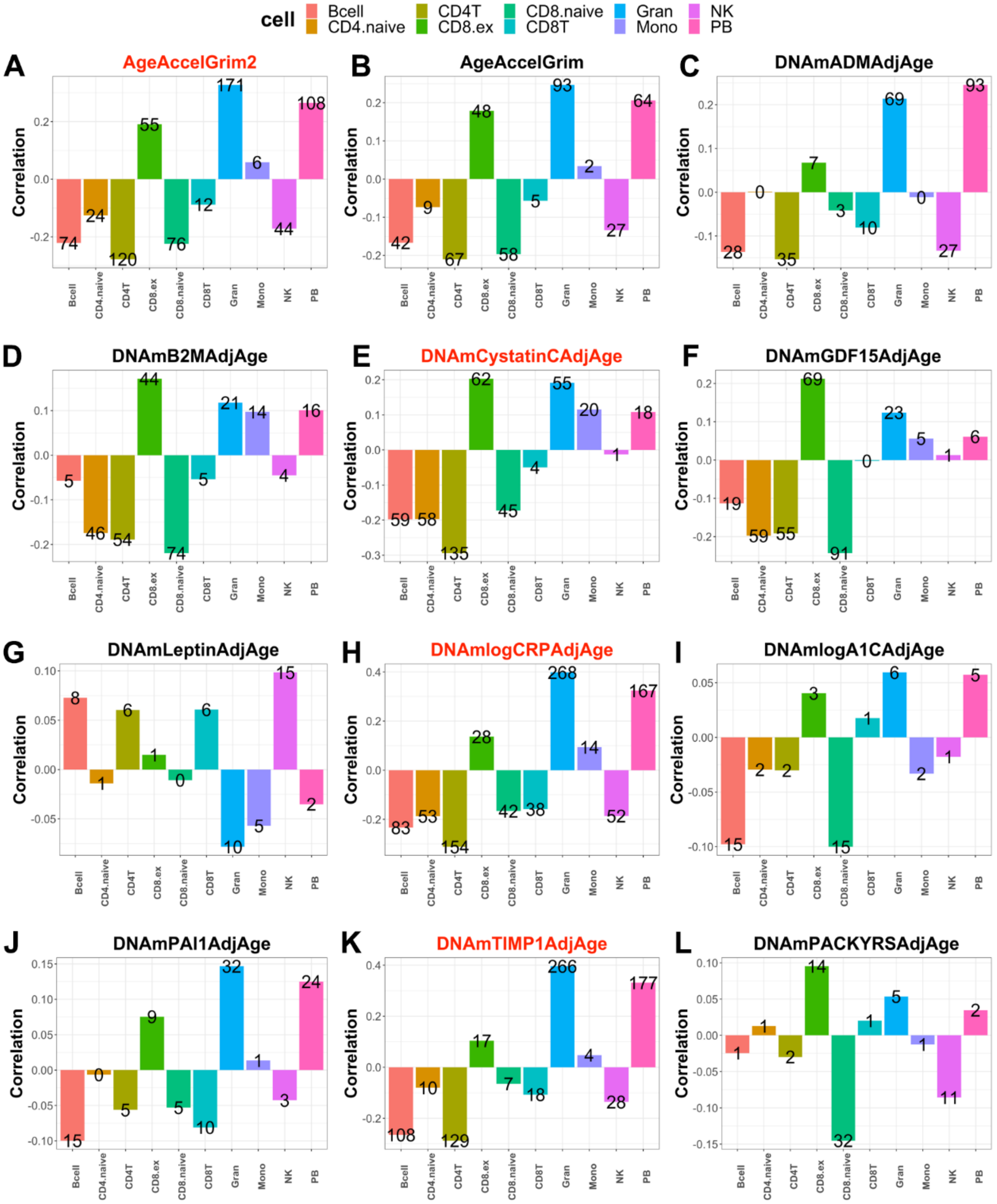


**Supplementary Figure 19. Measures of blood cell composition versus DNAm based biomarkers in females.** Each panel reports how the respective DNAm based biomarker (heading) relates to 10 imputed measures of blood cell counts. (**A**, **B**) display the results for AgeAccelGrim2 and AgeAccelGrim. (**C**–**L**) display the results for (age-adjusted) DNAm based surrogate markers of (**C**) adrenomedullin (ADM), (**D**) beta-2 microglobulin (B2M), (**E**) cystatin C (Cystatin C), (**F**) growth differentiation factor 15 (GDF-15), (**G**) leptin, (**H**) log scale of C reactive protein (CRP), (**I**) log scale of hemoglobin A1C, (**J**) plasminogen activation inhibitor 1 (PAI-1), (**K**) tissue inhibitor metalloproteinase 1 (TIMP-1) and (**L**) smoking pack-years (PACKYRS). The height of each bar corresponding to the statistical significance level (meta analysis p-value) of an association test between the blood cell measure and the age-adjusted DNAm biomarker. More precisely, the y-axis presents the meta analysis estimates of the Pearson correlation coefficients. The numbers displayed on top of each bar are minus logarithm (base 10) transformed meta P values. The title is marked by red if any absolute correlation >0.25. The association analysis is *not* confounded by chronological age because we used age adjusted DNAm based biomarkers. The fixed effects meta analysis was performed on females only across the validation study sets (N=6519): FHS test, WHI BA23, JHS, InCHIANTI, BLSA, LBC21, and LBC36. Abbreviations for cell counts are listed in the following: nature killer (NK), monocyte (MONO) and granulocyte (Gran), CD8pCD28nCD45Ran (CD8.ex for exhausted cytotoxic T cells), and plasma blast (PB). The blood cell counts were imputed based on DNA methylation levels as described in [40, 42] and the Supplementary Methods section.

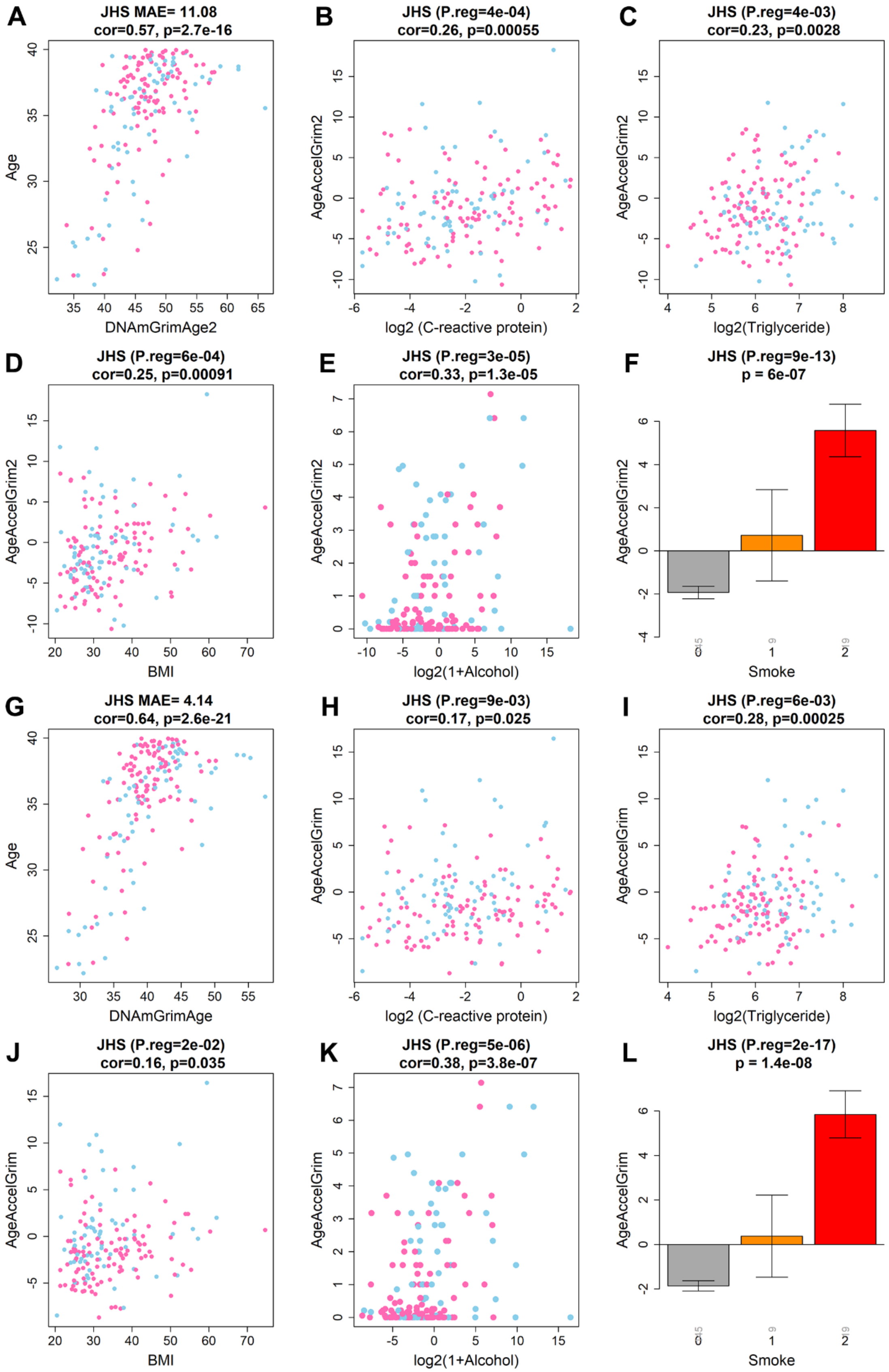


**Supplementary Figure 20. Applying GrimAge clocks on young people in Jackson Heart Study.** We evaluated our GrimAge clocks on the young population (age <40, n=173 with 62% females) in Jackson Heart Study (JHS) cohort. (**A**–**F**) present the assessments for (**A**)

correlation between chronological age and DNAmGrimAge2 and (**B**–**F**) associations between AgeAccelGrim2 with log2 C-reactive protein, log2 triglyceride, body mass index (BMI), log2 (alcohol assumption +1), and smoking status (0=never, 1=past, and 2=current). For (**B**–**E**) we report the P value (P.reg) from linear regression analysis, Pearson correlation estimate and P value. (**F**) reports the P value (P.reg) from linear regression analysis and the p-value of a non-parametric group comparison test (Kruskal-Wallis). The y-axis of the bar plot depicts the mean and one standard error. For (**B**–**D**) linear regression analysis was performed for outcome measures (as dependent variable) on AgeAccelGrim2 (as independent variable), adjusted for chronological age and gender. For (**E**, **F**) linear regression analysis was performed for AgeAccelGrim2 (as dependent variable) on life style variable (as independent variable), adjusted for chronological age and gender. The number under each bar presents number of individuals at each racial group. The lower (**G**–**L**) present the same assessments for DNAmGrimAge and AgeAccelGrim.

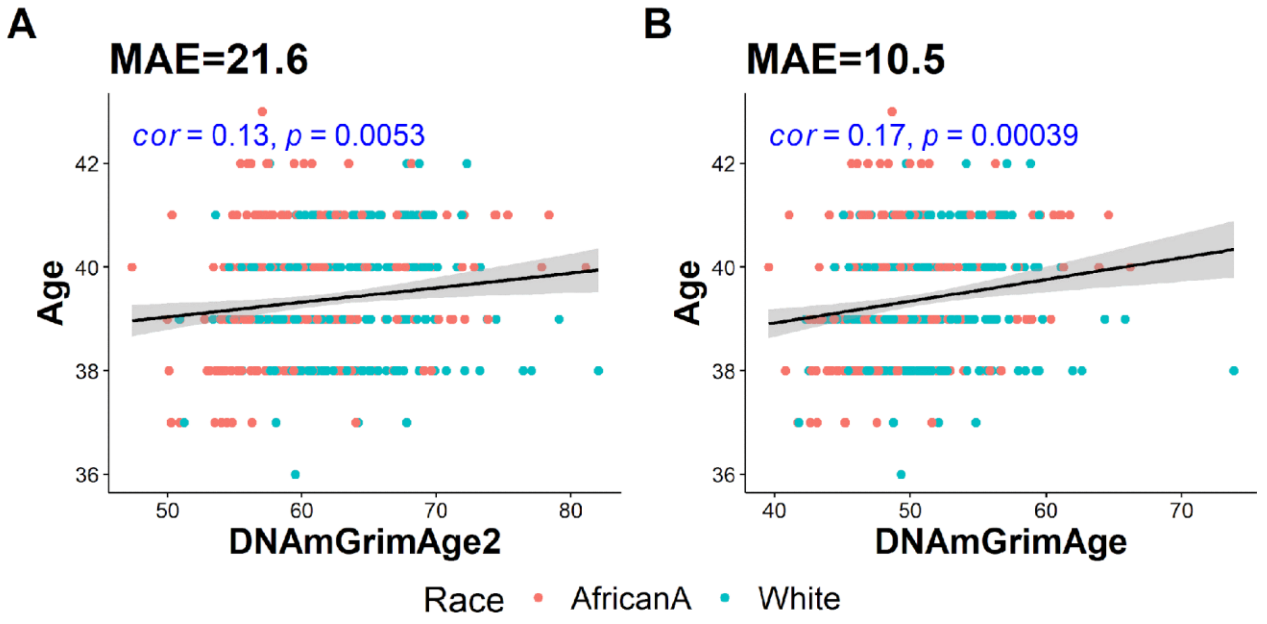


**Supplementary Figure 21. Correlation analysis of DNAmGrimAge(2) versus chronological age in NGHS mothers .** We present the scatter plots of chronological age versus (**A**) DNAmGrimAge2, and (**B**) DNAmGrimAge estimated in methylation array profiled in saliva samples from 432 mothers in NGHS. Each dot represents an individual sample colored based on ethnic/racial groups: White (n=218) and African American (n=214). The title of each panel repot the median of absolute error in units of years. The Pearson correlation coefficient (cor) and a corresponding correlation test p-value are reported at each panel.

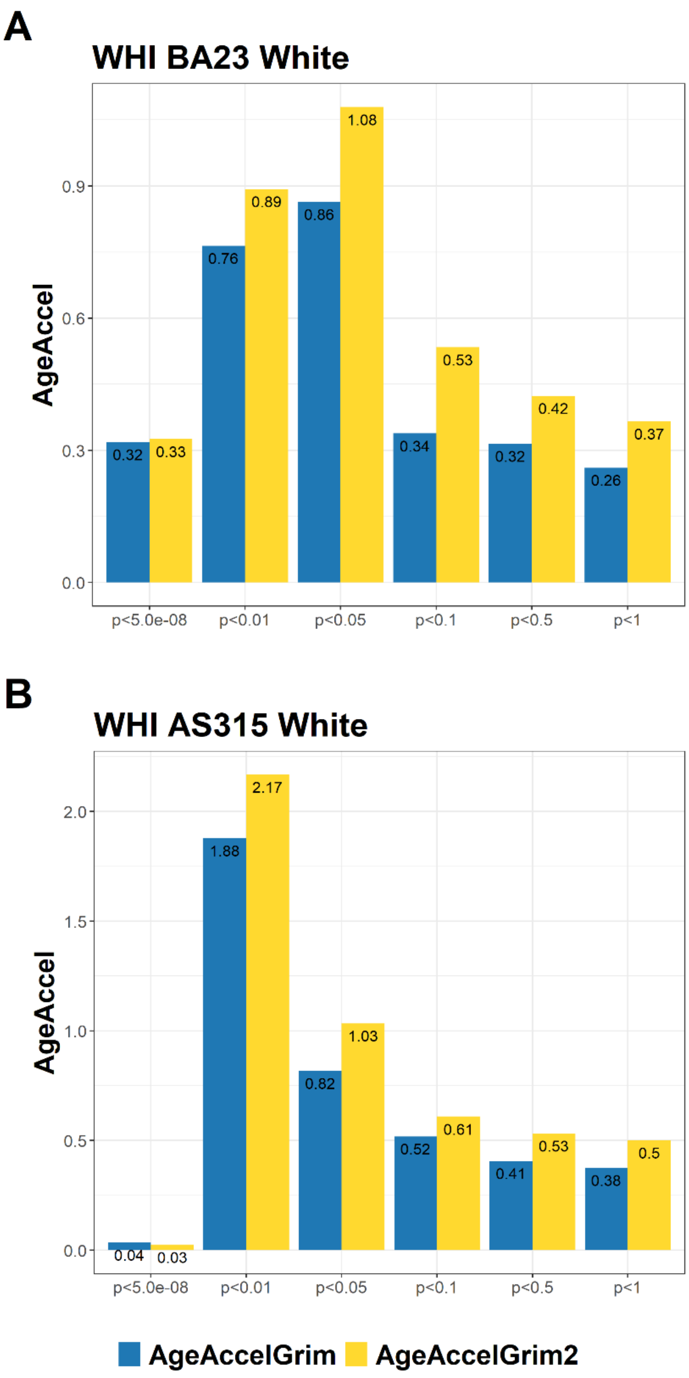


**Supplementary Figure 22. Polygenic risk score analysis using WHI cohort.** Polygenic risk score analysis (PRS) was applied to the women with European ancestry from (**A**) WHI BA23 and (**B**) EMPC cohorts, respectively. We calculate the proportion of the variation in AgeAccelGrim/AgeAccelGrim2 that can be explained by PRS at SNP P values thresholds set at <5.0e-08,0.01,0.05,0.1,0.5, and 1. The y-axis displays the proportion in percentage (%) and the x-axis displays different thresholds of P values. The proportions (in percentage %) are listed on the top of each bar.

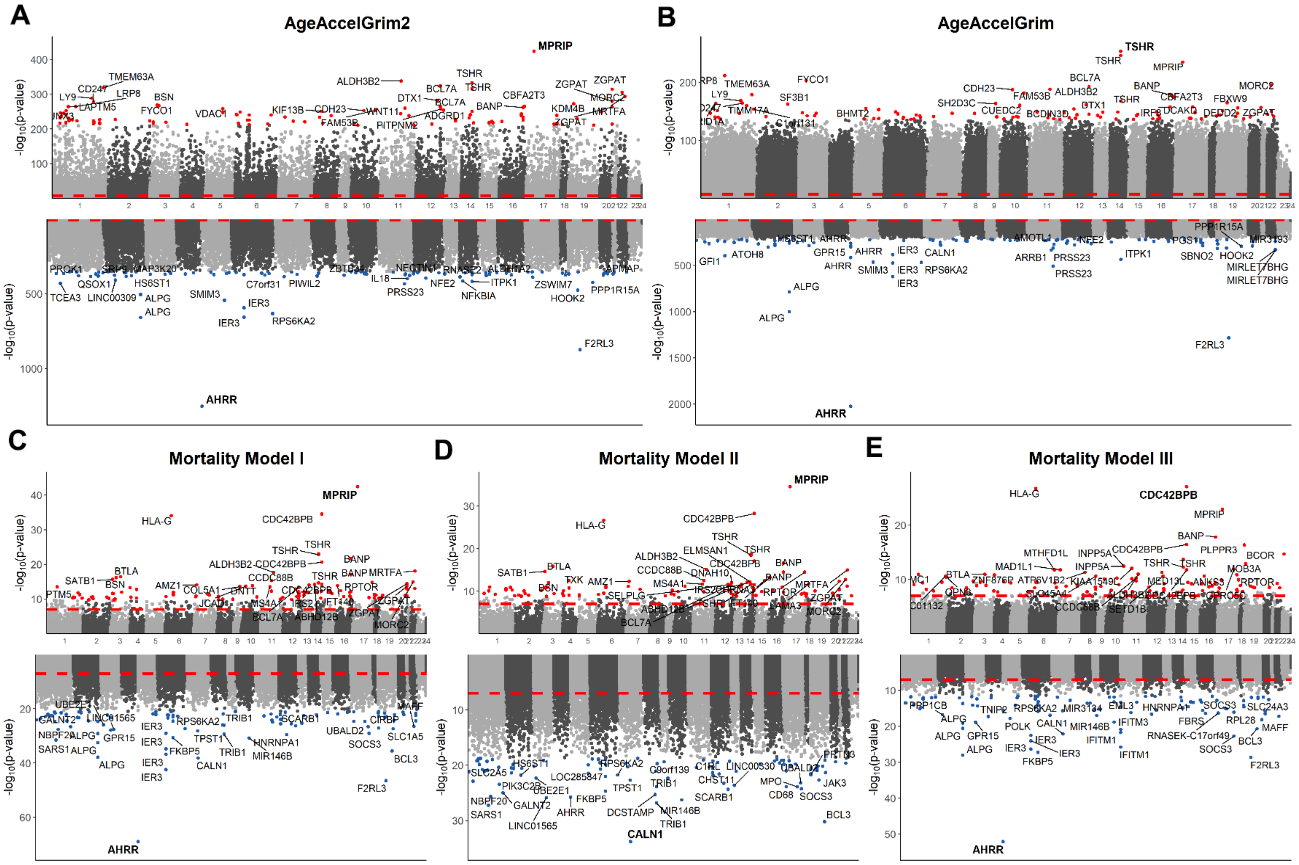


**Supplementary Figure 23. Epigenome-wide association study (EWAS) for mortality related traits.** Meta-analysis p-value (-log base 10 transformed) versus chromosomal location (x-axis) according to human genome assembly 19 (hg19) in (**A**), linear regression of AgeAccelGrim2, (**B**) linear regression of AgeAccelGrim, (**C**) Model I: Cox regression of time-to-death adjusted for age, gender, and batch effect (**D**) Model II: Cox regression of time-to-death adjusted for age, gender, batch effect, and smoking packyears (or smoking status) and (**E**) Model III : Cox regression of time-to-death adjusted for age, gender, batch effect, and 7 imputed blood cell counts: CD8 naïve, CD8pCD28nCD45Ran, plasma blasts, CD4+ T, nature killer cells, monocytes and granulocytes. In (**A**, **B**) age acceleration of GrimAge clocks are increasing/decreasing with the methylation levels of the CpGs in the top/bottom panes. In (**C**, **D**) the hazard ratios of time-to-death are increasing/decreasing with the methylation levels of the CpGs in the top/bottom panes. Red dashed horizontal lines denote P at 1.0E-07. Gene names are annotated for the top 30 CpGs with positive and negative associations, respectively. CpGs are labeled by adjacent genes with the most significant one marked in bold in both top and bottom panes.

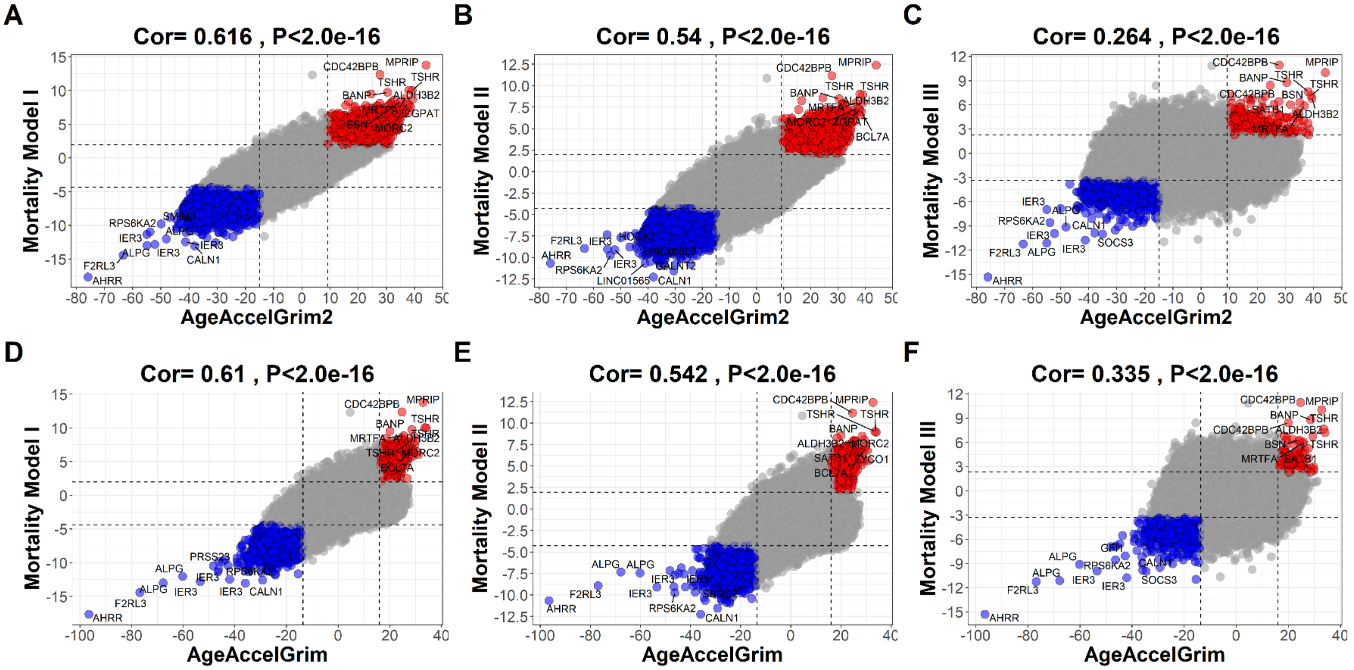


**Supplementary Figure 24. Correlation between EWAS of age acceleration of GrimAge clocks and EWAS of time-to-death.** Following the meta-analysis displayed in Supplementary Figure 23, the top panels display the meta Z scores from the EWAS of AgeAccelGrim2 on x-axis versus the meta Z scores from the EWAS of time-to-death based on Model I (**A**), Model II (**B**) and Model III (**C**) on y-axis. The bottom panels display the meta Z scores from the EWAS of AgeAccelGrim on x-axis versus the meta Z scores from the EWAS of time-to-death based on Model I (**D**), Model II (**E**) and Model III (**F**) on y-axis. Each dot corresponds to a CpG. Labels are provided for the top 10 CpGs in quadrant I and III, respectively, according to the product of Z scores in x and y axis. The title lists the Pearson correlation coefficient and corresponding nominal (unadjusted) two-sided correlation test P-value.

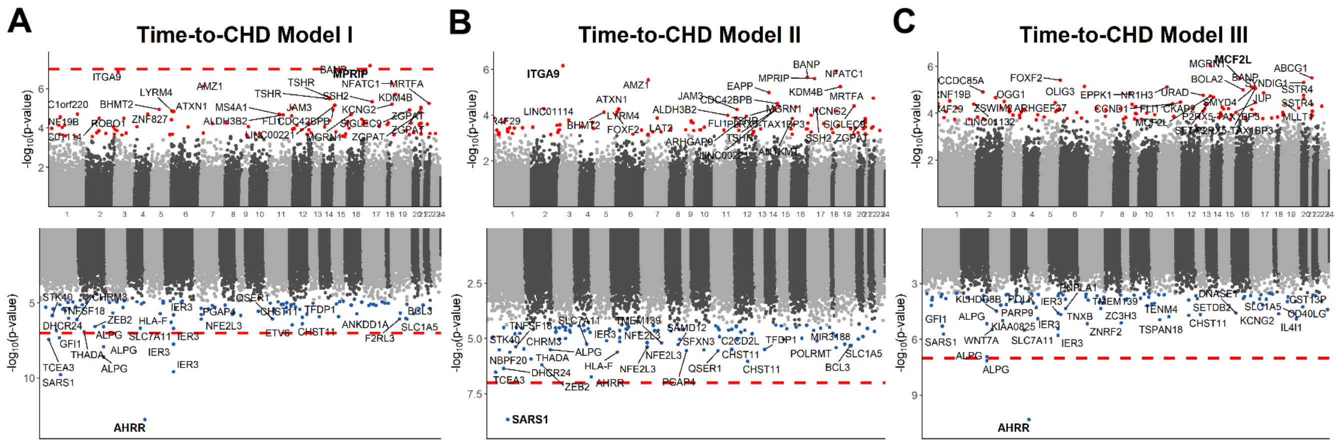


**Supplementary Figure 25. Epigenome-wide association study (EWAS) for time-to-coronary heart disease.** Meta-analysis p-value (-log base 10 transformed) versus chromosomal location (x-axis) according to human genome assembly 19 (hg19). (**A**) Model I: Cox regression of time-to-coronary heart disease (CHD) adjusted for age, gender, and batch effect (**B**) Model II: Cox regression of time-to-CHD adjusted for age, gender, batch effect, and smoking packyears (or smoking status) and (**C**) Model III : Cox regression of time-to-CHD adjusted for age, gender, batch effect, and 7 imputed blood cell counts: CD8 naïve, CD8pCD28nCD45Ran, plasma blasts, CD4+ T, nature killer cells, monocytes and granulocytes. At each panel, the hazard ratios of time-to-CHD are increasing/decreasing with the methylation levels of the CpGs in the top/bottom panes. Red dashed horizontal lines denote P at 1.0E-07. Gene names are annotated for the top 30 CpGs with positive and negative associations, respectively. CpGs are labeled by adjacent genes with the most significant one marked in bold in both top and bottom.

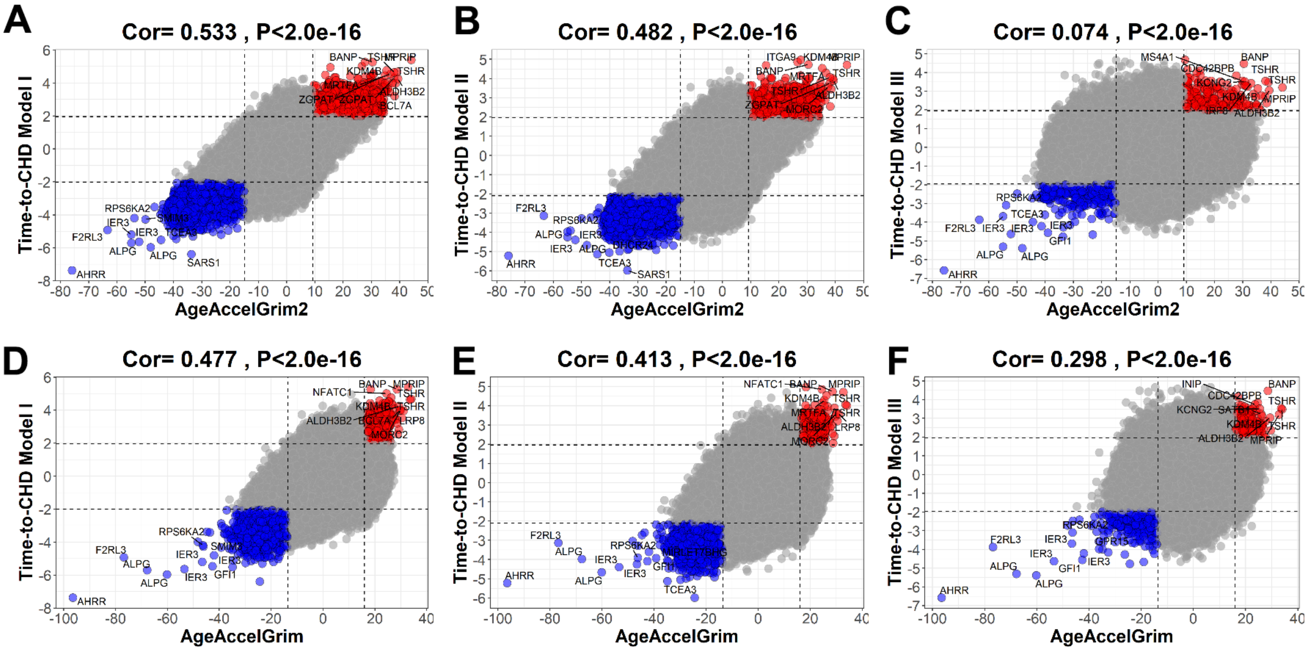


**Supplementary Figure 26. Correlation between EWAS of age acceleration of GrimAge clocks and EWAS of time-to-coronary heart disease.** Following the meta-analysis displayed in Supplementary Figures 23, 25, the top panels display the meta Z scores from the EWAS of AgeAccelGrim2 on x-axis versus the meta Z scores from the EWAS of time-to-coronary heart disease (CHD) based on Model I (**A**), Model II (**B**) and Model III (**C**) on y-axis. The bottom panels display the meta Z scores from the EWAS of AgeAccelGrim on x-axis versus the meta Z scores from the EWAS of time-to-CHD based on Model I (**D**), Model II (**E**) and Model III (**F**) on y-axis. Each dot corresponds to a CpG. Labels are provided for the top 10 CpGs in quadrant I and III, respectively, according to the product of Z scores in x and y axis. The title lists the Pearson correlation coefficient and corresponding nominal (unadjusted) two-sided correlation test P-value.
