# Supplementary material for: DNA methylation GrimAge version 2
Source: Aging (Albany NY). 2022 Dec 14;14(23):9484–549. doi: 10.18632/aging.204434 (PMC9792204; doi:10.18632/aging.204434)
Supplement: Supplementary Tables 1.1-2.1 [file aging-14-204434-s003.pdf]

SUPPLEMENTARY TABLES

Supplementary Table 1.1. GrimAge2 stage 1: DNAm-based surrogate biomarkers of plasma proteins and smoking pack-years.

Variable	Num CpGs	correlation with biomarker in training data	correlation with age in training data	correlation with biomarker in test data	correlation with age in test data	Exam
adm	186	0.653693573174295	0.625525905864977	0.381687444097942	0.639017395854806	exam 7
B2M	91	0.617158399278239	0.825073799472286	0.426041190563136	0.848465319002703	exam 7
cd56	607	0.86375831161774	0.172605242662232	0.361637489604755	0.170522481815106	exam 7
Cystatin_C	87	0.580806669506421	0.812098347065102	0.392285471797308	0.827175556961344	exam 7
EFEMP1	57	0.589686685159107	0.719151039155843	0.412150311207909	0.872303871874163	exam 7
GDF_15	137	0.737468260449462	0.71492538275353	0.534804728797616	0.806519824631994	exam 7
leptin	187	0.681181180674115	0.0581128431496463	0.352344410014838	0.0514309778202585	exam 7
log.A1C	86	0.525131694119941	0.31582295531732	0.339957448360379	0.26677461707641	exam 8
log.CRP	132	0.569330798879011	0.272903575140915	0.476839364307574	0.261805340722653	exam 8
PACKYRS	172	0.785	0.17	0.66	0.13	exam 8
pai_1	211	0.691865627259714	0.190154508514923	0.362625398311644	0.16187195108164	exam 7
TIMP_1	42	0.431107933469501	0.917716575776463	0.350384409802195	0.898106800656296	exam 7

Supplementary Table 1.2. GrimAge2: Distribution of DNAm proteins based on FHS training dataset.

Variable	mean	sd
DNAmADM	337.443763330646	26.8386567435564
DNAmB2M	1633051.85941816	166877.416872265
DNAmCystatinC	591129.33954085	41113.1707392004
DNAmGDF15	678.704154283819	175.497882136521
DNAmLeptin	8360.49439150999	4368.05918344219
DNAmlogA1C	1.73769184385078	0.03209281978435
DNAmlogCRP	0.447021928835783	0.439141130146429
DNAmPAI1	19804.6891037806	3325.68848188053
DNAmTIMP1	34348.2946807127	1548.59018754715

Supplementary Table 2.1. Description of variable availability for Diet, clinically relevant measurements, and life style factors.

Order	Category	Var	NumData	n	FHS	WHI	JHS	InCHIANTI	BLSA	LBC21	LBC36	NAS
1	Diet	log2(Total energy)	2	4221		x					x	
2	Diet	Carbohydrate	2	4222		x					x	
3	Diet	Protein	2	4221		x					x	
4	Diet	Fat	2	4221		x					x	
5	Diet	log2(1+Red meat)	3	4873	x	x					x	
6	Diet	log2(1+Poultry)	3	4832	x	x					x	
7	Diet	log2(1+Fish)	3	4873	x	x					x	
8	Diet	log2(1+Dairy)	3	4868	x	x					x	
9	Diet	log2(1+Whole grains)	2	4108	x	x						
10	Diet	log2(1+Nuts)	1	3463		x						
11	Diet	log2(Fruits)	3	4864	x	x					x	
12	Diet	log2(Vegetables)	3	4864	x	x					x	
14	Diet	log(OMEGA3)	1	643	x							
15	Diet	log(VitaminA)	1	651	x							
16	Diet	log(VitaminC)	2	1409	x						x	
17	Diet	log(VitaminB6)	2	1407	x						x	
18	Diet	log(VitaminE)	2	1397	x						x	
19	Diet	log(Selenium)	2	1401	x						x	
20	Diet	log(Iron)	1	633	x							
21	Diet	log(Zinc)	2	1404	x						x	
22	Diet	log(Calcium)	2	1407	x						x	
23	Diet	log(FolicAcid)	2	1407	x						x	
24	Diet	log(VitaminD)	2	1397	x						x	
25	Diet	log(Copper)	1	643	x							
26	Diet	log(BrewYeast)	1	643	x							
27	Diet	log(BetaCaroteneSup)	1	645	x							
28	Diet	log(Magnesium)	1	641	x							
29	Dietary Biomarkers	Retinol	1	2053		x						
30	Dietary Biomarkers	Mean carotenoids	1	2052		x						
31	Dietary Biomarkers	Lycopene	1	2053		x						
32	Dietary Biomarkers	log2(alpha-Carotene)	1	2053		x						
33	Dietary Biomarkers	log2(beta-Carotene)	1	2052		x						
34	Dietary Biomarkers	log2(Lutein+Zeaxanthin)	1	2053		x						
35	Dietary Biomarkers	log2(beta-Cryptoxanthin)	1	2053		x						
36	Dietary Biomarkers	log2(alpha-Tocopherol)	1	2053		x						
37	Dietary Biomarkers	log2(gamma-Tocopherol)	1	2053		x						
38	Measurements	log(A1C)	1	711	x							
39	Measurements	log2(C-reactive protein)	8	11281	x	x	x	x	x	x	x	x
40	Measurements	log2(Insulin)	3	5912		x		x				x
41	Measurements	log2(Glucose)	5	7392	x	x		x	x			x
42	Measurements	log2(Triglyceride)	6	9847	x	x	x	x	x			x
43	Measurements	Total cholesterol	8	13002	x	x	x	x	x	x	x	x
44	Measurements	LDL cholesterol	4	7688		x	x	x	x			
45	Measurements	HDL cholesterol	6	9844	x	x	x	x	x			x
46	Measurements	log2(Creatinine)	3	4770	x	x		x				

47	Measurements	log2(IL6)	3	3185				x	x			x
48	Measurements	log2(TNFA)	3	2621				x	x			x
49	Measurements	log2(Urine Creatinine)	3	1793	x		x	x				
50	Measurements	FEV1	5	5836	x				x	x	x	x
52	Measurements	Systolic blood pressure	6	11980	x	x	x	x			x	x
53	Measurements	Diastolic blood pressure	6	11980	x	x	x	x			x	x
54	Measurements	log2(Waist / hip ratio)	3	5887	x	x						x
55	Measurements	BMI	8	13420	x	x	x	x	x	x	x	x
56	Measurements	MMSE	6	7017	x	x		x	x	x	x	
57	Measurements	Telomere length	3	2193	x	x						x
58	Life style	Education	8	13312	x	x	x	x	x	x	x	x
59	Life style	Income	3	6687		x	x					x
60	Life style	Hand grip	5	5962	x			x	x	x	x	
61	Life style	log2(1+Exercise)	4	7278		x	x		x			x
62	Life style	Current smoker	6	10247	x	x			x	x	x	x
63	Life style	log2(1+Alcohol)	5	8050	x	x	x	x			x	
