# Supplementary material for: DNA methylation GrimAge version 2
Source: Aging (Albany NY). 2022 Dec 14;14(23):9484–549. doi: 10.18632/aging.204434 (PMC9792204; doi:10.18632/aging.204434)
Supplement: Supplementary Tables 3.1-3.5 [file aging-14-204434-s016.pdf]

Supplementary Table 3.1. Multivariate regression analysis of AgeAccelGrim2 on CT-scan derived fatty liver and adipose tissue density in FHS.

Model	Y	X	Beta	SE	P
I	AgeAccelGrim2	LIVER	-0.0725552450066406	0.0157640950628678	0.0000052462134978876
		SPLEEN	-0.0641606466770295	0.0314759794731426	0.0420133477051177
		MUSCLE	-0.0299140728046785	0.0217670903459476	0.1699445683868
		Female	-2.05039351096843	0.291933038291549	6.7760496877205E-12
		BMI	0.0318418769471347	0.0376401403591607	0.39796528544538
		Age at CT scan	-0.00992439979265223	0.0185602226136875	0.593075731831915
II	AgeAccelGrim2	SAT_CM3	0.0001175063321704	0.000168522663672719	0.485902386408158
		VAT_CM3	0.000648438884697252	0.000143475978795086	0.0000074714159508801
		Female	-1.79179445483356	0.377885807483643	2.65222492382836E-06
		BMI	0.0423208062396213	0.0516700617304267	0.413078749384451
		Age at CT scan	-0.0137130451222446	0.0166979368071955	0.411835229253499
III	AgeAccelGrim2	SAT_CM3	0.000213266609903971	0.000185658369273936	0.251137868322493
		SAT_HU	0.0518858581680036	0.0496768333623689	0.296691687990567
		VAT_CM3	0.000696277354148539	0.000225770022520319	0.00213665798620851
		VAT_HU	-0.00215892686800074	0.0479969355962227	0.964137881257326
		Female	-1.66540746493659	0.393922098195233	0.000027303482094446
		BMI	0.026757674678966	0.053215234920054	0.615276396289478
		Age at CT scan	-0.0180851191986976	0.0171297109908854	0.291498115855943
IV	AgeAccelGrim2	LIVER	-0.0626818541708387	0.0170574563176948	0.00026328300052734
		SPLEEN	-0.054951643047811	0.0333075558382417	0.0995944208257338
		MUSCLE	-0.0326249567509138	0.022474793765515	0.147220950242196
		SAT_CM3	-0.0000979759784997684	0.000188785005675917	0.60399856899
		VAT_CM3	0.000374339356189572	0.00016747464759595	0.0258363385051958
		Female	-1.46955858341664	0.423578131683179	0.000565817215577898
		BMI	0.0104187753954827	0.0559296045453909	0.852296337315191
		Age at CT scan	-0.0232044991761183	0.0190935020988578	0.224810675512135

Supplementary Table 3.2. Multivariate regression analysis of AgeAccelGrim on CT-scan derived fatty liver and adipose tissue density in FHS.

Model	Y	X	Beta	SE	P
I	AgeAccelGrim	LIVER	-0.0546693939781844	0.0142894416560176	0.000146089526183766
		SPLEEN	-0.0730238668554368	0.0285204177186902	0.0107352358788065
		MUSCLE	-0.0254844566773724	0.0197229816192019	0.196887362219345
		Female	-2.68970443896693	0.26432393954252	2.61572412382749E-22
		BMI	0.0105132397941722	0.0341221556735657	0.758124605518281
		Age at CT scan	-0.00651440056132528	0.0168534212592124	0.699259606048942
II	AgeAccelGrim	SAT_CM3	0.000157670637968458	0.000153593630455081	0.305049536220618
		VAT_CM3	0.000498830492411192	0.000130756509830738	0.00015034122401784
		Female	-2.58845270816718	0.344355524447278	2.0572998267966E-13
		BMI	0.0229333865618026	0.0470891168061544	0.626421646578195
		Age at CT scan	-0.00818237393770103	0.0152280190294081	0.591243652428715
III	AgeAccelGrim	SAT_CM3	0.000220531127826131	0.00016921748252493	0.192994953803833
		SAT_HU	0.021640035705448	0.0452770463366346	0.632862767943247
		VAT_CM3	0.000635656503675089	0.000205758533910126	0.00209935350529376
		VAT_HU	0.0272304577727766	0.0437427061130195	0.533841472589256
		Female	-2.471149627248	0.358947669020785	1.47274397318319E-11
		BMI	0.0128250099228703	0.0484980890476067	0.791529375255242
		Age at CT scan	-0.0120919656881404	0.0156250044919964	0.439304797792829
IV	AgeAccelGrim	LIVER	-0.0475308664009919	0.0154590687712233	0.00222060338431433
		SPLEEN	-0.0655966151283918	0.0301814115886675	0.0302088092527874
		MUSCLE	-0.0288585724605927	0.0203601148345682	0.156974546502247
		SAT_CM3	-0.0000645277569592293	0.000171118131252568	0.706260014989643
		VAT_CM3	0.000267511621204019	0.000151750496110608	0.0785266023805075
		Female	-2.27613778068489	0.383729334000118	5.53838746676851E-09
		BMI	-0.00329955058654534	0.0506937234630215	0.948129478492866
		Age at CT scan	-0.0180955318334558	0.0173395230458529	0.297164756461088

Supplementary Table 3.3. Multivariate regression analysis of DNAmPAI1AdjAge on CT-scan derived fatty liver and adipose tissue density in FHS.

Model	Y	X	Beta	SE	P
I	DNAmPAI1AdjAge	LIVER	-93.1160498683119	11.1566433936582	6.34802166163233E-16
		SPLEEN	-9.46221758578595	22.3749459732659	0.672547175092448
		MUSCLE	1.50197350069059	15.4690268717859	0.922687791190797
		Female	-1548.14293098737	209.468920594888	5.83551954372997E-13
		BMI9	58.241260706491	26.6190276309504	0.0291154380846502
		Age at CT scan	5.85774251435321	12.8861722223888	0.649603394362432
II	DNAmPAI1AdjAge	SAT_CM3	0.278474449545414	0.11761948706386	0.0182212234117412
		VAT_CM3	0.764719539974853	0.100362509401461	9.99534455778813E-14
		Female	-1185.68188545405	265.106918280431	9.25250195939522E-06
		BMI9	-18.1112674638562	36.1673090704662	0.616721540615711
		Age at CT scan	-12.6372328432492	11.4360903315457	0.269589868759395
III	DNAmPAI1AdjAge	SAT_CM3	0.348479480147811	0.129430579607252	0.00729296406555991
		SAT_HU	65.0792546734262	34.6363839191448	0.0607413584402037
		VAT_CM3	0.569510500269957	0.157782337342776	0.000332578216044617
		VAT_HU	-64.1739654035311	33.535306514281	0.0561461426188171
		Female	-1171.47467020196	276.165889678789	0.0000256828920671473
		BMI9	-29.7270181063069	37.1901222248041	0.424419520489371
		Age at CT scan	-13.6131465698224	11.7440482420606	0.246857321082752
IV	DNAmPAI1AdjAge	LIVER	-73.0814526252046	11.8486649492233	1.40977703397388E-09
		SPLEEN	13.2395901044582	23.1746825945454	0.568051069566604
		MUSCLE	-1.16768020363668	15.668413693673	0.940622293606083
		SAT_CM3	0.305262987704478	0.130871772484245	0.0200606974925636
		VAT_CM3	0.631192083678622	0.116514425102993	9.34151169101383E-08
		Female	-1063.2913100898	295.19219180069	0.000346655934718117
		BMI9	-59.0176129450417	38.8452015553271	0.129306031677585
		Age at CT scan	-7.74139415834141	13.0351867068401	0.55285274549796

Supplementary Table 3.4. Multivariate regression analysis of DNAmlogCRPAdjAge on CT-scan derived fatty liver and adipose tissue density in FHS.

Model	Y	X	Beta	SE	P
I	DNAmlogCRPAdjAge	LIVER	-0.00780143813357888	0.00135865529996456	1.58923063186677E-08
		SPLEEN	0.00184718877057261	0.00272140514411132	0.497588935065418
		MUSCLE	-0.00110300818926791	0.00188190714149873	0.558053651258652
		Female	0.203304682834499	0.0254062027717669	7.96441025158663E-15
		BMI	0.0134030663180608	0.00324206486118264	0.0000415110836793144
		Age at CT scan	-0.000982251385415205	0.00157747554513246	0.533771363011607
II	DNAmlogCRPAdjAge	SAT_CM3	0.0000200597481523092	0.0000145217529854608	0.167683781857688
		VAT_CM3	0.0000549319489027089	0.000012382355443839	0.0000108915405143277
		Female	0.21061338971306	0.0326729016255463	2.3624915827945E-10
		BMI	0.00711705268065337	0.00446049554545465	0.111111269960812
		Age at CT scan	-0.00174607502539682	0.00142077528165995	0.219569630405629
III	DNAmlogCRPAdjAge	SAT_CM3	0.0000320524594988421	0.0000159819165804588	0.0453555604941175
		SAT_HU	0.00851327102506476	0.00427687493696753	0.0469874908294514
		VAT_CM3	0.0000437656295960282	0.0000194657512869669	0.024918505461753
		VAT_HU	-0.00494246127130323	0.00413771126527481	0.232760084235606
		Female	0.220732514611499	0.0340409966410324	1.87007287920982E-10
		BMI	0.00514539586289426	0.00458830461081485	0.262561906328698
		Age at CT scan	-0.00213946455128356	0.00145732380758355	0.14260977827154
IV	DNAmlogCRPAdjAge	LIVER	-0.00652565473064705	0.00146822997100933	0.0000108119617565713
		SPLEEN	0.00354378934615982	0.00287020416541773	0.217517443301476
		MUSCLE	-0.000904409619672705	0.00193971730671087	0.641229585826029
		SAT_CM3	0.0000201274044847075	0.0000162298017621343	0.215489821931786
		VAT_CM3	0.0000364285045369923	0.0000144333453528689	0.0119082147047212
		Female	0.224349787525412	0.0365489444612348	1.67705762083865E-09
		BMI	0.00540326213183127	0.00481296824459281	0.26211587010049
		Age at CT scan	-0.00145663212648968	0.00162319617077255	0.369936859405074

Supplementary Table 3.5. Multivariate regression analysis of DNAmlogA1CAdjAge on CT-scan derived fatty liver and adipose tissue density in FHS.

Model	Y	X	Beta	SE	P
I	DNAmlogA1CAdjAge	LIVER	-0.000886026145980581	0.000104515193453869	2.36931537104274E-16
		SPLEEN	0.0000539545090005328	0.000208681172918216	0.79608440974091
		MUSCLE	-0.0000863308257918765	0.000144312594205194	0.549951938534319
		Female	0.00405851826142004	0.00193541896672057	0.0364766380341877
		BMI	0.000397450587047914	0.000249553164556365	0.111844292206913
		Age at CT scan	0.0000479742781959997	0.000123061465613299	0.696813893289029
II	DNAmlogA1CAdjAge	SAT_CM3	-6.95761606082695E-07	1.10853729924978E-06	0.530479425152961
		VAT_CM3	7.58779757829765E-06	9.44120867660531E-07	4.92559999606763E-15
		Female	0.0126329755006024	0.00248770020810905	5.09585747438412E-07
		BMI	0.000287382014295494	0.000340019110863444	0.398340159071714
		Age at CT scan	-0.000136011233502378	0.000109502327109008	0.214691163753782
III	DNAmlogA1CAdjAge	SAT_CM3	8.48946007625696E-07	1.21022613490267E-06	0.483278625204431
		SAT_HU	0.00136184271902832	0.000323838785763361	0.0000300728268376565
		VAT_CM3	3.91453755062786E-06	1.47226301375199E-06	0.00805090738099513
		VAT_HU	-0.00124305231457744	0.000312986705597368	0.000080110639284532
		Female	0.0131916675535521	0.0025704731474462	0.0000003883233966291
		BMI	0.0000308550730229477	0.000347028177090041	0.929181427163583
		Age at CT scan	-0.000161614306873522	0.000111303689050094	0.147023550524075
IV	DNAmlogA1CAdjAge	LIVER	-0.000706010410787347	0.000111258710493928	4.89407595344815E-10
		SPLEEN	0.000130584499919972	0.000217298775464703	0.548143946031757
		MUSCLE	-0.000100052075614054	0.000146674601482737	0.495462423372083
		SAT_CM3	-1.18094156504028E-06	1.23112588499746E-06	0.337893861513122
		VAT_CM3	6.02335217277869E-06	1.09265587636537E-06	5.62401973827812E-08
		Female	0.013255014929255	0.00276426806108236	2.13670536595381E-06
		BMI	0.0000148746940111846	0.000364766907404142	0.967488336247237
		Age at CT scan	-0.000105767228089239	0.00012421602671623	0.394903441179087
